# Abstracts DGCH

**DOI:** 10.1515/iss-2019-2002

**Published:** 2019-03-20

## DGCH: Medical education / Medical training

### Learning curve of surgical novices using the Single Port Platform SymphonX – Minimizing OR trauma to only one 15mm incision

(Abstract ID: 179)

R. Datta^1^, S. Schönhage^1^, D. Dratsch^1^, S.-H. Chon^1^, R. Wahba^1^, F. Gebauer^1^, G. Dieplinger^1^, D. Stippel^1^, R. Kleinert^1^, C. J. Bruns^1^, P. Plum^1^, H. Fuchs^1^

^1^*Uniklink Köln*

**Background:**

Minimally invasive single port surgery was associated with large incisions up to 2-3cm, complicated handling due to the lack of triangulation, and instrument crossing. Recently, the new surgical platform SymphonX has been introduced to the market, eliminating these disadvantages.

Aim of this prospective single center study was to evaluate the learning curve of medical students using a new surgical platform that allows for triangulation incorporating robotic features and that can be introduced through only one 15mm trocar.

**Materials and methods:**

The new technology has been introduced into clinical practice at our academic center as first European site after FDA and CE clearance.

A set of 5 laparoscopic skill tests (Rope Pass, Papercut, Peg Transfer, Recapping, needle thread) were performed with 3 repetitions. Medical students performed all tests with both conventional laparoscopic instruments and the new platform. Time and errors were recorded.

**Results:**

A total of 114 surgical novices (61 females) with a median age of 23 years completed the study. All students were able to perform the skill tests with both conventional and single port laparoscopic systems.

There was no significant difference in the learning curve and error rate for each skill test. In some tests, there was e tendency of a lower error rate using the SymphonX platform

**Conclusion:**

The learning curve of surgical novices using the new surgical platform SymphonX is comparable to standard laparoscopy in this large series. The error rate is promising. Further studies evaluating the new technology is ongoing.

**Picture:**
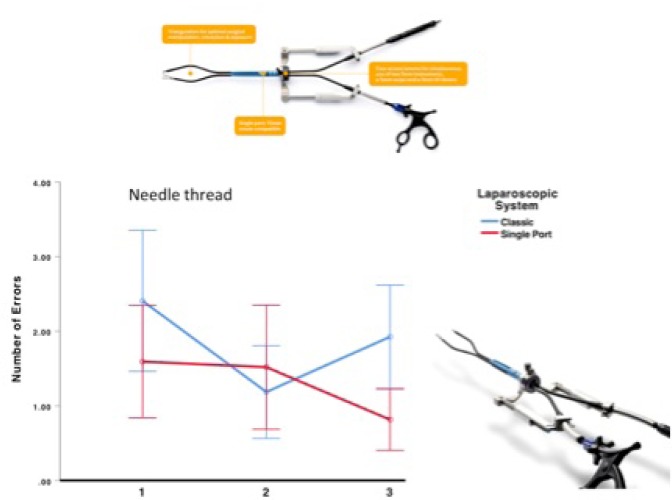
Fortimedix System

### Influential factors on the outcome of a basic Minimal-Invasive-Surgery training – Creating a Curriculum for medical students and surgical residents

(Abstract ID: 210)

T. Hoffmann^1^, V. Kimmerling^1^, M. Dürsch^2^, H. J. Schlitt^1^, M. Hornung^1^

^1^*Universitätsklinikum Regensburg*

^2^*St. Vincenz-Krankenhaus, Paderborn*

**Background:**

Minimally invasive surgery (MIS) became very popular among surgical procedures over the past two decades. The technique can be trained very well on a variety of simulators, however many surgical curriculums at medical schools and surgical residency programs still lack a structured, hands-on training. This study’s aim is to identify influential factors to create a curriculum for novices to MIS, to increase interest in a surgical career and enhance MIS-skills amidst surgical residents.

**Materials and methods:**

The study included 200 men and women with no prior experience in the field of MIS. Participants were randomized into subgroups, completing the training alone or in pairs. They completed a four-step basic MIS-training on a box simulator. Surveys were conducted before and after every session. Performance was assessed through completion time improvement. Analysis of covariance (ANCOVA) was used for statistical evaluation.

**Results:**

Subgroup composition had no effect on completion time improvement. There were significant differences in the training results among subgroups divided by the number of errors, men (p=0,001), women (p= 0,001) and number of changes between hands, men (p<0,001), women (p=0,002). Moreover, the execution of cognitive exhausting tasks prior to training was significantly beneficial, men (p=0,019), women (p=0,043). Female participants who watched their team partner complete the task, advanced significantly more than females who had no interest in their teammate’s work. (p=0,017). Male participants with a strong subjective spatial sense had a significantly better completion time improvement (p=0,013). Supporting the team member in the all-male teams or mixed gender teams also proved to be of significance (p=0,014).

**Conclusion:**

A clear impact of team composition could not be found. However, there are influences that need to be considered when constructing a MIS-training. It is a valuable addition to surgical curricula and should be incorporated in already existing surgical programs. It seems reasonable to give a short lecture of instructions on technique and teamwork before starting the training. In addition, giving participants a clear framework as well as encouraging mutual support during sessions will improve the trainees’ performance.

### Serious Games in surgical medical education: A virtual Emergency department as a Tool for Teaching Clinical Reasoning in Surgery

(Abstract ID: 235)

R. Kleinert^1^, R. Datta^1^, S.-H. Chon^1^, C. J. Bruns^1^

^1^*Uniklinik Köln*

**Background:**

Serious Games enable the simulation of daily working practices and constitute a potential tool for teaching both declarative and procedural knowledge. The availability of educational Serious Games offering a high-fidelity, three-dimensional environment in combination with profound medical background is limited, and most published studies have assessed student satisfaction rather than learning outcome as a function of game use.

It was our aim to test the effect of a Serious Game simulating an accident & emergency department ("EMERGE") on students’ declarative and procedural knowledge as well as their satisfaction with the serious game.

**Materials and methods:**

140 medical students in the clinical part of their training (5th Semester to PJ (practical year)) self-selected to participate in an experimental study. Declarative knowledge (measured with 20 multiple choice questions) and procedural knowledge (measured with written questions derived from an OSCE station) were assessed before and after working with EMERGE. Students’ impression of the effectiveness and applicability of EMERGE were measured on a 6-point Likert scale.

**Results:**

A pre-post comparison yielded a significant increase in declarative and procedural knowledge. The effect on declarative knowledge was larger in students in earlier years of education than in students of higher semesters. Additionally, students’ overall impression of EMERGE was positive.

**Conclusion:**

The current study reveals that working with a Serious Game, such as EMERGE, has a positive effect on declarative and procedural gain and can may be used as an additional tool for teaching medical students. Future studies should investigate what specific aspects of playing EMERGE lead to the reported effects.

### Immersive anatomy atlas – Randomized comparison of the learning success in an analog and virtual learning environment

(Abstract ID: 309)

D. Weyhe^1^, V. Uslar^1^, F. Weyhe^1^, M. Kaluschke^1^, G. Zachmann^1^

^1^*Universitätsmedizin Oldenburg, Pius-Hospital, Oldenburg*

**Background:**

Research is increasingly being conducted towards the potential of Virtual Reality (VR) technology as a tool in education at schools and universities. In this context, we developed a prototype of a virtual anatomy atlas for surgical anatomical training and further education. The aim of this study was to evaluate the difference in learning outcome between this digital teaching medium and a conventional "open book" method (OB) in randomized students of the 11th grade of two German high schools under exam conditions.

**Materials and methods:**

A total of 28 students divided into two Groups were asked nine anatomy questions. One group used conventional anatomy books and charts to answer the questions below. The other group used the VR Anatomy Atlas.

How many lobes does the right lung have? What is the structure between stomach and lungs? Name the annular muscle that surrounds the eye Name the Latin term of the kneecap Name the nerve structure connecting the brain to the spinal cord How many muscles are in direct contact with the femur? How many parts does the calf muscle consist of? Where is the thyroid gland? In front of or behind the windpipe? What is the right temporal muscle (in German: "Schläfenmuskel") called in Latin? Sketch the Achilles tendon in proportion to the leg To measure the success for each learning method, the error rate, the processing speed for the individual questions (stratified for school affiliation), the satisfaction with the teaching unit and the existence of a medical career wish were evaluated.

**Results:**

The error rate was the same for both schools and between both teaching aids (VR: 34.2%; OB: 34.1%). The answering speed for correctly answered questions in the OB group was approx. twice as high as for the VR group (mean value OB: 98sec, range: 2-410sec; VR: 50sec, 1-290sec). There was also a significant difference between the students of the two schools based on a longer processing time in the OB condition in School B (mean OB in School A: 158sec; OB in School B: 77sec). The subjective survey on the learning methods showed a significantly better school grade for VR (p = .012). Medical career aspirations have been strengthened with VR, while interest in the OB group has tended to decline.

**Conclusion:**

We conclude that the immersive anatomy atlas helped the subjects to actively and intuitively perform targeted actions that led to correct answers even without prior knowledge of VR and anatomy. With the OB method, orientation difficulties and/or the technical effort in the handling of the topographical anatomy atlas generally seem to lead to a significantly longer response time, especially if the students are not specially trained in literature research in books or texts. This seems to indicate that the VR environment in the sense of constructivist learning might be a more intuitive and easier to use learning environment than the more traditional acquisition of knowledge from books.

**Picture:**
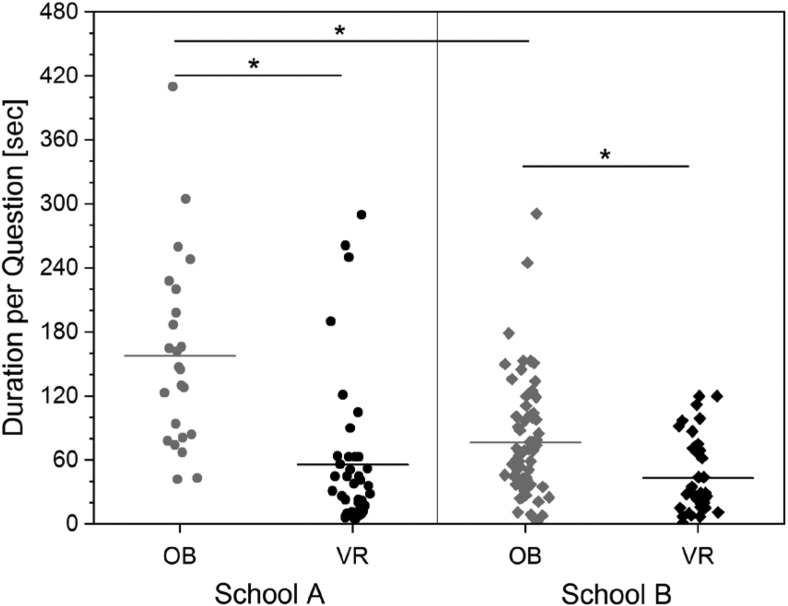
Processing time for correctly answered questions depending on school affiliation and teaching conditions. The individual measured values and the average value per group are shown.

### Situation awareness in multimodal laparoscopy training enhances performance of laparoscopic procedures – a randomized controlled trial

(Abstract ID: 477)

L. Seifert^1^, K.-F. Kowalewski^1^, M. W. Schmidt^1^, K. F. Köppinger^1^, B. P. Müller-Stich^1^, F. Nickel^1^

^1^*Universitätsklinikum Heidelberg*

**Background:**

Minimally Invasive Surgery (MIS) is standard for many indications, especially for basic procedures such as cholecystectomy or appendectomy. Benefits for the patients are undoubted but surgeons face new challenges (e.g. restricted vision and degrees of freedom). Therefore, preoperative training has become mandatory in surgical education. While most curricula focus on psychomotor skills, recent evidence underlines the importance of cognitive training. Situation awareness (SA) is a cognitive skill of effective information processing. It depends on common cognitive abilities, such as attention, (working)memory and multitasking. SA can be divided into three steps 1) perception of clues in the environment 2) connection of clues with personal knowledge for understanding their relevance 3) prediction of possible outcomes of the situation. Good SA is required for adequate decision-making and avoidance of complications. This study aimed to evaluate the potential benefit of SA training in surgical education.

**Materials and methods:**

A prospective, single-centre, two-arm, parallel-group randomized trial was conducted. Laparoscopically naïve medical students in their clinical years (3rd-6th year) were randomized in two groups and received either e-learning-based SA training or standard e-learning. After standardized introduction and basic skill training, students performed a laparoscopic cholecystectomy (LC) on a cadaveric porcine model as a baseline test. LC performance was measured with the Objective Structured Assessment of Technical Skills (OSATS) and Global Operative Assessment of Laparoscopic Skills (GOALS) by trained and blinded raters. Both groups watched 212 videoclips showing key steps of LC: 1) differentiation of artery, duct and connective tissue, and 2) gall bladder preparation. In order to improve SA, the videos of the intervention group stopped suddenly and participants were asked to predict an upcoming event or to name anatomical structures. The control group watched the videos without interruption for questions. After intervention, another LC was performed as a post-test assessment.

**Results:**

A total of 50 students were included. Baseline test revealed no differences between groups. There were no significant differences in terms of GOALS score (18.5±4.6 vs 18.7±5.4; p=0.981), OSATS score (62.2±12.8 vs 57.3±9.4; p=0.348) or operative time (64.5±22.0 vs 62.8±15.8; p=0.762). There were correlations between self-assessed attention and GOALS score (R=0.15; r^2^=0.021; p=0.554), OSATS score (R=0.21; r^2^=0.033; p=0.46) and time (R=-0.08; r2=0.007; p=0.734). Correlations between the questions answered correctly in the video clips and performance were shown for GOALS (R=0.11; r2=0.012; p=0.649) and OSATS (R=-0.26; r^2^= 0.068; p=0.281), respectively. The evaluation of the training showed that both groups considered the intervention as useful while the intervention group rated their e-learning higher in usefulness (70.5%±13.39% vs 56.25%±23.26%; p=0.123) and learning effect (72.5%±23.26% vs 55.63%±16.13%; p= 0.032) than the control group.

**Conclusion:**

Participants of both groups considered SA training beneficial but it did not result in improved LC performance. Further studies should focus on improving SA training based on the findings and evaluate whether SA training is more helpful for advanced procedures requiring mental workload.

### The EduDerm Skin Model: Development of a high-fidelity skin model for medical education according to the ADDIE instructional design model

(Abstract ID: 532)

S. Schmitz^1^, H. Schröder^1^, A. Andert^1^, A. Röth^1^, C. Klink^1^, U. P. Neumann^1^, N. Steuer^1^, C. Luisi^1^, L. Strudthoff^1^, M. Holtsträter^1^, U. Steinseifer^1^, G. Wagner^1^, J. Arens^1^, S. Sopka^1^

^1^*Uniklinik RWTH Aachen*

**Background:**

Mastering basic surgical skills such as suturing and knot tying needs sufficient training time and equipment. With medical curricula becoming more and more complex, usually they do not contain enough time for learning basic surgical skills. Suturing is frequently practiced on animal skin, which is for single-use only, contains ethical concerns and is not easily available to practice at any place. First objective of this study was to conduct a needs-analysis among students to assess the requirements for a take-home suturing model. Afterwards the results led to the design of the EduDerm skin model according to the ADDIE instructional design model (Analysis, Design, Development, Implementation, Evaluation). The project is carried out in cooperation with the Department of Cardiovascular Engineering, Institute of Applied Medical Engineering (CVE/AME), RWTH Aachen University, where the EduDerm skin modell is developed and manufactured.

**Materials and methods:**

All medical students were contacted via mail for a voluntary survey on surgical education. Self-perception of basic surgical skills was assessed as well as number of voluntary visits of our training center AIXTRA. Students’ needs of different model features were assessed. EduDerm Skin Models were developed and manufactured at the CVE/AME according to students’ demands. Funding was approved by the medical faculty’s’ grant for innovative educational projects. Pilot implementation was conducted in our clinic’s suturing course accompanying clinical internships and evaluation was performed after 90 minutes of practicing. For evaluation Likert scales ranging from 1 to 6 were applied.

**Results:**

The respondents (n=204) did not feel well-prepared for a surgical internship (mean 1.88, SD 1.4) and teaching of basic surgical skills was demanded to get more representation in the curriculum (mean 4.2, SD 1.2). Students’ request for a take-home skin model was high (mean 5.53, SD 0.82) and the EduDerm model was rated as applicable (Likert scale: cutting: mean 5, SD 1.22; intracutaneous suturing: mean 5.1, SD 1.0; deep suturing: mean 4.4, SD 1.4). Students’ basic surgical skills improved significantly after practicing with the EduDerm Skin Model (Likert scale: continuous intracutanous suturing: 3.464 vs. 1.866, single knot suturing: 4.25 vs. 2.412, Donati’s suture: 3.179 vs. 1.809, Allgöwer’s suture: 2.929 vs. 1.483, instrumental knot tying: 4.25 vs. 2.21, hand knot tying: 3.857 vs. 2.044; all p-values <0.001).

**Conclusion:**

Medical students of all semesters at RWTH reported a need for training of basic surgical skills. With already full curricula a take-home model for independent training met a wide acceptance.

According to the ADDIE model of instructional design, analysis, implementation, and evaluation were performed by the AIXTRA, UKA, and the development and design of the model was performed by the CVE/AME tailored to students’ demands. The model was applicable and students felt more confident in basic surgical skills after practicing with the EduDerm Skin Model.

### Prospective randomized study evaluating the impact of intraoperative teaching on surgery duration and workload of the surgeon

(Abstract ID: 585)

T. Aumann-Münch^1^, B. Sahlmann^1^, S. Janssen^1^, V. Uslar^1^, D. Weyhe^1^

^1^*Universitätsmedizin Oldenburg, Pius-Hospital, Oldenburg*

**Background:**

During the clinical internships of medical students, students are regularly placed in the operating theatre. A structured curriculum for intraoperative clinical training is not available at most hospitals and university clinics. Clinical skills are taught either situatively or in clinical training centres outside the operating theatre. Nevertheless, the students spend a substantial part of their internship in the operating theatre and are part of the surgical team. Here they can gain insights into surgical procedures and techniques and refresh their anatomical knowledge, but curricular training by the surgeon usually does not take place or takes place very individually. We are not aware of any studies on the additional stress situation of the surgeon due to structured intraoperative training. This study is intended to compare the workload of surgeons with and without structured intraoperative teaching. In addition, quality and success of intraoperative clinical teaching are evaluated from the students’ point of view.

**Materials and methods:**

The prospective randomized study was initiated after a positive ethics vote. Over a period of 9 months, either thyroid gland resection, laparoscopic cholecystectomy (CHE) or inguinal hernia surgery (TEP) was performed on n=90 patients, where a medical student was present in the operating theatre. Group A (n=43) received an intraoperatively structured teaching and group B (n=37) operated only the instruments required for situs control. Biometric data of the patients, the surgeons, type and extent as well as intraoperative peculiarities of the performed operation were recorded and documented by a study nurse. For the surgeons, there was no obligation intraoperatively to carry out the teaching units and they were also able to end the teaching unit in stress situations. After the operation, the subjective workload of the surgeons in the operating room was evaluated by the NASA-TLX Score as well as the teaching quality from the student’s point of view.

**Results:**

For the operations recorded, the average duration of the teaching-related OP conversations in group A was 7.5 minutes. (range: 0 - 26min). In group B, the duration of intraoperative communication was 3.3 minutes (range: 0 - 23min). In group A there was a significantly longer operation time for the lap. CHE and TEP (CHE= 49 min group B vs. 78 min group A; TEP: 40 min vs. 54 min). For thyroid gland resections, no influence on the cut/suture time could be found (82 vs. 81 min). The workplace exposure measured with the NASA-TLX was higher in the dimensions "mental, physical and temporal demands", as well as in the estimated "effort" and "frustration" in group A than in group B. (Fig. 1).

**Conclusion:**

Structured intraoperative teaching increases the workload of the surgeon and also prolongs the operation time. This effect of intraoperative teaching seems to be particularly important in minimally invasive surgery. Larger studies should validate these observations and, if necessary, develop pre- and postoperative training concepts to reduce the intraoperative burden on the surgeons. The influence of intraoperative teaching on patient safety is unclear.

**Picture:**
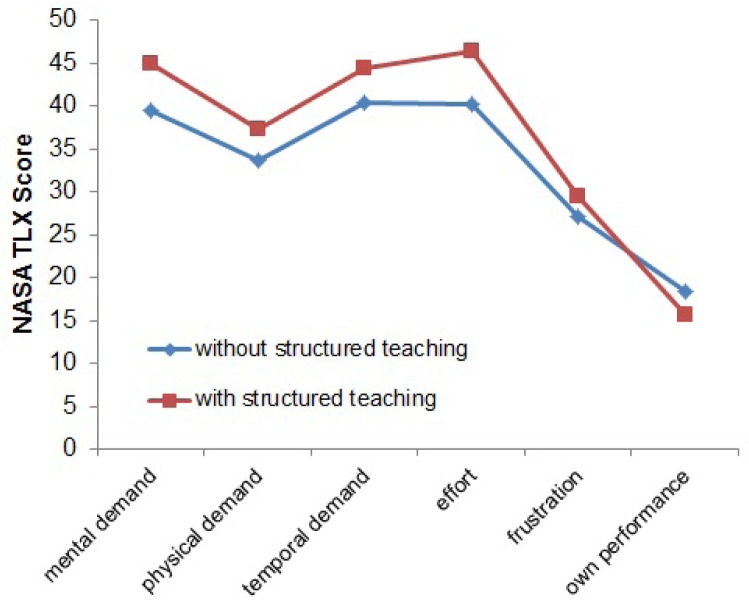
mean of the NASA-TLX dimensions for the group with and without strucured teaching

### Establishing a structured doctoral program for medical doctoral degrees in the Department of Surgery at the University Hospital Heidelberg

(Abstract ID: 659)

M. Skunde^1^, T. Wiedenmann^1^, M. Friedrich^1^, A. L. Mihaljevic^1^

^1^*Universitätsklinikum Heidelberg*

**Background:**

Physicians not only need medical knowledge but also scientific competences to be able to find the best treatments for their patients at all times. However, curricula for medical studies in Germany often focus very little on the training of scientific skills with the doctoral thesis usually being the only scientific project during the course of medical studies. We are aiming to improve the scientific expertise of medical students with a structured program for obtaining a medical doctoral degree in the Department of Surgery at the University Hospital Heidelberg.

**Materials and methods:**

The doctoral program supports students of medicine and dental medicine during their doctoral thesis work in the Department of Surgery with a structured curriculum. All participants regularly take part in Journal Clubs and workgroup meetings to promote scientific exchange among students and their supervisors. Furthermore, participation in a seminar of good scientific practice and in a scientific conference is compulsory for all students. Further courses (experimental techniques, statistical analysis, animal handling, scientific writing, etc.) must be chosen according to interests and specific needs of the doctoral thesis project. To help with successful planning of the project, to give additional scientific advice, and to encourage critical discussions about the project, a thesis advisory committee, consisting of the project supervisor and two additional researchers, accompanies every student during the work on his doctoral thesis. Students register for participation in the doctoral program at the beginning of their research work. During the registration process, students are asked about the general frameworks of their doctoral thesis, such as planned total duration, amount of time dedicated to full-time research work or motivation for joining the doctoral program, and the answers are analyzed.

**Results:**

Between October 2016 and September 2018 85 students registered for participation in the structured doctoral program. The participants expect an average duration of research work of 17 ± 6 months (mean ± SD) until completion of their doctoral thesis. Most participants choose experimental research projects (61%), followed by clinical research projects (21%). Research projects in the field of medical education were chosen by 11% of students, followed by projects in the field of minimally invasive surgery (7%). 39% of registered students plan to make use of the possibility to interrupt their medical studies for one semester to be able to work on their research project for 6 months full-time. 56% are willing to work more than 6 months full-time, therefore pausing their studies for more than one semester. Only 5% of participants enrolled in the doctoral program exclusively work part-time or less than one semester full-time for their doctoral degree. Students name "Access to training workshops" and "Scientific exchange" as the main reasons for registering to the doctoral program.

**Conclusion:**

A structured doctoral program attracts mostly enthusiastic students with ambitious research plans. Further follow-up is needed to determine if participating students benefit from a better performance during their doctoral thesis, e.g. determined by publication success or results from doctoral thesis evaluations.

### PATRONUS – The first student-lead clinical trial in Germany

(Abstract ID: 696)

M. Friedrich^1^, M. Schwab^1^, A. L. Mihaljevic^1^

^1^*Universitätsklinikum Heidelberg*

**Background:**

Evidence should define and guide modern clinical care, yet many relevant questions in surgical practice remain unconfirmed by data. Meaningful clinical research however is challenging to conduct and its overall infrastructure in Germany was - until recently - considered poor as compared to other leading countries. While this has been significantly improved following the establishment of the Studienzentrum der Deutschen Gesellschaft für Chirurgie (SDGC) and the surgical clinical trial network CHIR-Net, limited focus has been put on training and recruitment of medical students to become competent clinician scientists.

**Materials and methods:**

To address these challenges, the CHIR-Net has established a student-initiated clinical trial network (SIMGA; Student-Initiated German Medical Audit) in 2017. Inspired by initiatives from the United Kingdom, this network enables students to participate in academic research projects and serves as exchange platform between students and physicians. As part of the SIGMA network, students contribute to national multi-center trials while improving clinical and research skills and gaining an insight into clinical academia. PATRONUS (Multicenter prospective cohort study of PATient-Reported Outcomes and complications following major abdominal Neoplastic Surgery) is the first SIGMA-initiated project and the first-ever student-lead clinical trial in Germany.

**Results:**

SIGMA achieved the following objectives:

- Creation of a national multicenter network of medical students and associated clinician scientists (n=15 established centers)
- Training of medical students in methodology, regulatory affairs and ethical conduct (SIGMA Prüfstudierendenkurs, n=32 trained participants).
- Design, initiation, conduction, analysis and publication of prospective multicenter clinical trials initiated by medical students (PATRONUS, n=351 recruited patients)

**Conclusion:**

SIGMA is a product of strong collaboration between clinical scientists and medical trainees, enabling students to contribute to high-quality clinical trials. Additionally, participants are offered extensive training to support the next generation of research-active clinicians. PATRONUS is the first-ever student-lead clinical trial in Germany and successfully recruited 351 patients. Preliminary data of patient follow-up will be available by early 2019.

## DGCH: Surgery 4.0

### The role of the Da Vinci robotic system in revisional bariatric surgery

(Abstract ID: 73)

U. Hesse^1^, J. Lenz^1^, M. Vladimirov^1^, H. J. Stein^1^

^1^*Klinikum Nürnberg Paracelsus Medizinische Privatuniversität, Nürnberg*

**Background:**

The arrival of robotic assisted surgery in the treatment of morbidly obese patients has enlarged the armamentarium for surgeons involved in bariatric surgery.

While the implementation of the technique has faced many hospitals with increasing costs and training the advantages of the technical development have been demonstrated in several recent studies. Three dimensional optical view and the endowrist technique are two main features of the system facilitating the surgeons capability in intraabdominal dissection and suturing and increasing the exposition of the operational field due to the instrument holding arms and a third hand for the console surgeon.

This in particular is of great advantage not only in primary cases but also in patients undergoing revisional procedures following preceeding upper GI surgery.

Aim: In the following the experience with revisional surgery using the Da Vinci robotic system will be reported and compared to conventional laparoscopic treatment and the literature.

**Materials and methods:**

Patients and methods: In a 12 months period a total of 59 minimally invasive bariatric procedures (20 robotic assisted, 39 laparoscopic) were performed. 14 patients received a gastric bypass, 31 a gastric sleeve and in 14 patients a band was removed or adhesions were resected without an alternative procedure. Out of the 14 GBP procedures 8 (58%) were performed robotic. Out of these 4 (50%) had previous operations 1 hiatal mesh repair, 1 open Mason operation, 1 gastric band, 1 gastric sleeve. The Da Vinci Xi was used for the surgery.

**Results:**

Results: All these patients resumed oral nutrition on day 2 post OP and were discharged from the hospital 4,4,4, and 7 (pneumonia) days post OP. No patient had to be reoperated. The average operating time was 194,5 min (142-228) vs 290,2 min (154-480) of the remaining.

**Conclusion:**

Conclusion: This preliminary experience suggests that robotic revisional surgery can be performed safely even in complicated cases and with a short learning curve.

### Telephone call management systems: Is an intelligent operating room system able to reduce unnecessary stress factors during an operation?

(Abstract ID: 511)

N. Samm^1^

^1^*Klinikum Rechts der Isar der TU München*

**Background:**

Mobile phones increase reachability of a surgeon and improve patient’s care. But interruptions of the surgical workflow in the OR due to incoming calls influence the surgeon’s concentration and therefore have a negative impact on patient safety.We evaluated if a cognitive OR system could be used for a situation-adapted preselection of incoming calls for the surgical team.

**Materials and methods:**

46 volunteers were confronted with three call type scenarios of different urgency in combination with different OR situations and time lines. Tree different types of caller scenarios with a graded importance for patient care were given. For each call type scenario the callers were informed about the approximate remaining operating time (60, 30 or 15 minutes) and the current situation in the OR (normal, irregular, critical).

**Results:**

For minor inquiries only 1% of all participants confirmed to forward the call, for major inquiries, 7% of all calls were routed to the surgeon. For vital inquiries 78% of all test persons opted for a forwarding. With a remaining operating time of 15 minutes, only 37% of the participants decided to be forwarded in case of a vital inquiry. Results did not show a correlation between increasing message severity for different OR situations (normal, irregular, critical) and the percentage of calls being forwarded.

**Conclusion:**

Results indicate a statistically significant correlation between the importance of the call and the number of calls being forwarded. Emergency calls are largely transmitted by the system while less important calls are blocked. The different OR situations had no clear influence on the percentage of forwarded calls.

### Implementation of Acute Care Surgery at a University Hospital in Switzerland: First 2-year Experience

(Abstract ID: 565)

B. Schnüriger^1^, S. Winterhalder^1^, T. Haltmeier^1^, D. Candinas^1^

^1^*Universitätsspital Bern*

**Background:**

Specialized surgical teams have been shown to improve outcomes in multiple elective patient populations. Starting in the USA ten years ago, Acute Care Surgery (ACS) is currently evolving worldwide. ACS focusses on patients with acute surgical disease, including traumatic and non-traumatic emergencies. At an academic, tertiary referral center, an ACS service has been established in 2016. Here we report our experience two years after the implementation of the ACS service.

**Materials and methods:**

Retrospective descriptive analysis of a prospectively collected database including all patients that underwent emergency abdominal surgery 01/2016-12/2017.

**Results:**

During the 2-year study period, a total of 1106 patients underwent emergency abdominal surgery. Of these, 79.3% were admissions to the Emergency Department (ED) and 20.7% were already hospitalized for other diseases. Overall, 65.7% of the patients were admitted from home and 27.1% from other hospitals. Median age was 57.0 years (IQR 31). Most frequent indications for surgery were appendicitis (17.0%), diseases of the colon (14.0%), diseases of the gallbladder (12.6%), hollow-viscus perforations (10.7%), small bowel obstruction (7.0%), bleeding (4.4%), and mesenteric ischemia (3.6%). Admission to the Intensive Care Unit (ICU) was required in 28.4% and 33.5% were admitted to the Intermediate Care Unit. In-hospital mortality was 6.7% and median length of stay 7.0 days (IQR 14).

**Conclusion:**

During the first two years after the establishment of an ACS service, surgery has been performed for multiple abdominal emergencies, both in patients admitted from home and patients that were already hospitalized. The ICU admission rate of 28.4% reflects the high grade of physiologic derangement in ACS patients. In times of increasing subspecialization, dedicated ACS services with broad knowledge and skills in the management of patients with acute surgical disease may provide high-quality of care in this patient population with extensive treatment needs.

## DGCH: Surgery and economy

### Physicians’ views on pay-for-performance as a reimbursement model: a quantitative study among Dutch surgical physicians

(Abstract ID: 190)

K. Alqasim^1^, E. Ali^2^, S. Evers^1^, M. Hiligsmann^1^

^1^*Charité - Universitätsmedizin Berlin - CVK*

^2^*Memorial Hermann Hospital, Houston*

**Background:**

Aim of the study is to assess the views, knowledge, and experience of Dutch physicians with regard to the general objectives and values of the pay-for-performance (P4P) system, as the Dutch healthcare industry might find it useful, in terms of governance and health economy, to explore this approach further, compared to the standard DRG system.

**Materials and methods:**

A quantitative cross-sectional survey study was conducted among 48 physicians in surgical specialties in the Netherlands between May 2014 and July 2014. The survey questionnaire was designed to gather information regarding the intensity of feelings, on a 7-point Likert scale, toward statements that address the P4P system. Confidence intervals were calculated using the bootstrap technique with 1000 iterations.

**Results:**

Physicians see a positive value in P4P for their organizations rather than for personal attainment (mean = 5.00; 95% CI = 4.62-5.39), even though they feared that P4P might put financial pressure on them (mean = 5.03; 95% CI = 4.50-5.54). They strongly share the view that other colleagues will resist adopting P4P as a business model (mean = 5.74; 95% CI = 5.43-6.04). Respondents stated that they would not leave their current jobs if P4P were to be incorporated in their organization.

**Conclusion:**

Physicians see value in P4P for their organizations, and consider that P4P could provide an incentive for improving medical outcomes compared to the standard DRG system. There seems to be potential for the P4P system as participants expressed positive support for its values. There is an intersection of interests between the value of P4P and the physicians’ aim of achieving quality outcomes; however, further studies would be needed to investigate perceptions about specific design features in a larger sample. In addition, prior to implementing P4P, broad education about the system should be provided in order to counteract pre-conceptions and prevent resistance.

## DGCH: Research & Studies

### Remote Ischemic Conditioning (RIC) – Improving a non-invasive technique of tissue conditioning

(Abstract ID: 29)

A. Sogorski^1^, J. Kolbenschlag^2^, S. Spindler^1^, M. Dostibegian^1^, B. Behr^1^, K. Harati^1^, M. Lehnhardt^1^, M. Dadras^1^

^1^*Universitätsklinikum BG Bergmannsheil Bochum*

^2^*BG Klinikum Tübingen*

**Background:**

Remote Ischemic Conditioning (RIC) is a non-invasive method of tissue conditioning. Over the last years it has been demonstrated as a promising technique to render tissues and organs resilient against prolonged ischemic events and the ischemia/reperfusion injury in a variety of surgical fields. Furthermore, RIC has been shown to improve cutaneous microcirculation of e.g. free or pedicle fascio-cutaneous and myo-cutaneous flaps. Through its remote mode of action there are no anatomical barriers or limitations between the conditioning site (e.g. upper limb) and the target organ (e.g. free flap transplanted to the lower limb).

The aim of our prospective clinical study was further optimization of the RIC technique with regard to its effect on cutaneous microcirculation. We focused on the impact of a different number of applied conditioning cycles as well as the duration of changes.

**Materials and methods:**

In a prospective randomized clinical study perfusion changes as a result of the RIC treatment were investigated in 80 young, healthy subjects. The RIC stimulus as applied at the upper limb through inflation/deflation of a surgical tourniquet. One RIC cycle consisted of 10 minutes of ischemia followed by 10 minutes of reperfusion. The applicated number of cycles was 1, 3, 5 or 7, respectively. Cutaneous microcirculation was assessed continuously at the antero-lateral thigh. Combined laser-doppler flowmetry and white-light spectroscopy was used to analyze cutaneous blood flow, tissue oxygen saturation and relative hemoglobin content during the conditioning. Measurements were continued for 4h after the last applied cycle to assess the duration of microcirculatory changes.

**Results:**

RIC caused significant (p<0.05) changes of superficial and deep cutaneous microcirculation. Tissue oxygen saturation increased due to an improved blood flow. Group II with application of 3 cycles showed most pronounced changes whereas a higher number of cycles did not lead to a further increase of measured parameters. Overall, changes were more pronounced in superficial layers than in the deep subdermal level. Sustainable (p<0.05) microcirculatory improvement was present for at least two to four hours after the RIC stimulus.

**Conclusion:**

RIC is a simple non-invasive technique to enhance cutaneous microcirculation. Due to its remote character application is safe, because tissues at risk are not directly subjected to certain ischemic events. A conditioning protocol of 3 cycles, each consisting of an interval of 10 minutes of ischemia followed by 10 minutes of reperfusion, appears most reasonable with regard to its beneficial effect on cutaneous microcirculation.

### Bilirubin, urobilinogen, pancreas elastase and bile acid in drain fluid. The GBUP-study: Analysis of possible predictive biomarkers for a colorectal anastomotic leakage

(Abstract ID: 33)

C. Paasch^1^, S. Rink^1^, M. Steinbach^1^, S. Kneif^1^, D. Peetz^1^, A. Klötzler^1^, U. Gauger^2^, K. Mohnike^3^, M. Hünerbein^1^

^1^*HELIOS Klinikum Berlin-Buch*

^2^*Freier Statistiker, Berlin*

^3^*Universitätsklinikum Magdeburg*

**Background:**

A colorectal anastomotic leakage (CAL) is a major complication after colorectal surgery and leads to high rates of morbidity and prolonged hospital stay. The study aims to evaluate the benefit of using bilirubin, urobilinogen, pancreas elastase and bile acid in the drain fluid (DF) as a predictive marker for the CAL.

**Materials and methods:**

From June 2015 to October 2017 100 patients, who underwent left hemicolectomy (LH), sigma resection (SR), high anterior resection (HAR), low anterior resection (LAR) or reversal of Hartmann´s Procedure (ROHP) were included in this monocentric non-randomized prospective clinical trial. During the first four postoperative days (POD) the concentration of bilirubin, urobilinogen, pancreas elastase and bile acid in the DF was measured.

**Results:**

In total 100 patients were recruited. 17 were excluded due to intraoperative decisions to conduct a protective stoma. 6 patients had a CAL. The patients of the control group (n=77) and the patients who suffered from a CAL (n=6) had no increased concentration of urobilinogen and pancreas elastase in the DF. The concentration of bile acid in the DF of the patients who suffered from a CAL differed from those of the control group on the 4th POD (p=0.055).The concentration of bilirubin in the DF of the patients who suffered from a CAL significantly differed from those of the control group on the 1st POD (p=0.031) and on the 3rd POD (p=0.041).

**Conclusion:**

Bilirubin and bile acid in the DF may function as a predictive marker for a CAL.

### Sharp-ended, rootless hair fragments can be found in pilonidal sinus cavities

(Abstract ID: 158)

F. D. Bosche^1^, D. Doll^2^

^1^*Universitätsklinikum Münster*

^2^*St. Marienhospital, Vechta*

**Background:**

Hair has been accused as the causative agent of pilonidal sinus disease (PSD). A great amount of hair can be found in pilonidal sinus cavities. Astonishingly, macroscopic and microscopic examinations of hair found inside pilonidal sinus cavities have been scarce. The purpose of this study was to examine the morphological aspects of the hair found in PSD in order to determine the origin of the hair.

**Materials and methods:**

Occipital, lumbar and intergluteal hair was harvested from 20 PSD-Patients and 20 volunteer matched pair patients. Hair from inside pilonidal sinus cavities was collected intraoperatively from the 20 Pilonidal sinus patients, differentiating between the hair sticking inside the pores and the hair found deeply inside the cavities. Numbers and lengths were recorded and intra and intergroup variations of hair length were characterized using analysis of variance. The hair was examined macroscopically and microscopically using light and scanning electron microscopy with gold an carbon dust coating techniques, thus reaching an enlargement of 1000 times.

**Results:**

The analysis of 624 pilonidal sinus nest hairs found in the 20 sinus cavities reveales rootlessness in 74%. Body hair shows roots on one end. The hair found inside the cavities was significantly shorter than the hair from the other examined body regions (length 0,9±0,7 cm p < 0,0001). Furthermore the hair found inside the cavities was significantly shorter than the hair protruding from pores (p < 0,0001). Microscopic examination shows razor sharp hair ends inside the sinus cavitites. Comparing electron microscopy, these spikey hair end resemble cur hair ends. Overall, the pilonidal hair nest contains between 1 and over 400 hair fragments.

**Conclusion:**

Short hair fragments were found within pilonidal sinus cavities. Moprhologically, these fragments resemble short cut hair rather than intact body hair. These fragments seem so be more capable of entering a pilonidal sinus cavity than longer body hair. The source of these hair fragments has to be eliminated when aiming to prevent pilonidal sinus disease.

**Picture:**
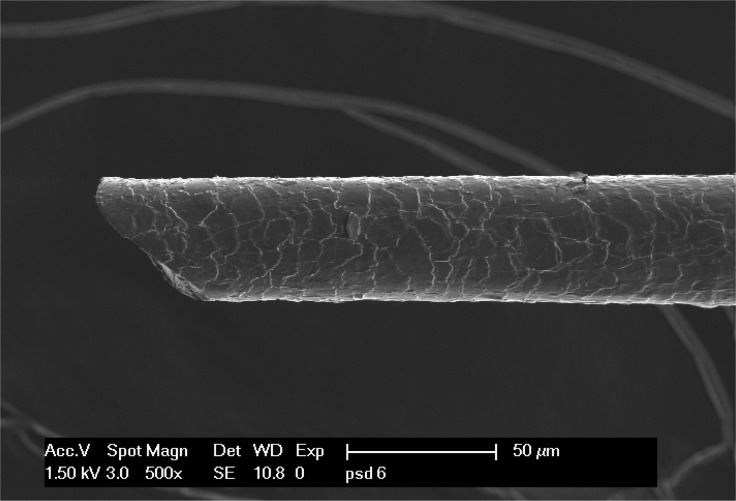
Hair found inside pilonidal sinus cavity (scanning electron microscopy, carbon dust coated)

### Treatment with somatostatin analogs induces differentially expressed let-7c-5p and mir-3137 in gastroenteropancreatic neuroendocrine tumors

(Abstract ID: 166)

F. Bösch^1^, A. Bazhin^1^, S. Heublein^2^, K. Brüwer^1^, T. Knösel^3^, M. Guba^1^, C. Auernhammer^1^, C. Spitzweg^1^, J. Werner^1^, M. K. Angele^1^

^1^*Uniklinik München*

^2^*Universitätsklinik Heidelberg*

^3^*Klinikum der LMU München*

**Background:**

Distant metastases frequently occur in gastroenteropancreatic neuroendocrine tumors. If hepatic surgery is not feasible, patients are treated with somatostatin analogs. However, the underlying mechanisms of action of this treatment remain to be defined. The aim of the present study was to analyze the micro-RNA expression profile intra-individually before and after the treatment with somatostatin analogs.

**Materials and methods:**

Tumor specimens of all included patients (n=8) before and after the onset of a therapy with somatostatin analogs were analyzed and a micro-RNA expression profile (754 micro-RNAs) of each probe was generated. This analysis in an intra-individual setting was selected to avoid bias from inter-individual differences. The micro-RNA expression profiles were validated by qPCR. Patients with any other systemic treatment were excluded from the present study.

**Results:**

Eight patients were included in the present study of which all had neuroendocrine tumors of the small intestine with diffuse hepatic metastases. Grouped analyses revealed that 15 micro-RNAs were differentially expressed (3 up- and 12 downregulated) after the exposure to somatostatin analogs. Additionally, let-7c-5p and mir-3137 are concordantly regulated in the intra-individual analysis.

**Conclusion:**

This is the first study analyzing the individual micro-RNA expression profile before and after a therapy with somatostatin analogs. Data from this study reveal that somatostatin analogs may in part exert their beneficial effects through an alteration in the micro-RNA expression profile.

### Inhibition of SLC7A11 enhances radiosensitivity in p53 null esophageal adenocarcinoma cells

(Abstract ID: 188)

A.-K. Eichelmann^1^, K. Chiam^2^, N. Clemons^3^, D. Liu^3^, SL. Due^2^, I. Bastian^2^, R. Hummel^4^, D. Watson^2^, D. Hussey^2^

^1^*Universitätsklinikum Münster*

^2^*Flinders Medical Centre, Adelaide*

^3^*Peter MacCallum Cancer Centre, Melbourne*

^4^*Uniklinik Lübeck*

**Background:**

Resistance to drug and radiation treatment limits current therapies for oesophageal adenocarcinoma (EAC). The TP53 gene is mutated in 80% of EACs, and given the central role of p53 in controling cellular response to therapy, we study the impact of mutant p53 on EAC response to cytotoxic agents. We previously demonstrated that knockout of mutant p53 can result in increased radiation and drug resistance in EAC cells. In another study we reported that the expression of SLC7A11, a component of the redox maintenance system xC-, is suppressed by mutant p53 and this increases susceptibility to oxidative damage. In this current study we sought to investigate the role of SLC7A11 in mediating the increased radiation resistance seen in mutant p53 knockout vs mutant p53 carrying EAC cells.

**Materials and methods:**

We used the JH-EsoAd1 cell line, which has a missense c797G>A mutation in TP53 resulting in an amino acid change of G266E. The IARC transactivation classification for this mutation is “non-functional”. Three CRISPR-mediated TP53 knockout (KO) JH-EsoAd1 clones, and three control parental lines retaining endogenous mut-p53 (parental polyclonal, parental clonal and Cas9 only) were treated. Radiation sensitivity (2Gy) was determined by a clonogenic survival assay. Baseline SLC7A11 expression in parental lines as well as the clones was determined by Western Blot. For transfection experiments, cells were transfected with SLC7A11 siRNA and knockdown of SLC7A11 was confirmed by Western Blot.

**Results:**

Western Blot results confirmed higher expression of SLC7A11 in p53 null cells compared to the Parental cells. SLC7A11 knockdown in the p53 KO cells resulted in 75.2% reduced SLC7A11 expression. Following inhibition of SLC7A11, p53 KO cells showed increased sensitivity to irradiation (2 Gy SF: 46% (SLC7A11) vs. 73% (non-binding siRNA); p=0.0239), while this effect was not apparent in the Parental cells (52% (SLC7A11) vs. 45% (non-binding siRNA); p=0.2).

**Conclusion:**

The current data show that inhibition of SLC7A11 can restore radiosensitivity in p53 null cells, most likely via enhanced sensitivity to oxidative stress. Further studies are required to identify the exact mechanisms underlying the increased sensitivity to ionising radiation following SLC7A11 knockdown in order to develop new therapeutic opportunities to target this amino acid transporter for cancer treatment. Furthermore, the results provide promising starting points for a better understanding of the important role of SLC7A11 in cancer metabolism and redox balance and the influence of p53 on these processes.

**Picture:**
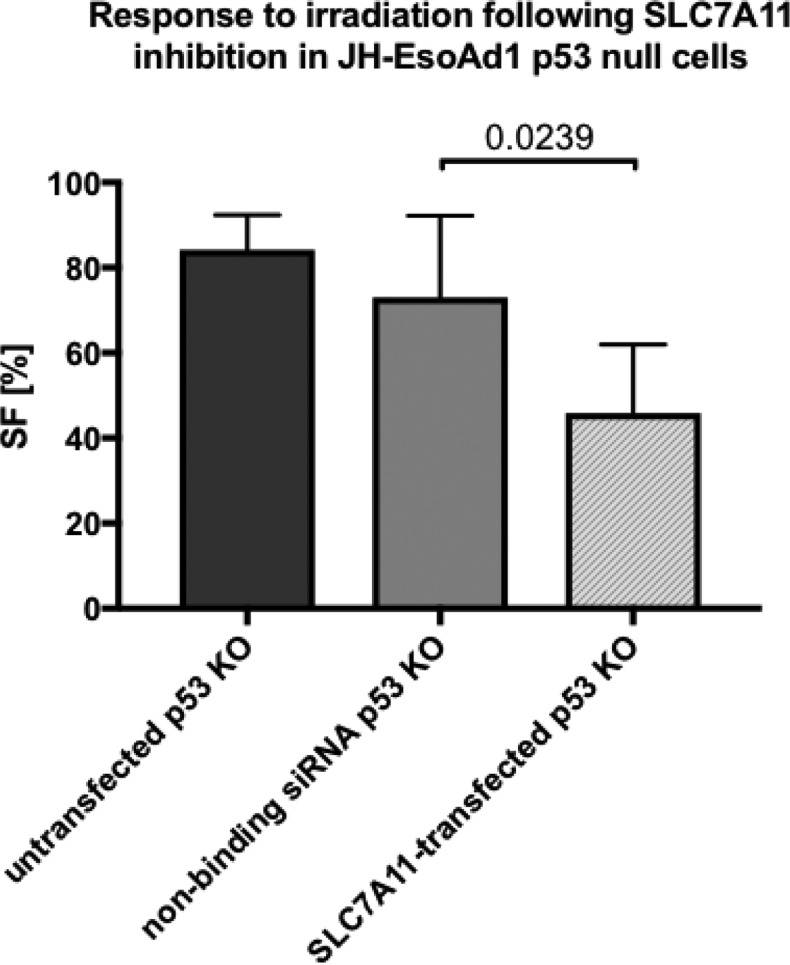


### A new regulatory mechanism involved in ageing

(Abstract ID: 230)

L. Feiner^1^, C. Rubie^1^, M. Glanemann^1^

^1^*Universitätsklinikum des Saarlandes, Homburg (Saar)*

**Background:**

The mechanistic target of rapamycin (mTOR) is known as a key modulator of ageing and age-related disease. The aim of the present study was to discover a potential new regulatory mechanism involved in mTOR related ageing. As microRNAs (miRNAs) are naturally occurring non-coding regulatory molecules and key control elements of crucial regulatory pathways, we aimed to identify a specific miRNA regulating mTOR in an age-dependant mode.

**Materials and methods:**

MTOR and miRNA expression were studied in young (average age 22, n = 40) and old blood donors (average age 78, n = 40) applying qRT PCR and ELISA. Various computer software programmes were employed to identify a specific miRNA that potentially interacts with mTOR. Functional implications of miRNAs with the 3’UTR of mTOR were analyzed by a Luciferase assay system. Accordingly, cell line HELA K was transfected with miRNA mimics and gene and protein expression of mTOR was monitored using qRT PCR and Western blot analysis.

**Results:**

MTOR protein expression was significantly down-regulated in an old cohorte of blood donors with respect to a young cohorte (P < 0.05). Hence, a specific miRNA, miR-496, was identified by target prediction programmes to potentially interact with mTOR and subsequently shown to be significantly up-regulated in the old cohorte with respect to the young cohorte (P < 0.05). Accordingly, miR-496 was demonstrated to functionally interact with mTOR. Thus, addition of miR-496 led to significant down-regulation of luciferase activity (P < 0.05) and mTOR expression after transfection of HELA K cells with the respective miRNA mimics (P < 0.05). Further, functional effects on mTOR downstream activity related to miR-496 were demonstrated.

**Conclusion:**

We have demonstrated an age-related aberrant and inverse expression pattern of mTOR and miR-496 in an old cohorte of blood donors with respect to a young cohort. Moreover, this miRNA was shown to regulate mTOR expression in vitro and to affect mTOR downstream activity. Together, these results strongly indicate that regulation of mTOR expression by miR-496 is a new regulatory mechanism involved in ageing.

Origin: Rubie C. et al.: microRNA-496 - A new, potentially aging-relevant regulator of mTOR. Cell Cycle 2016; 15:1108-1116.

### Diabetes mellitus type 2 and obesity are potentially regulated by the mTOR pathway under the control of miR-496

(Abstract ID: 231)

L.-K. Feiner^1^, C. Rubie^1^, J. Zimmer^1^, M. Glanemann^1^

^1^*Universitätsklinikum des Saarlandes, Homburg (Saar)*

**Background:**

Mechanistic target of rapamycin (mTOR) regulates lipid and glucose metabolism thus playing a key role in metabolic diseases like type 2 diabetes mellitus (T2DM). Recently, we demonstrated a functional interaction of miRNA-496 (miR-496) with mTOR and its impact on the regulation of human ageing. As T2DM is most prevalent in older adults, we hypothesized that miR-496 may also have an impact on mTOR regulation in T2DM.

**Materials and methods:**

Based on real time PCR and enzyme-linked immunosorbent assay (ELISA) mTOR gene and protein expression as well as miR-496 expression was monitored in Peripheral Blood Mononuclear Cells (PBMC) from T2DM patients (median age: 71) and healthy age- and BMI matched controls (median age: 69). Further the mTOR protein- and miR-496 expression was determined in T2DM patients with different degrees of obesity.

**Results:**

Our data indicate miR-496 involvement in the regulation of T2DM through the control of mTOR. We further demonstrate that this interaction may be dependent on the patient’s body mass index with significantly elevated mTOR activity reflecting the progression of the underlying obesity disorder.

**Conclusion:**

Monitoring mTOR expression may be considered as a marker for T2DM development and the progression of obesity. As mTOR up-regulation caused by obesity maintains circulating fatty acids within the physiological range, the BMI-dependent mTOR up-regulation may be considered as a compensatory mechanism to protect the body from metabolic harm.

Origin: Rubie C. et al.: mTOR and microRNA 496 are associated with type 2 diabetes mellitus and obesity in eldery people. ANM 2019.

### A prospective bicentric study to evaluate surgical outcome and lung function changes after laser–assisted pulmonary metastasectomy

(Abstract ID: 249)

M. Hassan^1^, T. Graeter^2^, B. Haager^1^, I. Dietrich^1^, L. J. Kemna^1^, B. Passlick^1^, S. Schmid^1^

^1^*Uniklinik Freiburg, Freiburg im Breisgau*

^2^*Lungenklinik Löwenstein*

**Background:**

The surgical resection of pulmonary metastases (PM) is associated with a survival benefit in selected patients. The use of laser-assisted surgery (LAS) for PM has been shown in a variety of retrospective studies to facilitate the complete resection, especially for higher number of metastases, while preserving a maximum of healthy parenchyma. This is the first prospective study to evaluate perioperative surgical, oncologic and clinical parameters including the changes of lung function after LAS.

**Materials and methods:**

This is an interim-analysis of a prospective, bicentric, single-arm trial. So far we analyzed 78 operations in which PM was carried out in curative intent. A 1.320 nm diode-pumped Nd: YAG-Laser was used for resection of the metastases. Surgical and clinical data were collected using a standardized form and postoperative lung function changes after 3 and 6 months were assessed using whole body plethysmography and diffusion capacity for carbon monoxide (DLCO).

**Results:**

Median operative time was 129 minutes (range, 55-334 minutes) and a median of 3 metastases were resected per operation (range 1-23). The median duration of postoperative air leak was 1 day (range 0-11 days), and median length of hospital stay was 8 days (range, 4-15 days). LAS associated, postoperative minor complications were observed in 7 (9%) cases and there were no mortalities. The analysis of perioperative lung function showed that mean FEV1 3 months after surgery was reduced by 13% (p<0.0001) and DLCO by 14% (p=0.001) respectively. There was no relevant regeneration of lung function at 6 months. Decline of DLCO correlated with the number of resected metastases (r=0.48, p=0.01). All metastases were radiologically measured for distance to pleural surface and size. Adding up diameter and distance to pleural surface there was a positive correlation to the changes of the residual volume at 3 months (r=0.52, p=0.04).

**Conclusion:**

We present excellent results after LAS even in high numbers of metastases with short duration of postoperative air leak and little morbidity. Number, size and depth of the metastases affect lung function changes after resection, particularly diffusion capacity and residual volume.

**Picture:**
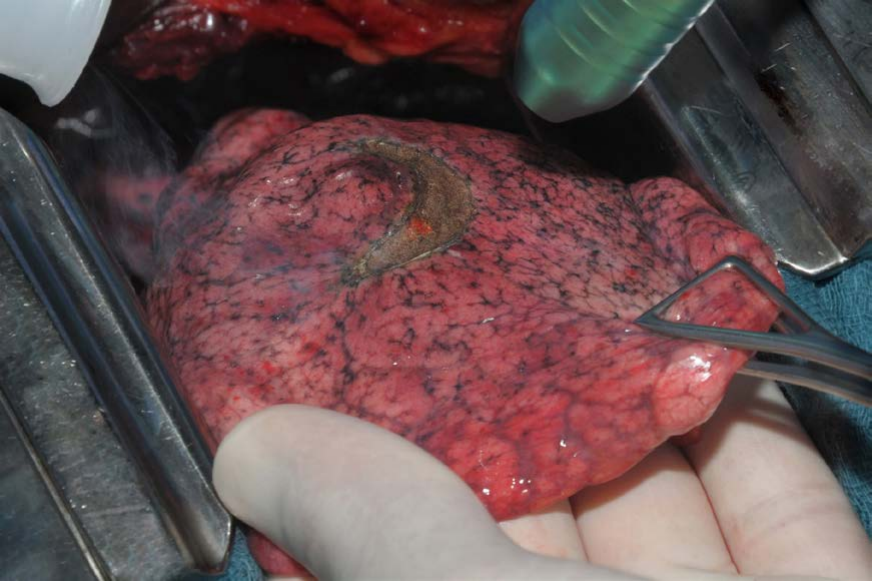
laser–assisted surgery for pulmonary metastases

### Differences in gene expression in colorectal cancer with hepatic vs. peritoneal metastases

(Abstract ID: 254)

E. Pretzsch^1^, F. Bösch^1^, C. Lampert^1^, A. Bazhin^1^, J. Werner^1^, M. K. Angele^1^

^1^*Uniklinik München*

**Background:**

Introduction. Molecular and biological differences in primary tumors of metastasized colorectal cancer (CRC) have been shown to be associated with metastatic route. In this respect, increased expression of tumor stem cell markers and differences in miRNA expression profiles (i.e. miRNA 31 affecting EMT via c-Met) are present in CRC with hepatic vs. peritoneal metastases. Aim of the present study was to further elucidate the underlying mechanisms for the above observations by investigating gene expression patterns of primary CRC with liver vs. peritoneal metastases.

**Materials and methods:**

Material and Methods. CRC with (A) hepatic metastases (n=10), (B) peritoneal metastases (n=10) and (C) locally advanced CRC without metastases for 5 years (n=10) was analyzed. RNA of the primary tumors was isolated by microdissection from the pathological specimens. A NanoString analysis (nCounter® PanCancer Progression Panel) of 770 genes was performed. Gene expression was analyzed by conducting univariate analysis using the Chi-square test; p-values less than 0.05 were considered significant.

**Results:**

Results. Genes are significantly differentially expressed depending on the metastatic pattern. Patients of group A had 26 differentially expressed genes, mainly associated with epithelial-mesenchymal transition (EMT) and angiogenesis, compared to group C. Patients of group B had 18 differentially expressed genes compared to group C. In contrast to group A, EMT associated genes were downregulated in group B. Moreover, angiogenesis associated genes did not play a pivotal role in group B.

**Conclusion:**

Discussion. For the first time it was shown that CRC leading to hepatic or peritoneal metastases exerts a difference in gene expression. An upregulation of genes associated with EMT or angiogenesis was found in CRC with hematogenous spread to the liver. However, a downregulation of EMT associated genes was seen in CRC with peritoneal carcinomatosis. These findings suggest that differences in the gene signature of the primary tumor trigger and direct the metastatic route of CRC.

### The effects of selective androgen receptor and selective estrogen receptor modulators ostarine and raloxifene on skeletal muscle in an osteopenic rat model

(Abstract ID: 292)

L. F. Noisser^1^, M. Komrakova^1^, K. O. Böker^1^, R. Wigger^2^, D. Hoffmann^1^, AF. Schilling^1^, W. Lehmann^1^, S. Sehmisch^1^

^1^*Universitätsmedizin Göttingen*

^2^*Georg-August-Universität Göttingen*

**Background:**

The prevalence of age-related diseases like osteoporosis and sarcopenia is increasing. While bone loss is commonly treated, sarcopenia is often neglected. However targeting sarcopenia is especially important after an osteoporotic fracture as bone healing causes further muscle atrophy through inactivity.

Low sex hormone concentrations are among the key reasons for sarcopenia and muscle weakness. Hormone replacement therapy (HRT) with estrogen or testosterone has been shown to increase muscle mass and function. HRT however can lead to side effects especially on the cardiovascular function. Selective estrogen and androgen receptor modulators (SERMs and SARMs) target similar hormone receptors and are thought to have fewer side effects than endogenous hormone substitution.

This study is focused on the effects of the SARM ostarine (OS) and/or the SERM raloxifene (RL) on skeletal muscle in an osteopenic rat model undergoing bone healing period.

**Materials and methods:**

Three-month-old female Sprague-Dawley rats (n=60) were ovariectomized (OVX) to mimic postmenopausal conditions, while 15 non-ovariectomized rats (NON-OVX) were used as controls.

OVX rats were divided into 4 groups, of 15 rats. Group 1 was left untreated (OVX). Group 2 received OS (OVX+OS). Group 3 was treated with RL (OVX+RL). Group 4 underwent combined treatment with OS and RL (OVX+OS+RL). The average daily doses were 0.6 mg/kg body weight (BW) for OS and 11 mg/kg BW for RL. Eight weeks following OVX, all rats underwent osteotomy of both tibia metaphysis with plate osteosynthesis. Treatments lasted up to 13 weeks after OVX and ended on extraction and weighing of the M. gastrocnemius, M. soleus, M. longissimus, and the uterus. The cross-sectional areas of glycolytic, intermediate, and oxidative fibers in muscles were measured. Cell-nuclei and capillary density were quantified. In serum creatine kinase activity was assessed.

**Results:**

At experiment onset, BW did not differ between groups, but had increased significantly at the end in the OVX+OS and OVX groups compared to all other groups. BW of OS rats was higher than that of OVX rats. The changes in muscle weight (MW) were similar to those in BW. MW was the highest in OS group. Compared to the OVX and OVX+RL groups, the NON-OVX, OVX+OS, and OVX+OS+RL groups had a significantly higher uterus weight.

Fiber size (FS) was larger in OVX and OVX+OS groups than in any other. The FS in OVX+RL and OVX+OS+RL groups was similar to that in the NON-OVX group.

Nucleus density was higher in the OVX+OS+RL group than in the NON-OVX group in the M. gastrocnemius. No differences between the groups were observed in other muscles. Capillary density increased in OVX+OS group in M. soleus and M. longissimus. In M. gastrocnemius, the NON-OVX group had the lowest capillary density.

**Conclusion:**

The OS treatment alone increased BW, MW and capillarization, whereas FS remained at the level of OVX rats. Enhanced uterus weight after OS treatment alone and combined with RL suggests an androgen-like activity of OS in the uterus. This may be considered as unfavorable side effect. Rats under RL and OS+RL treatments demonstrated muscle structures similar to NON-OVX rats. RL treatment alone did not change uterus weight. Thus, RL treatment appears to be more favorable than OS or OS+RL for treating muscle tissue in estrogen-deficient individuals. Considering its current application in osteoporosis therapy, RL could be an alternative treatment for other musculoskeletal diseases, including post-fracture muscle loss.

### High Resolution Respirometry (HRR) of the smooth muscle in the intestine

(Abstract ID: 313)

F. Speichinger^1^, C. Kamphues^1^, A. Gratl^1^, J. Frese^1^, A. Greiner^1^, M. Kreis^1^

^1^*Charité - Unversitätsmedizin Berlin CBF*

**Background:**

High Resolution Respiratory has been shown as an effective tool to measure the mitochondrial respiration in striated muscle, as well as the measurement of citrate synthase activity (CSA) has been shown to give information of the amount of mitochondria in a tissue.

In patients with peripheral arterial disease it has been shown that the function and the amount of mitochondria depends on the blood supply measured by the HRR.

So far, there is no knowledge about the High Resolution Respiratory in the smooth muscle of the intestine.The investigation of the mitochondria in the smooth muscle of the intestine could be of great interest e.g. because the occurrence of anastomotic leakage is still a relevant complication in the colorectal surgery with poorly understood mechanism.

**Materials and methods:**

10 patients were included. The HRR and measurement of the CSA was performed with all samples. Some of them have been analysed with the TEM.

**Results:**

Through High Resolution Respirometry performed by using an Oxygraph-2k we were able to evaluate the mitochondrial respiration of the smooth muscle in the intestine. According to our findings of striated muscle in patients with peripheral arterial disease we observed similar results on a lower level, as expected due to the lower content of mitochondria in the smooth muscle. After correction for the CSA we could see similar ratios and proved that the HRR is transferable to smooth muscle tissue. Further evidence for the portability of smooth muscle we examine the Tunica muscularis of the intestine by transmission electron microscopy (TEM) and compared it with the results of straited muscle with similar results.

**Conclusion:**

By High Resolution Respirometry using an Oxygraph-2k we were able to measure the activity and amount of mitochondria of the smooth muscle in the intestine.

Thereby we established a new tool for further questions such as the function of mitochondria in reduced blood circulations in the intestine associated with anastomotic leakage.

### Clinical relevance of circulating tumor cells in esophageal cancer – Comparison of two immunological enrichment methods

(Abstract ID: 321)

A. Wöstemeier^1^, K. Harms-Effenberger^1^, S. Riethdorf^1^, F. G. Uzunoglu^1^, K. Pantel^1^, M. Bockhorn^1^, M. Reeh^1^, J. R. Izbicki^1^

^1^*Universitätsklinikum Hamburg Eppendorf*

**Background:**

Current modalities to predict tumor recurrence and survival in esophageal cancer are insufficient. Locoregional and distant relapse is even common in lymph node-negative patients and more precise staging methods are therefore needed. So far, only the CellSearch system was used to detect circulating tumor cells (CTC) in esophageal cancer patients. Studies analyzing different CTC detection assays using combined enrichment techniques to potentially increase the sensitivity are missing.

**Materials and methods:**

In this single-centre, prospective study, peripheral blood samples from 100 esophageal cancer patients were obtained preoperatively and analyzed for the presence of CTC by two distinct methods. A total of 90 cases have been evaluated for the presence of CTCs by MACS enrichment (anti-cytokeratin/ anti-Ep-Cam) with subsequent immunocytochemical staining and in parallel by the CellSearch method. Data were correlated with clinicopathological parameters and patient outcomes.

**Results:**

CTCs were detected in 25.6% (23/90) of patients by MACS enrichment/Ariol (0-150 CTCs/7.5ml) and in 17.8% (16/90)of patients (0-56 CTCs/7.5ml) using the CellSearch system. CTCdetection with the CellSearch system correlated significantly with the pT stage and M stage. However, no significant correlation has been found with histopathological parameters and CTC detection with the combined cytokeratin/EpCAM enrichment. The mean follow-up time was 38 months. Within this time, 65/100patients relapsed and died, respectively. End points were progression-free (PFS) and overall survival (OS). Presence of CTCs correlated with significantly shorter OS and PFS in univariate and multivariate analysis using MACS enrichment or the CellSearch method.

**Conclusion:**

CTC detection by MACS enrichment/ Ariol correlates with a shorter progression-free and overall survival, using a cut of value of >= 2 CTCs, whereas patients with >1 CTC or <= 1CTC did notshow significant differences in PFS and OS. However, the CellSearch system is a strong independent prognosticator for overall and progression-free survival, regardless of the cut offvalue and thus remains the gold standard for CTC detection.

### Establishment of a microbiome analysis pipeline from feces, scrapings and fresh-frozen mucosa for in-depth analysis of biobank tissue samples

(Abstract ID: 343)

U. Wirth^1^, D. Garzetti^2^, M. Koeppel^2^, D. Ring^2^, J. Werner^1^, B. Stecher^2^, M. Rentsch^3^, T. Schiergens^1^

^1^*Uniklinik München*

^2^*Max von Pettenkoferinstitut für Hygiene und Medizinische Mikrobiologie der LMU München*

^3^*Klinik für AVT, München*

**Background:**

The human microbiota has recently become subject of multiple investigations because of its role in development or maintenance of different physiological and pathological states of the human body. Due to its interaction with tissues, cells and the immune system on external and internal body surfaces, it has important influence on prevention as well as development of inflammatory, benign and malignant tumorous diseases. With the development of biobanking of human tissue samples, such as biopsies or tissue samples of surgical specimens, a multitude of normal and pathological tissue samples has been stored and is available for up-to-date research, especially in the fields of personalized medicine and pathogenesis of human diseases.

The aim of our work was to establish and validate a microbiome analysis pipeline for different kinds of snap frozen biobank samples including feces, mucosa scrapings (containing the intestinal mucin layer) and mucosa samples.

**Materials and methods:**

Samples (feces, mucosal scrapings and mucosa) from normal tissues of transverse colon from surgical specimens were collected during surgery. The samples were stored snap-frozen in the biobank at our surgical department. Microbiome analysis of the fecal samples was performed according to protocols normally used for human and mouse intestinal samples: after DNA extraction and amplification using specific primers targeting the V3-4 variable regions of the bacterial 16S rRNA gene, amplicons were sequenced on the Illumina MiSeq v.3 platform. Additionally, the routine protocol for microbiome analysis in stool was expanded for the analysis of mucosa scrapings and mucosa samples. For validation of microbiome analysis, number of taxa, α- and ß-diversity metrics and differential abundance analysis were performed.

**Results:**

Microbiome analysis from fecal samples and scrapings were successfully established according to the already existing protocols. DNA extraction from mucosa samples was optimized to yield enough high-quality DNA and amplicons for successful sequencing. Validation of this pipeline was performed based on different population measures like absolute number of taxa, α - and ß-diversity and differential abundance analysis.

**Conclusion:**

After successful establishment and validation of microbiome analysis from snap frozen mucosa samples, it seems feasible to perform microbiome analysis on samples already stored in a biobank. The entire biobank collection of tissues obtained from surgical specimens resected during inflammatory (like inflammatory bowel disease) and mostly malignant diseases (all kind of gastrointestinal and other cancers), is therefore potentially available for microbiome analysis. Together with the clinical documentation, this data will be valuable to correlate disease states with microbiota profiles. The interaction between human microbiota and the pathogenesis and progression of different diseases, as well as the microbiota’s impact on development of perioperative infectious complications could be elucidated in future projects using a comprehensive range of phenotypes of a surgical biorepository.

### Reduction of perioperative fasting periods and improved nutrient supply by intensified nutritional support of patients undergoing gastrointestinal tract surgery

(Abstract ID: 357)

T. Wuensch^1^, J. Quint^1^, V. Müller^1^, M. Biebl^1^, J. Pratschke^1^, F. Aigner^1^

^1^*Charité - Universitätsmedizin Berlin CVK*

**Background:**

Prolonged perioperative fasting periods are associated with delayed recovery after surgery. Clinical guidelines (e.g. ESPEN, 2017) emphasize the benefits and feasibility of preoperative fasting of solid food for only six hours and clear fluids for two hours before induction of anaesthesia. Furthermore, oral feeding should not be interrupted by surgery and has been shown to be safe even in patients undergoing surgery for gastrointestinal (GI) tract malignancies. The aim of our study was to assess, whether these fasting recommendations are achieved in clinical practice in patients undergoing GI tract surgery. In addition, this study seeks to identify potential nutritional support improvement strategies during the clinical care process.

**Materials and methods:**

Patients scheduled for elective surgery of the upper (n=23) or lower (n=27) GI tract participated in a prospective observational study. Patients’ charateristics and nutritional status (assessed by Mini Nutritional Assessment, MNA and Nutritional Risk Screening, NRS) were recorded and blood samples were drawn on the day of admission and on postoperative day (POD) four. Nutrient intake and timing was assessed by nutrition diaries during hospitalization. Intensified nutritional support included preoperative nutrition counseling and daily nutrition support during hospitalization by a dietitian.

**Results:**

Within our patient cohort, the prevalence of "risk for malnutrition" was 61.2% or 38% when assessed with the NRS or MNA score, respectively. The MNA also graded 6.3% of the patients as "malnourished" at the time of admission.

Preoperative fasting periods of solid food ranged between 9.4-89.3 h (median: 14.4 h) in patients undergoing upper GI tract surgery and 10.8-40.3 h (median: 22.7 h) in patients undergoing lower GI tract surgery. Postoperatively, fasting periods were significantly different between patients undergoing upper GI tract surgery (median: 92.7 h, range 21.9-138.4 h) as compared to patients undergoing lower GI tract surgery (median: 23.2 h, range 10.0-78.8 h). The most significant reductions of fasting periods by intensified nutritional support was achieved preoperatively in patients undergoing lower GI tract surgery (median: 13.7 h, range 10.3-30.8 h) and postoperatively in patients undergoing upper GI tract surgery (median: 41.3 h, range 38.3-59.3 h). By the intensified nutritional support, the total energy deficit accumulating between the day of admission till POD4 was reduced by 27%.

Prolonged preoperative fasting was reflected in disturbed amino acid plasma profiles with significantly elevated branched-chain amino acid levels and reduced non-essential amino acids, like glutamic acid. Postoperatively, the fasting periods correlated significantly positive with C-reactive protein and 3-methylhistidine plasma levels, the latter being a marker of muscle protein degradation.

**Conclusion:**

Significant reductions of perioperative fasting periods and an improve daily energy intake were achieved by the intensified nutritional support of surgical patients. Despite the high levels of awareness of the medical staff for the importance of sufficient feeding of the patients, there is a discrepancy between guideline-adhering short perioperative fasting periods and the actual achieved times in the individuals‘ clinical treatment process.

### Hepatic CYP1A2 activity in liver tumors and the implications for preoperative volume-function analysis

(Abstract ID: 395)

T. Wuensch^1^, N. Heucke^1^, J. Wizenty^1^, B. Sinn^2^, R. Arsenic^2^, M. Jara^1^, M. Kaffarnik^3^, J. Pratschke^1^, M. Stockmann^1^

^1^*Charité - Universitätsmedizin Berlin CVK*

^2^*Charité - Universitätsmedizin Berlin CCM*

^3^*Charité - Campus Virchow Klinikum, Berlin*

**Background:**

One strategy for preoperative risk assessemnt in liver surgery includes the combined assessment of the dynamic liver function by the 13C-methacetin maximal liver function capacity (LiMAx) test, the volumetric analysis of the liver and calculation of future liver remnant function. However, this so-called volume-function analysis assumes that the remaining CYP1A2 activity (the enzyme system specifically addressed by the LiMAx test) in any tumor lesion is zero. This study aims to assess the remaining CYP1A2 activities in different hepatic tumor lesions and its consequences for the volume-function analysis.

**Materials and methods:**

The activity of CYP1A2 was determined in human liver tissue samples collected after surgical resection of hepatocellular adenomas (HCA, n=9), hepatocellular carcinomas (HCC, n=25) or colorectal liver metastases (CRLM, n=5). Paired tissue samples were collected, one from the lesion and one from adjacent non-tumor liver tissue. CYP1A2 abundance was assessed by immunofluorecence staining of paraffine-embedded tissue sections. In all patients a LiMAx test was performed prior to surgery and representative volume-funtcion analysis were conducted.

**Results:**

CYP1A2 activity was significantly higher in non-tumor tissues (44.30 ± 36.94 μU/mg) as compared to HCA (11.23 ± 18.00 μU/mg), HCC (1.19 ± 2.40 μU/mg) or CRLM (0.87 ± 1.63 μU/mg), respectively. The mean percentage decline compared to adjacent non-tumor tissue was 74,7% in HCAs, 97.3% in HCCs and 98.1% in CRLMs, respectively. The CYP1A2 activity differences were also reflected in CYP1A2 protein signals in the assessed hepatic tissues. Volume-function analysis showed a minimal deviation compared to the current standard calculation for HCC (<1% difference) while a difference of 14.3% was observed for HCA.

**Conclusion:**

In conclusion, CYP1A2 activity is virtually absent in HCCs but considerable activity detectable in HCAs. These findings are important for an accurate surgical risk assessment and an improved preoperative volume-function analysis in HCA cases with low LiMAx values.

### High level infrasound exposure reduces the contractility of human cardiac tissues in in-vitro model

(Abstract ID: 410)

R. Chaban^1^, C.-F. Vahl^1^

^1^*Universitätsmedizin Mainz*

**Background:**

Human exposure to infrasound is increasing due to different factors, raising questions regarding its public safety. The aim of this work is to evaluate whether this exposure interferes directly with human cardiac function and hence attributes to any kind of pathological process.

**Materials and methods:**

Human myocardial tissues, obtained from patients undergoing cardiac surgery, were prepared in small muscle samples and then stimulated electrically with a frequency of 75 bpm for a period of almost 120 minutes under sustained perfusion with an oxygenated physiological solution. Two samples were obtained from each patient: one was subjected to infrasound and the other served as a control. The exhibited isometric contraction force (CF) and contraction duration (CD) were measured before and after the treatment. The changes in these values were compared to the control samples in each trial. Three groups of trials were conducted with three different levels of infrasound: 120, 110 and 100 dB SPL. Each group contained 6 trials.

**Results:**

Using linear regression, we found a correlation between increasing infrasound level above 100 dB SPL und decreasing CF (r^2^=0.26; one-sided p= 0.014). The 120 dB SPL group showed a decrease in its CF of almost 20.6% in comparison to its control. The 110 dB SPL showed a decrease of 11.7%. There was no significant change in CF in the 100 dB SPL group. The CD remained unchanged in all groups.

**Conclusion:**

Exposure to high levels of infrasound (120 dB SPL) resulted in a relevant (18%) and statistically significant decrease in CF. These results should be considered when looking at environmental regulations.

**Picture:**
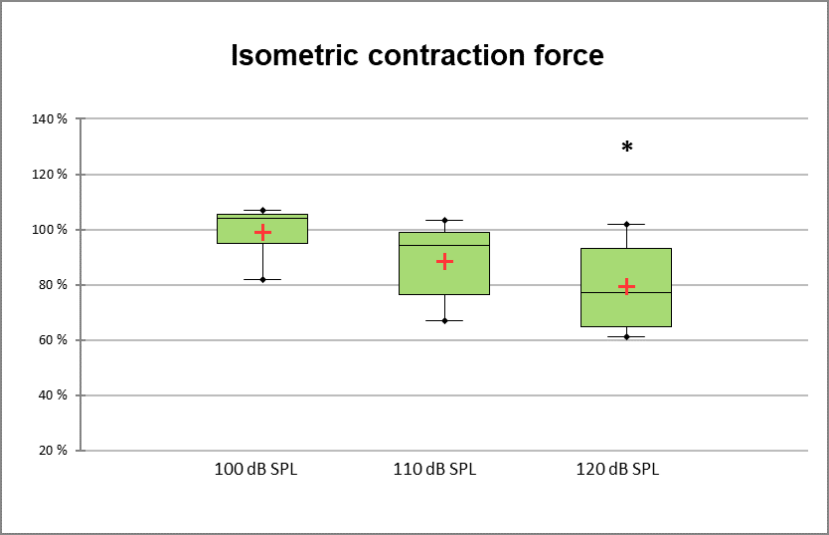


### Expression of the anti-inflammatory protein Annexin A1 correlates with intestinal inflammation in patients with Crohn´s disease

(Abstract ID: 421)

P.-A. Neumann^1^, J. Troger^1^, S. Reischl^1^, E. Miltschitzky^1^, H. Friess^1^, G. O. Ceyhan^1^

^1^*Klinikum Rechts der Isar der TU München*

**Background:**

Annexin A1 (AnxA1) is a potent anti-inflammatory protein that has been described as one of the main effector molecules of glucocorticoids. It suppresses leukocyte activation and transmigration and exhibits anti-inflammatory properties in inflammation models such as endotoxemia, peritonitis and arthritis. Furthermore AnxA1 has been shown to be centrally involved in intestinal wound healing and inflammation. Thus it shows high potential as an immune regulator for inhibition of inflammation and stimulation of wound closure in the gut. Little is known about the endogenous role of AnxA1 in inflammatory bowel disease. Thus we aimed to further characterize the expression of AnxA1 in Crohn´s disease.

**Materials and methods:**

To analyze the endogenous role of AnxA1 in Crohn´s disease, surgical specimen of patients who have had intestinal resections for Crohn´s disease have been analyzed (n=60 patients): For protein analysis and evaluation of AnxA1 expression-patterns immunohistochemistry was performed and corroborated with analysis of AnxA1 RNA expression using realtime PCR (n=20 patients). Furthermore serial immunofluorescence co-stainings of cell markers and AnxA1 were used to determine the main cell source of AnxA1 expression within the tissue. Ultimately AnxA1 expression was correlated to histologic scoring of the specimen and the clinical course of disease of the patients.

**Results:**

Immunohistochemistry (IHC) revealed correlation of AnxA1 expression with increase of intestinal inflammation. Using co-staining of AnxA1 with different cell markers, we identified CD4 positive T cells to highly positive for AnxA1. Corresponding to the results of IHC, RT-PCR showed elevated expression of AnxA1 RNA in samples of inflamed areas compared to non-inflamed tissue which correlated well with the histologic scoring of the tissue.

**Conclusion:**

Within our analysis, the expression pattern of the anti-inflammatory protein AnxA1 correlated well with intestinal inflammation in patients with Crohn´s disease. We will now further characterize the expression of AnxA1 with clinicopathological features of the disease to determine whether the protein might serve to be a valid biomarker for the disease. Due to its anti-inflammatory character and role in regulation of intestinal inflammation AnxA1 might be a promising therapeutic in inflammatory bowel disease.

### Long-term outcome of venous thrombectomy combined with anticoagulation vs. anticoagulation alone for acute inferior vena cava thrombosis

(Abstract ID: 437)

F. Dahi^1^, C. Klaeffling^1^, W. Derwich^1^, D. Gray^1^, G. Jung^1^, M. Keese^2^, T. Schmitz-Rixen^1^

^1^*Universitätsklinikum Frankfurt am Main*

^2^*Universitätsmedizin Mannheim*

**Background:**

Aim of the present restrospective study is to compare the long-term clinical outcomes of surgical (venous thrombectomy and anticoagulation) vs. conservative therapy (anticoagulation) for acute inferior vena cava thrombosis.

**Materials and methods:**

All patients with thrombosis of the inferior vena cava treated conservatively or surgically from January 2005 to December 2017 at University Hospital Frankfurt were included in the study. Long- term outcomes in terms of venous patency and development of post- thrombotic syndrome (PTS) were analyzed.

**Results:**

A total of 48 patients with acute inferior vena cava thrombosis were treated in this period in our hospital. Twenty-three patients (8 female, 15 male; mean age, 40 years; range, 14-72 years) underwent transfemoral venous thrombectomy combined with a temporary arterio-venous fistula and anticoagulation. Four patients underwent additionally balloon angioplasty and stenting of an iliac vein stenosis due to May-Thurner Syndrome.

25 patients (15 female, 10 male; mean age, 48 years; range, 12-75 years) were treated conservatively with oral anticoagulation (coumarine derivatives). In the 6- and 24 months follow-up, venous patency was controlled using duplex ultrasonography. Development of PTS was assessed using the Villalta score. In the 24 months follow- up, the patency rate of the inferior vena cava and iliofemoral veins was 85,7 % in the surgical group, and 72 % in the conservative treatment group (P=0.231). 21,5% of patients in the surgical group developed moderate to severe PTS compared to 33,0% in the conservative group (P=0.715). In the surgical group 5 patients (22%) developed re-thrombosis in the first postoperative week and underwent new surgical procedure. One patient developed pulmonaly embolism. Two patients died because of multi organ insufficiency (mortality rate during first hospital stay: 8%).

**Conclusion:**

Surgical therapy for acute vena cava thrombosis seems to offer better long- term results in terms of venous patency and PTS development compared to anticoagulation alone. Nevertheless, open surgery has a higher incidence of perioperative complications. Furthermore, there is a need for more studies with larger patient populations.

**Picture:**
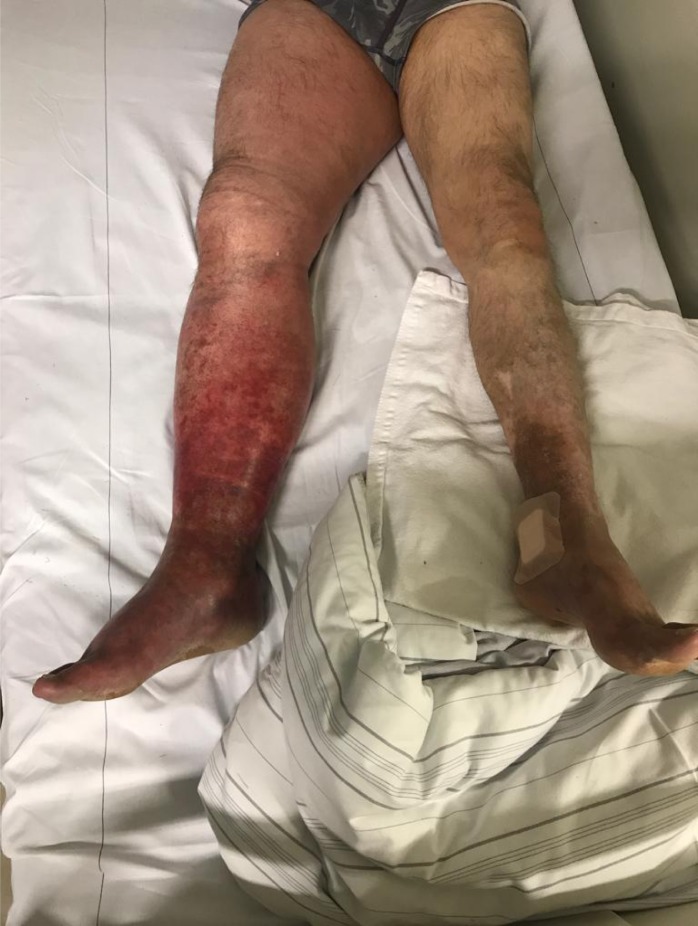


### Prognostic significance of combinding neutrophil to lymphocyte ratio and sICAM sera concentration in patients with colon cancer

(Abstract ID: 483)

M. C. Langheinrich^1^, V. S. Schellerer^1^, E. Naschberger^1^, S. Kersting^1^, R. Grützmann^1^, S. Merkel^1^

^1^*Universitätsklinikum Erlangen*

**Background:**

The systemic inflammatory response (SIR) is a complex system comprising humoral and cellular components and gained growing evidence in the development and progression of cancer. The neutrophil-to-lymphocyte ratio (NLR) is one of the most common hematologic parameters reflecting the systemic inflammation and predict survival in varied type of cancers. Soluble ICAM-1 (sICAM-1) is also an inflammatory associated marker, elevated in different type of cancers. Therefore, in the present study we set out to evaluate the effect and the prognostic relevance of combination NLR and sICAM in a large cohort of patients with colon cancer at the time of initial diagnosis to predict outcome in these patients.

**Materials and methods:**

In this retrospective analysis of prospectively collected blood samples (collected by peripheral venous punction before surgery) 225 patients with previously untreated colon cancer (stage I-IV) were enrolled. Clinical data were obtained from the Erlangen Registry for Colorectal Carcinomas. NLR was measured via the routine blood analysis and receiver operating characteristic (ROC) analysis was used to identify the best cutoff value of NLR. On the basis of our previously published data, sICAM levels were analyzed by commercial available ELISA kit and a cut-off level of 250 ng/ml was identified. Survival curves were determined by the Kaplan-Meier method.

**Results:**

The optimal cut-off value for NLR (NLR=3) was determined using receiver operating characteristic (ROC) curve and the Youden index. Overall, 122 patients presented with NLR>3, 103 patients with NLR<=3; 109 patients with sICAM>=250 ng/ml (sICAM high) and 116 patients with sICAM<250 ng/ml (sICAM low). Patients with NLR>3 and sICAM high (n=66) had a 5-year survival rate of 47%, significantly (p<0,004) worse compared to patients with NLR<=3 and sICAM-1 low (n=60) 80%. Of note, patients with NLR>3 and sICAM high showed trends for inferior DFS compared to patients with NLR<=3 and sICAM low (56% vs 80%), but failed statistical significance.

**Conclusion:**

The combination of NLR and sICAM, both simple and easily obtained markers, is a useful prognostic indicator for OS in patients with colon cancer.

**Picture:**
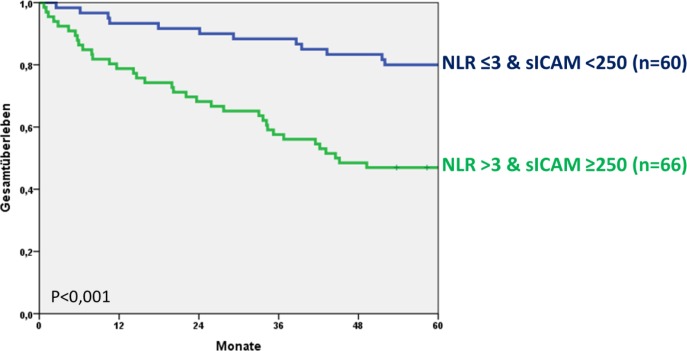
Kaplan-Meier NLR_sICAM OS

### Role of mechanics and tissue architecture in the development of postoperative pancreatic fistulas (POPF)

(Abstract ID: 509)

R. B. Schmuck^1^, A. Cipitria^2^, E. Lippens^1^, D. Wulsten^1^, D. S. Garske^2^, J. Pratschke^1^, I. M. Sauer^1^, G. Duda^1^, M. Bahra^1^

^1^*Charité - Universitätsmedizin Berlin CVK*

^2^*Max Planck Institute of Colloids and Interfaces, Potsdam*

**Background:**

Postoperative pancreatic fistulas (POPF) occur in up to 30% of patients after partial resection of the pancreas. The mechanical properties of the adjacent non-malignant tissue is significantly associated with the development of. Consequences thereof are a prolonged stay in the hospital, which doubles the cost of care after pancreatic surgery, and an increased postoperative morbidity and mortality.

**Materials and methods:**

The aim of this interdisciplinary project was to (i) develop novel techniques to quantify pancreas mechanical properties in an objective manner and (ii) to better understand the tissue composition and structural factors causing alterations in the tissue mechanical properties. Postoperative ex-vivo mechanical testing (bulk unconfined compression testing) was flanked by structural tissue and cell characterization (histology, polarized light microscopy and immunofluorescent staining) and correlated to clinical data and tumor characteristics.

**Results:**

In total, 91 samples of 59 patients were analyzed, including 48 tumor samples and 43 samples of adjacent non malignant tissue. We could clearly show a correlation of the preoperative presence of cholestasis and a reduced rate of POPF. Furthermore, patients who developed POPF after pancreatic surgery showed a more viscoelastic tissue composition of the adjacent non-malignant tissue represented by a shorter stress relaxation half time measurement. A trend was observed for lower fibrous tissue content as well as reduced content of collagen I in adjacent non-malignant tissue in patients developing POPF. We could not detect a difference between the content of fibronectine, hyaluronic acid, collagen III and IV, and patients developing POPF or not.

**Conclusion:**

The risk of POPF seems to be lower in patients presenting with preoperative cholestasis and a more viscoelastic tissue composition of the pancreas remnant. Methods to quantify mechanical properties of the pancreas in an objective manner are needed to establish risk stratification algorithms to identify patients at high risk for POPF.

### Priority Setting Partnership (PSP) Pancreatic Cancer and Colorectal Cancer – bringing together clinicians, patients and carers to discuss research priorities

(Abstract ID: 539)

R. Klotz^1^, C. Doerr-Harim^1^, C. Tjaden^1^, S. Frankenhauser^2^, M. W. Büchler^1^, A. L. Mihaljevic^1^

^1^*University Heidelberg*

^2^*BG Unfallklinik Ludwigshafen*

**Background:**

Colorectal cancer is the third and pancreatic cancer is the fourth leading cause of cancer deaths in Germany. Research projects on cancer are currently initiated by industry or scientists, usually without involving relevant stakeholders (treating physicians, patients, family members, caregivers, nurses, etc.). However, there is a mismatch between available research evidence and the research preferences of consumers, i.e. patients and caregivers. Consequently, involving patients is an upcoming topic in clinical research and health-care politics.

**Materials and methods:**

Priority Setting Partnerships (PSPs) aim to involve patients, caregivers, doctors and other relevant stakeholders as equal partners to find the most urgent unanswered research question for a certain disease. This is achieved by a transparent 7-step process including survey, literature reviews and prioritizations. The PSP Pancreatic cancer and the PSP Colorectal cancer were initiated in Heidelberg in cooperation with the UK-based James Lind Alliance, a non-profit-making initiative established in 2004. The aim is to separately identify and prioritize the top 10 open research questions / uncertainties on (diagnostics and) treatment of the two diseases. The intention of these projects is to stimulate and steer future research in the field of colorectal and pancreatic cancer, by identifying the most important research areas for patients and clinicians.

**Results:**

The first nation-wide survey of the PSP Pancreatic cancer was carried out from August to November 2017 and revealed 566 questions submitted by 140 participants. The gathered uncertainties included questions about medical and surgical therapy, palliative care, naturopathy, nutrition, the impact of psycho-oncology, and more. After removal of duplicates and out of scope questions, uncertainties were checked against current evidence. A second online survey for interim prioritization and the final face-to-face consensus conference will follow.

The PSP Colorectal cancer started in 03/2018 and is founded by the BMBF. Potential stakeholders and partner organization were identified and the online survey is planned for 11-12/2018.

At the congress, the TOP 10 List of the PSP Pancreas and first results of the nationwide online survey of the PSP Colorectal cancer can be presented. The consecutive steps of the ongoing PSPs will explained and discussed.

**Conclusion:**

The identified questions warrant realized patient involvement and initiate patient-relevant research and research funding, thus improving the care of those most affected by pancreatic and colorectal cancer. The presented PSPs establish the transparent, validated JLA method for the first time in Germany and they are the first PSPs on the subject of pancreatic cancer and colorectal cancer world-wide.

### Tissue and blood platinum concentrations after (e)PIPAC and L-HIPEC

(Abstract ID: 546)

U. F. Pabst^1^, P. Bucur^2^, C. R. Demtröder^1^, J. P. Hölzen^1^, S. Roger^3^, N. Tabchouri^2^, M. Ouaissi^2^

^1^*Universitätsklinikum Münster*

^2^*University Tours, Chambray-Les-Tours*

^3^*University Francois Rabelais de Tours*

**Background:**

Pressurized IntraPeritoneal Aerosol Chemotherapy (PIPAC) and its technical modification by the additional use of an electrostatic aerosol precipitation device (ePIPAC) is a new technology to deliver intraperitoneal chemotherapy. It is reported that the ratio between peritoneal and systemic drug concentrations is superior compared to that of liquid hyperthermic intraperitoneal chemotherapy (HIPEC). So far, there is no direct comparative data available supporting such an assumption.

**Materials and methods:**

Three groups of four French landrace pigs each underwent either PIPAC with oxaliplatin 92mg in 150ml dextrose 5% (Group 1), electrostatic aerosol precipitation PIPAC (Group 2) or laparoscopic HIPEC (L-HIPEC) with oxaliplatin 400mg in 4 litres dextrose 5% at 42°C (Group 3). Blood as well as peritoneal tissue concentrations of platinum were determined by Inductively Coupled Plasma-Mass Spectrometry (ICP-MS). To obtain Oxaliplatin concentrations, the measured platinum concentrations must be multiplied with a factor 2.03.

**Results:**

The maximum concentration (mean+/- SD) in all three groups occurred after 50 minutes (Group 1: 0.80±0.07μg/g; Group 2: 0.65±0.08μg/g; Group 3: 0.60±0.09μg/g) with no significant difference between the three groups (Kruskal-Wallis: p>0.794). However, analysing blood concentrations after 10 (Group 1: (0.35±0.01μg/g); Group 2: (0.04±0.05μg/g); Group 3: (0.175±0.02μg/g), 20 (Group 1: (0.06±0.005μg/g); Group 2: (0.06±0.005μg/g); Group 3: (0.36±0.05μg/g) and 30 minutes (Group 1: 0.08±0.005μg/g); Group 2: (0.09±0.01μg/g); Group 3: (0.54±0.02μg/g), significant higher concentrations were found in Group 3 (p<0.05). Overall platinum concentrations in the peritoneum did not differ between the three groups (Kruskal-Wallis: p=0.3452). In all three groups, platinum concentrations found in the parietal peritoneum were significantly higher than in the visceral peritoneum (p=0.0242).

**Table:** 

	10 minutes	20 minutes	30 minutes	40 minutes	50 minutes	60 minutes
Group 1 (PIPAC);	0.035+/-0.01	0.06+/-0.005	0.08+/-0.005	0.52+/-0.04	0.8+/-0.07	0.76+/-0.04
Group 2 (ePIPAC)	0.04+/-0.005	0.06+/-0.003	0.09+/-0.01	0.46+/-0.01	0.65+/-0.08	0.59+/-0.04
Group 3 (HIPEC)	0.175+/-0.02	0.36+/-0.05	0.54+/-0.02	0.54+/-0.08	0.6+/-0.09	0.57+/-0.13

Mean +/- Standard deviation of blood platinum concentrations (microgram/gram)

**Conclusion:**

During PIPAC and ePIPAC, relevant platinum absorption, similar to that observed during L-HIPEC was found. Irrespective of the technique used, the overall platinum concentrations measured in the peritoneum showed also no significant differences among the three groups. No superiority was found for ePIPAC.

**Picture:**
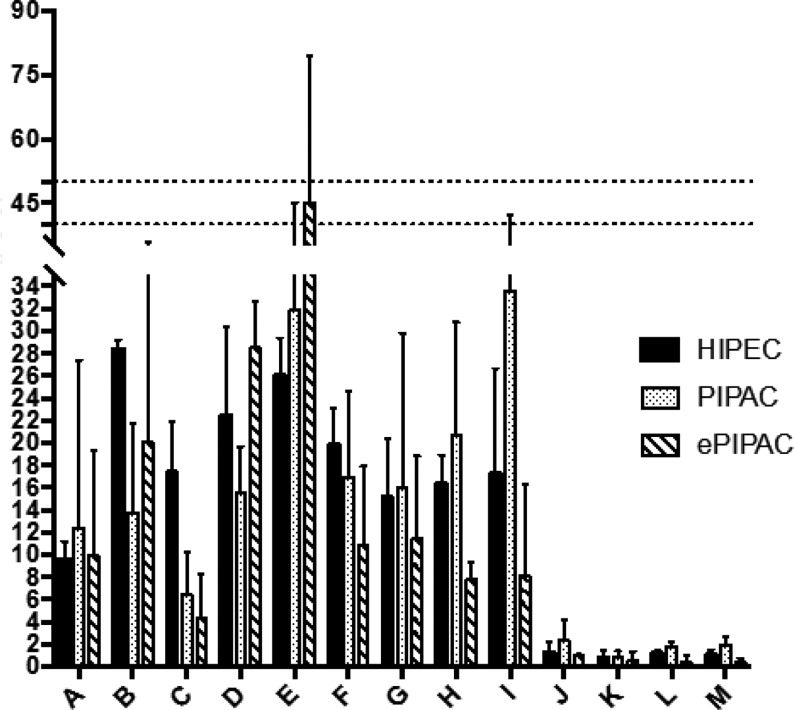
Platinum in the parietal (A-I) and visceral (J-M) peritoneum. x-axis: areas of tissue sampling according to Sugarbaker PCI-score area 0 to12; y-axis: platinum concentration (mikrogram/gram)

### Renal Impairment is associated with reduced outcome after Associating Liver Partition and Portal Vein Ligation for Staged Hepatectomy: Data from the ALPPS Registry

(Abstract ID: 619)

T. Reese^1^, M. H. Fard-Aghaie^1^, G. Makridis^1^, A. Kantas^1^, M. Malagó^2^, E. de Santibañes^3^, P.-A. Clavien^4^, H. Petrowsky^4^, M. Linecker^4^, K. J. Oldhafer^5^

^1^*Asklepios Klinik Barmbek, Hamburg*

^2^*Royal Free Hospital, London*

^3^*Italian Hospital Buenos Aires*

^4^*University Hospital Zurich*

^5^*Asklepios Campus Hamburg*

**Background:**

Impaired postoperative renal function increases morbidity and mortality after liver resection. Preoperative laboratory values such as elevated alanine transaminase and an increased MELD score and comorbidities (cardiovascular disease, diabetes, chronic renal failure and advanced age) have an impact on the incidence of postoperative renal function. However, intraoperative risk factors, such as major hepatectomy and prolonged operative time, are the most important components to have impact on postoperative renal function.

It is unknown whether the incidence and risk factors for impaired renal function after standard liver resections also apply to Associating Liver Partition and Portal Vein Ligation for Staged Hepatectomy. With two operation steps, a prolonged operation time and higher use of blood transfusions a much higher incidence can be assumed. However, the prognostic value of renal function on the outcome in ALPPS patients could be much more severe than after conventional liver resections.

**Materials and methods:**

All patients included in the ALPPS Registry were screened for preoperative serum-creatinine levels (sCr) and were excluded if the value was not available.Interstage Renal Impairment (IRI) was defined as an increaseof Serum-Creatinine by >=0,3mg/dl referring to preoperative value or an increase of Serum-Creatinine by >=1,5x of the preoperative value on the fifth postoperative day after stage-1. Primary endpoint was the occurrence of renal impairment after stage-1. Secondary endpoints were morbidity and mortality after each stage.

**Results:**

We identified 741 patients for analysis. Overall, 7,5% had an IRI on postoperative day 5 after stage-1. Patients developing an IRI were significantly older and comorbidities such as myocardial infarction, cerebral vascular disease and liver diseases were significantly associated with IRI.

During stage-1, they had a significant longer operation duration, a higher proportion of intraoperative transfusions and additional procedures. After stage-1, IRI patients had more major complications and higher interstage mortality (1% vs. 8%, p<0,001). No differences were seen in volumetric analysis. There was no significant difference in the extension of the resection and operation duration of stage-2. Furthermore, patients with IRI are more likely to have more and severe complications after completion of stage-2.

The mortality in patients with an IRI is higher (38%) compared to non-IRI (8%, p<0,001), although the PHFL was not significant. However, in 41% of the patients with IRI the renal function recovered before stage-2, the mortality after stage-2 remained high (28%). The survival after completion of stage-2 was significantly lower for patients with IRI (Figure 1A, p<0,001). After exclusion of patients that died during hospital stay, there was no difference in survival (Figure 1B).

Risk factors for the development of an IRI were age over 67 years, a prolonged operation time over 5h and additional procedure during stage-1. However, IRI was no independent risk factor for mortality in this cohort.

**Conclusion:**

This study shows that interstage renal impairment is a prognostic factor for interstage and post-stage-2 morbidity and perioperative mortality and for the first time contributes risk factors for renal impairment after stage-1. However, if IRI causes the reduced outcome or serves a surrogate marker for major interstage complications remains unknown.

**Picture:**
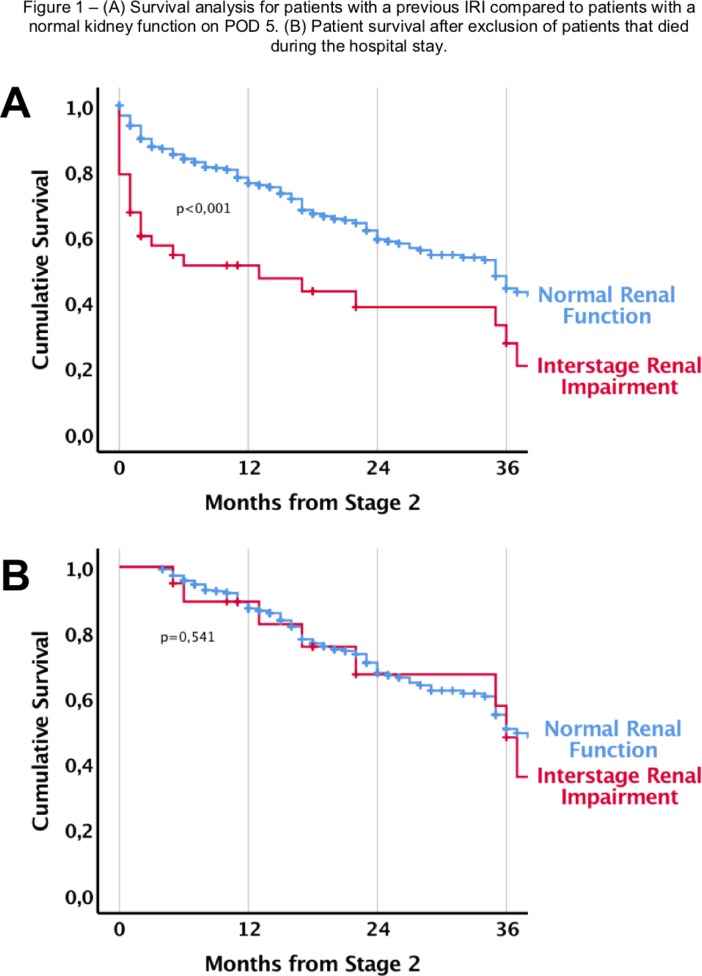
Figure 1

### Expression patterns of the anti-inflammatory protein Annexin A1 in anastomotic leakage

(Abstract ID: 681)

S. Reischl^1^, E. Miltschitzky^1^, A. Kasajima^1^, F. Becker^2^, E. Rijcken^2^, G. O. Ceyhan^1^, P.-A. Neumann^1^

^1^*Klinikum Rechts der Isar der TU München*

^2^*Universitätsklinikum Münster*

**Background:**

Annexin A1 (AnxA1) has been described as one of the main effector molecules of the anti-inflammatory effects of glucocorticoids. It exerts anti-inflammatory effects by suppression of leukocyte activation and transmigration, and exhibits anti-inflammatory properties in inflammation models that include endotoxemia, peritonitis and arthritis. Furthermore it has been described to play a role in intestinal wound healing. The effects on intestinal anastomotic healing and the cellular expression patterns in anastomotic tissue have not been analyzed yet. Thus we aimed to characterize the functional role of AnxA1 in intestinal anastomotic healing and further examine the cellular expression patterns of AnxA1 in anastomotic tissue.

**Materials and methods:**

To characterize the role of AnxA1 in intestinal anastomotic healing a surgical model of anastomotic leakage in mice has been established. A colonic anastomosis was performed in AnxA1 knockout mice (n=30) and wildtype controls (n=30). Anastomotic tissue was harvested after 1, 3 and 5 days and grade of inflammation (mild, medium, severe) was scored by a pathologist in a blinded fashion. To further examine cell specific expression patterns of AnxA1, immunohistochemistry (IHC) in slides of anastomotic tissue of human patients suffering from anastomotic leakage was performed. Expression of AnxA1 in anastomotic and non-anastomotic tissue areas was quantified. Furthermore immunofluorescence co-stainings of AnxA1 and different cell markers (CD20, CD4, CD8 and CD68) were used to identify the main sources of expression within the anastomotic tissue.

**Results:**

Increased histologic inflammation scores in AnxA1 null mice compared to wild type controls were found. The difference was more pronounced in the very early inflammatory period. IHC showed pronounced presence of AnxA1 positive lymphocytes within anastomotic area, whereas fibroblasts and endothelial cells showed only mild expression and epithelial cells showed no expression. The immunofluorescence co-staining revealed T cells highly positive for AnxA1 but B cells showed no expression.

**Conclusion:**

AnxA1 seems to be essential for uncomplicated intestinal anastomotic healing in mice. The expression of the anti-inflammatory protein AnxA1 was elevated in tissue of patients with proven anastomotic leakage, interestingly AnxA1 is mainly expressed by CD4 positive T cells. We will next evaluate the therapeutic potential of AnxA1 for improving intestinal anastomotic healing.

**Picture:**
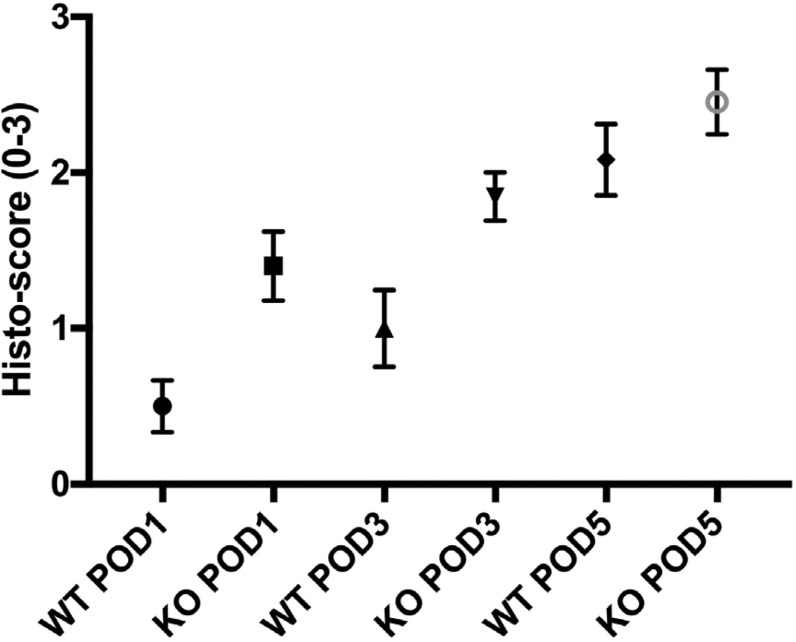
Histological scoring of anastomotic tissue of AnxA1 knockout (KO) and wildtype (WT) mice at postoperative day (POD) 1, 3 and 5 after colonic anastomosis

### Reliable prediction of the individual liver regeneration capacity by cytokine- and growthfactor profiling

(Abstract ID: 756)

K. Hoffmann^1^

^1^*Universitätsklinikum Heidelberg*

**Background:**

Post-hepatectomy liver failure (PHLF) is one of the most challenging complications following extended hepatectomy. Up to now it is not known if there is an individualized risk for impaired liver regeneration and which are potential targets for regeneration preconditioning. Cytokines and growth factor play a important role in every phase of liver reganeration. Aim of the study was to predict the individualized regeneration capacity of the liver by cytokine - and growth factor profiling.

**Materials and methods:**

Longitudinal blood samples (day -1, 1,3,7 realted to surgery) and liver tissue samples of 30 patients undergoing major liver resection were analysed. Primary human hepatocytes (PHH) were prepared out of tissue specimens for each patient. Cytokine and growth factor expression was analysed by Luminex bead-based multiplex assay, tissue protein expression by mass spectometry and PHH by Sybr Green Assay. Spearman-Correlation, Wilcoxon- and Kruskal-Wallis Test, Lasso Regression and Cytoscape were used for data analysis.

**Results:**

Preoperative status (ASA, Age, Co-morbidities) and postoperative complications correlate significantly with PHLF and mortality after liver resection. Expression of IL6, IL8, HGF, VEGF, EGF, APO2, PLGF and TGFß changes individually between day -1 and 7 after surgery. Correlation plotting identifies clear patterns associated with PHLF, complications Clavien Dindo >3a and exitus. Prediction models based on preoperative cytokine and growth factor expression predict the individual risk for PHLF. Clustering according to growth factor expression allows prediction of risk for death postoperatively. Combination of clinical parameters and cytokine/growth factor profile are surrogates for postoperative prognosis. A multifactorial interaction network was identified. Mass spectometry identified 158 significantly regulated proteins for liver failure. PHLF was associated with overexpression of 102 proteins e.g. HCCS, DDX47 and ITIH4 which are responsible for apoptotic signalling pathway, impaired response to cytokines as well as Oxidation-reduction process. In contrast patients with good liver regeneration overexpressed PTPN1(regulation of HGF signalling pathway), NHLRC2 (cell redox homeostasis) and GAK (regulation of cytokine response). Primary hepatocyte analysis revealed individual growth pattern upon stimulation with HGF and/or IL6. PHH growth capacity was significantly associated with postoperative liver function and clinical course.

**Conclusion:**

Cytokine- and growth factor profiling and modelling is a reliable method for preoperative prediction of the postoperative clinical outcome. The individual expression patterns correlate with the risk for PHLF. Validation on tissue level by massspectometry and functional level by primary hepatocytes supports the hypothesis of individual targeted regeneration preconditioning in the future.

**Picture:**
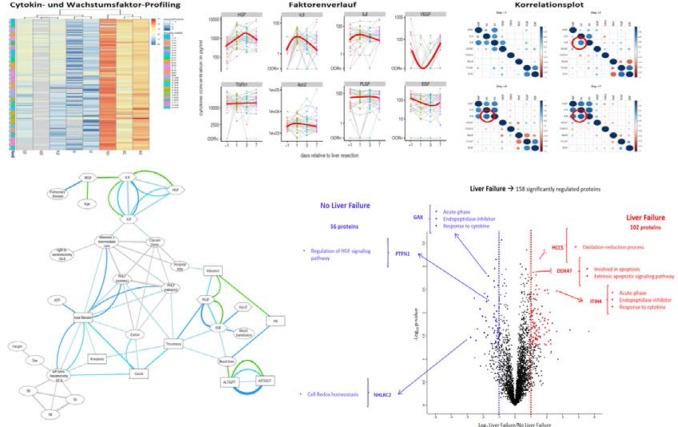


### Defunctioning ileostomy in rectal surgery – protection at any price?

(Abstract ID: 859)

S. Lünse^1^, A. Schreiber^1^, W. Keßler^1^, C.-D. Heidecke^1^, P. Menges^1^, L.-I. Partecke^1^

^1^*Universitätsmedizin Greifswald*

**Background:**

The loop ileostomy is commonly used for prevention of severe sepsis by fecal diversion due to anastomotic leakage after anterior rectal resection. It has been suggested that defunctioning ileostomy ameliorates the effects of a leak, which potentially leads to life-threatening pelvic sepsis. However, the presence of a stoma is connected to the risk of adverse effects related to the stoma itself as well as to the subsequent stoma closure.

**Materials and methods:**

We present a retrospective single-center analysis of 138 patients, who underwent a primary open anterior rectal resection with primary anastomosis and defunctioning loop ileostomy. It was divided into a group with an early stoma closure within 30 days after primary operation (group A, n=25) and into a group with the closure at a later date (group B, n=113). Primary endpoints were stenosis, bleeding, hernia, retraction, abscess and fistula of the stoma as well as paralysis, exsiccosis and colitis.

**Results:**

The most frequent adverse effect in both groups was the loss of fluid and electrolytes due to high stoma flow. Patients with a preoperative high morbidity of ASA III-IV showed a significant need for inpatient treatment of exsiccosis (p=0.037). Furthermore, patients with an early stoma closure (group A) showed a significant appearance of parastomal abscess (p=0.042), whereas parastomal hernia (p=0.038) and colitis (p= 0.012) were observed more frequently in patients with long-time stoma deviation (group B). Moreover, patients with late stoma closure showed significantly prolonged paralysis with the need for medical treatment (p=0.032).

**Conclusion:**

The construction of a defunctioning loop ileostomy during rectal resection is a safe and uncomplicated surgical procedure, but it can cause significant postoperative adverse effects. High fluid and electrolyte loss are well-known complications, but we herewith raise the evidence for parastomal hernia and colitis as well as for prolonged gut paralysis in patients with long-time defunctioning loop ileostomy. Therefore, the early stoma closure may avoid adverse effects that occur due to long-time deviation.

### The polymorphism rs8878 in CXCL10 is a predictor for relapse and survival in lung cancer

(Abstract ID: 863)

A. Polonski^1^, N. Haubold^1^, M. Bockhorn^1^, A. Heumann^1^, M. Reeh^1^, J. R. Izbicki^1^, F. G. Uzunoglu^1^

^1^*Universitätsklinikum Hamburg-Eppendorf*

**Background:**

CXCL10, a chemokine, has been attributed to several roles such as chemoattraction for monocytes/macrophages, promotion of T cell adhesion to endothelial cells, antitumor activity and angiogenesis. In our study, we aimed at assessing the prognostic relevance of the single-nucleotide polymorphism rs8878 in the CXCL10 gene in lung cancer patients.

**Materials and methods:**

Genomic DNA from blood and lymph node tissue samples from 186 lung cancer (squamous cell carcinoma and adenocarcinoma) patients was amplified and sequenced. The results were correlated with clinicopathological parameters and clinical outcome (disease free survival and overall survival). Chi-square test, Kaplan-Meier estimator and cox regression hazard model were used to assess the prognostic value.

**Results:**

C/C allele carriers had a significantly shorter overall survival (27,7 months vs 57 months; p=0,018) and a significantly shorter disease-free survival (22,1 months vs 41,6 months; p=0,029) compared to T/T and C/T allele carriers. Multivariate Cox regression identified the aberration as independent prognostic factor for UICC stadium II or III tumors (p<0,029) and R1 resection status (p<0,008).

**Conclusion:**

To conclude, determination of CXCL 10 preoperatively might potentially allow allocation of lung cancer patients into different risk profiles which might influence individual therapeutic strategies.

### Metastasis-associated fibroblasts promote tumor-angiogenesis in metastasized pancreatic cancer via Il-8 and CCL2 biological axes

(Abstract ID: 872)

T. Pausch^1^, E. Aue^1^, A. F. Valls^1^, Y. Shen^1^, M. Schneider^1^, T. Schmidt^1^

^1^*Universitätsklinikum Heidelberg*

**Background:**

Ductal adenocarcinoma of the pancreas (PDAC) as soon as metastasized cannot be cured by systemic therapy yet. Primary tumor and metastases exhibit a complex cancer pathology characterized by deposition of desmoplastic stroma, which contributes to cancer progression and chemoresistance. In this microenvironment, cancer cells engage in complex interactions with cancer-associated fibroblasts (CAFs). Concerning the role of CAFs it is heavily discussed if they support or fight the tumor. Even more the role of metastasis associated fibroblasts (MAFs) remains to be unclear and should be analyzed in this study.

**Materials and methods:**

In vitro we studied interaction of tumor cells of metastatic PDAC and fibroblasts in the context of tumor angiogenesis. We measured alterations of cell proteome via ELISA-proteome assay and proofed specific expression changes via qPCR. In angiogenesis assays we tried to block specifically expressed cytokine. In vivo we studied impact of antiangiogenic tyrosine kinase inhibitor Sunitinib on hepatic metastasis of PDAC and associated desmoplastic tumor microenvironment via immunohistochemistry.

**Results:**

Enhanced proangiogenic effect of co-cultured tumor cells and fibroblasts was seen. Additionally proliferation of fibroblasts was stimulated by tumor cells. Simultaneously cell proteome got augmented. Specifically up-regulation of cytokines interleukin 8 (Il-8/ CXCL8) and chemokine (C-C motif) ligand 2 (CCL2/ MCP-1) was seen in fibroblasts. Increased proangiogenic effects of co-cultures could be suppressed completely by blockage of IL-8- and CCL2-pathways. As anticipated in vivo Sunitinib led to reduction of metastasis. But beyond that it led to reduction of MAFs and simultaneously to increased proliferation of tumor cells in the field of micro-metastasis and cancer invasion front.

**Conclusion:**

Our results highlight that IL-8 and CCL2 from MAFs play a central role in tumor angiogenesis of metastasized PDAC which can be suppressed specifically. Nonspecific antiangiogenic therapy in fact reduces volume of metastases and number of MAFs yet it enhances aggressiveness of tumor cells.

### Esophageal adenocarcinoma: Circulating tumor cells in multimodal treatment protocols (ESO-CTC trial)

(Abstract ID: 887)

J. Kuvendjiska^1^, V. Martini^1^, P. Bronsert^2^, S. Timme^2^, B. Kulemann^1^, J. Höppner^1^

^1^*Universitätsklinikum Freiburg*

^2^*Universitätsklinikum Freiburg i.Br.*

**Background:**

Esophageal adenocarcinoma (EAC) is one of the most rapidly increasing tumor entities in the western world. Despite remarkable progress in the treatment of these patients, the overall outcome is still limited. More than 60 % of all patients who undergo an apparently curative, complete resection of a clinically non-metastatic primary tumor will eventually relapse or develop distant metastases, which might be caused by circulating tumor cells (CTC). However, there is little evidence about the significance of CTCs in patients with EAC.

Our study was designed as a pilot study of the ESOPEC-Trial. We evaluated the presence and morphology of CTCs during the treatment period and compared the well-established surface-antibody dependent CTC isolation technique (CellSearch®) with the isolation by size technique (ScreenCell®). The experimental results will be correlated with patients’ overall and relapse-free survival.

**Materials and methods:**

20 patients with non-metastatic EAC were consecutively enrolled into this trial prior to the beginning of the neoadjuvant treatment. The patients had no previous medical history of cancer. Blood specimen were sampled before the start of the neoadjuvant therapy (FLOT or CROSS protocol), after the neoadjuvant therapy, and finally after the surgery. CTC isolation was performed with CellSearch® isolation devices and by use of a cell size based filtration method (ScreenCell®). The cells isolated by size were subsequently stained with May-Grünwald Giemsa staining. We compared the results of the two CTC isolation techniques. Furthermore, the absolute number of CTCs per blood specimen was quantified and the morphology of both single and cluster CTCs during the time course of therapy was assessed.

**Results:**

75 % of the patient population was treated with neoadjuvant chemotherapy (FLOT protocol) while 25 % of the patient received radiochemotherapy (CROSS protocol). Using the ScreenCell® technique, single CTCs or cluster CTCs were found in 60 % of the patient population in at least one blood specimen. Interestingly, the number of CTC positive patients showed tendency to an increase after neoadjuvant therapy (before the neoadjuvant therapy: 30 %; after the neoadjuvant therapy 60%; after surgery: 62%). Additionally, we observed a tendency to an increase in the number of CTCs per/mL blood after the neoadjuvant therapy. However, due to the limited number of blood specimen no statistically significant differences could be observed during the time course of treatment. The CTCs showed a morphological diversity and could be grouped in four groups. However, CTC isolation by CellSearch® delivered unsatisfactory results since 40 % of the blood samples were non-analysable.

**Conclusion:**

In our study, we could observe a tendency to an increase of both the number of CTC positive patients and the absolute count of CTCs per ml after the neoadjuvant therapy. To our knowledge this is the first study to investigate the presence of CTCs during the time course of treatment in patients with EAC. The observed increase of CTCs after the therapy along with the role of the different morphological CTC subtypes will be further investigated in the patient population of the ESOPEC trial. Better isolation results were obtained using the cell size based filtration method (ScreenCell®). This supports the use of isolation by size, since CTCs (due to the epithelial-mesenchymal transition) might escape surface-antibody dependent isolation techniques.

### Dormancy in synchronous and metachronous colorectal liver metastasis

(Abstract ID: 933)

K. Jaber^1^, M. Wecker^1^, R. Bobe^1^, A. Rehders^1^, W. T. Knoefel^1^, G. Flügen^1^

^1^*Universitätsklinikum Düsseldorf*

**Background:**

Despite modern concepts in diagnosis and treatment, colorectal cancer (CRC) remains the third most common cancer worldwide. Although multimodal treatment is standard of care, the 5 years survival rate is just about 50%, most deaths resulting from metastatic spread. The Liver is the main organ in which those metastases disseminate. Even patients diagnosed with local disease (UICCI+II) will eventually develop CLM in 50% of the cases. In those patients, surgical therapy offers better survival and, in some cases, a total remission; yet even after total R0-resection, 60% of these patients will still suffer recurrent CLM within 2 years of initial treatment. Even after long periods of clinical remission, CLM can arise. In 2% of patients who have had successful treatment of M0 CRC and no recurrence or CLM in the following 5 years, CLM developed up to 10 years after initial therapy. This observed latency until overt CLM arise points to a clinically relevant pool of metastasis initiating cells in a dormant stage, present in the liver from an early stage. Currently, no treatment of these dormant disseminated tumor cells exists, as research into this phenomenon is lacking.

**Materials and methods:**

Using RNeasy FFPE Kit we isolated mRNA from FFPE sections of synchronous and metachronous CLM, as well as primary tumors (PT) (n=28patients). After producing cDNA from the mRNA-samples, using qPCR (quantitative real time PCR), we looked for the expression of known dormancy factors NRF2F1, DEC2, p27 and TGFβ2. These factors have previously been implicated in dormancy, as well as cell-cycle arrest. The δδC(t) method was used to analyze the qPCR results, statistics were carried out using two-tailed t-test with the Prism software (Version 6).

**Results:**

While observing interindividual variation, NR2F1 and TGFβ2 were not detectable in the majority of samples. Low sample acquisition of PT of patients with metachronous CLM has, so far, resulted in an insufficient number of samples of this group. Further recruitment is ongoing. Surprisingly, metachronous metastases so far showed a significantly lower expression of DEC2 than the synchronous CLM. We observed no significant difference in p27 expression between these two groups in the samples analyzed this far. Between PT and their synchronous CLM, we could not detect a significant difference in p27 expression, yet a trend toward higher expression in the CLM was observed. The DEC2 expression in the synchronous CLM was significantly lower than in the concurrent PT.

**Conclusion:**

While metachronous metastases have developed at a later time, compared to synchronous CLM, and the metastasis initiating cells may thus have gone through a dormancy-phase, all of the hepatic lesions included here were, at the time of resection, viable and proliferating metastases. Thus, the observed lower expression of the putative dormancy factors in metachronous metastases could be caused by a subclone that has been able to effectively exit dormancy. Following this hypothesis, heterogenous populations within synchronous metastases could express dormancy factors, as well. The ongoing recruitment of new samples will hopefully enable us to further unravel the connection between the established dormancy factors and the chronology of CLM. Tumor dormancy, especially in CLM, has not been extensively researched. Understanding dormancy is critical in developing treatment options for metastatic CRC and to stop metastasis in the first place.

### The predictive role of ctDNA for response to neoadjuvant chemoradiotherapy in locally advanced rectal cancer (NEORECT trial)

(Abstract ID: 937)

T. Grünewald^1^, S. Dintner^1^, M. Höck^1^, F. Sommer^1^, T. Kröncke^1^, H. Messmann^1^, M. Anthuber^1^, G. Stüben^1^, M. Trepel^1^, B. Märkl^1^, R. Claus^1^

^1^*Universitätsklinikum Augsburg*

**Background:**

Treatment of locally advanced rectal cancer (stage II or III) consists of neoadjuvant chemoradiotherapy (nCRT) followed by total mesorectal excision (TME). Complete pathologic remissions (pCR) are observed in 20-30% of patients (pts) undergoing nCRT. Several studies and case series comparing TME and "watch and wait" strategy after nCRT have reported similar excellent outcome for both patient groups (Kong et al. 2017). Thus, non-operative treatment for locally advanced rectal cancer might constitute a treatment option for selected pts. However, preoperative diagnostics including imaging, blood-derived protein biomarkers, histological and molecular markers have failed reliably predicting pCR. The detection of circulating tumor DNA (ctDNA) has proven to be sensitive for monitoring treatment response and detecting minimal residual disease (MRD). In a recent study, MRD assessment by ctDNA was successfully used for predicting relapse in stage II colon cancer demonstrating super predictive abilities compared to MRT and CT based follow-up. We hypothesized that monitoring ctDNA changes in pts with rectal cancer undergoing nCRT might facilitate identifying pts reaching pCR and thereby prospectively guide therapy.

**Materials and methods:**

We conducted a prospective single center study in pts with rectal cancer (stage II or III) planned for nCRT and curative resection. Serial peripheral blood samples were collected before, during and after nCRT and directly before TME. Circulating free DNA was extracted from 4 ml plasma. Informative somatic mutations were identified initially in rectal biopsies by next generation sequencing (Thermo Fisher Oncomine HotSpot Panel) and subsequently used for ctDNA quantification by digital PCR (dPCR; Thermo Fisher QuantStudio 3D Digital PCR System).

**Results:**

By the current interims analysis, 20 pts were included in the trial. Median age was 67 years (range 49-81), 60% were male. 7 pts who completed the trial protocol and reached surgery had ctDNA samples available for analysis. Of these, 4 pts had detectable ctDNA prior to therapy. Lower detection limit for dPCR assays on plasma from rectal cancer pts was established at a level of 0.1%. In 4 out of 4 pts, decrease of ctDNA was observable during nCRT. One out of 4 pts reached pCR, another patient achieved subtotal remission. One patient showed a continuous decline of mutant plasma DNA during nCRT (1.15%, 0.9% and 0.55%). Directly before TME, a 10-fold steep rise of mutant alleles (5.5%) was observed, which was consistent with new hepatic metastases. After resection of metastases, the mutant alleles decreased again. Taken together, our preliminary results indicate an interrelation between decrease of ctDNA and remission after nCRT.

**Conclusion:**

ctDNA is detectable in pts with stage II and III rectal cancer undergoing nCRT. ctDNA decrease can be observed upon nCRT and monitoring ctDNA dynamics during nCRT is a feasible approach to be further developed as predictive marker for achieving pCR.

## DGCH: Health politic

### Effect of transcutaneous vagus nerve stimulation on the gastrointestinal tract (transVaGa): A clinical pilot-trial

(Abstract ID: 770)

G.-S. Hong^1^, B. Pintea^2^, P. Lingohr^1^, N. Schäfer^3^, S. Wehner^1^, J. Kalff^1^, D. Pantelis^1^

^1^*Universitätsklinikum Bonn*

^2^*Universitätsklinikum BG Bergmannsheil Bochum*

^3^*Klinikum Leverkusen*

**Background:**

Postoperative ileus (POI) is a common complication after abdominal surgery. Invasive stimulation of the cervical vagus nerve is known to reduce inflammatory response and ameliorated POI after surgery in a mouse model. However, the transcutaneous vagus nerve stimulation (tVNS) is a possible non-invasive approach. Firsti we investigated to effect of tVNS in a mouse model, secondly, we aimed to investigate the effect of tVNS on the activation of the stomach muscle in humans in a clinical pilot trial.

**Materials and methods:**

Mice underwent abdominal surgery with intestinal manipulation. 24 hours after surgery, proinflammatory cytokines and leucocyte influx were investigated within the muscularis externa of the small bowel. To detect a functional effect of tVNS, gastrointestinal transit was assessed 24 hours after surgery. Secondly, we investigated the effect of tVNS in humans. Therefore, patients requiring open laparotomy were screened for a prospective proof of concept clinical study. After open laparotomy, muscle activity of the stomach was measured by a free running electromyography (EMG) before and during tVNS on the ear. Frequency and amplitude of compound gastric action potentials were the electrophysiological parameters we assessed to reveal the changes in electro motor gastric activity. Gastrin levels as a surrogate marker for vagus nerve activation was analyzed before, 1 and 3 hours after tVNS.

**Results:**

tVNS reduced significantly proinflammatory cytokine expression and leucocyte influx within the muscularis externa of the small bowel and improved gastrointestinal transit after abdominal surgery compared to sham-stimulation in mice. In humans, tVNS led to significant reduction of action potentials frequency and significant elevation of action potentials amplitude in the stomach compared to control. Gastrin levels were significantly elevated three hours after tVNS compared to levels before tVNS.

**Conclusion:**

Application of tVNS is a safe and feasible procedure during surgical intervention. Our results provide evidence that tVNS ameliorates postoperative ileus in mice and activates efferent visceral vagal fibers in humans. Therefore, this low risk and easy to perform method could be useful to prevent postoperative ileus.

## DGCH: Infections & hygiene

### OR ventilation a relevant factor for prevention of surgical site infections

(Abstract ID: 227)

C. Bulitta^1^, S. Buhl^1^

^1^*Ostbayerische Technische Hochschule Amberg-Weiden*

**Background:**

The role and requirement of ventilation technology in the operating theatre with regards to the control of indoor climate (including management of thermal loads) and ensuring aspects of occupational safety (removal of toxic substances) is beyond dispute. In contrast, the impact on the prevention of surgical site infections has been subject of controversial discussions for many years. Internationally uniform standards are missing. The current recommendations of the WHO and the German KRINKO for the prevention of surgical site infections do not see a compelling proof for the use of a low-turbulence displacement flow (TAV). The studies underlying these recommendations show weaknesses. This raises the question what is the most effective and economical concept for ventilation and air conditioning systems in the operating room.

**Materials and methods:**

We reviewed data in the literature as well as conducted own studies (active air sampling according to SIS-TS 39:2015 and particle measurments according to DIN 1946-4(2008)) in order to compare low-turbulence displacement flow (TAV), turbulent dilution flow (TVS) and temperature-controlled airflow (TAF) systems with different advantages and disadvantages regarding their impact on minimizing airborne risk factors for surgical site infections.

**Results:**

The results of active airsampling according to SIS-TS 39:2015 show that TAV and TAF, but not TVS, result in less than 10 cfu/m3 at all measurement locations in the room during surgery. Peripherally in the room, the cfu concentration was lowest for TAF. The cfu concentration did not scale proportionally with airflow rates. Regarding particle Measurments only TAV and TAF are in compliance with the required protection degree levels according to DIN 1946-4(2008). Compared with TAV, the power consumption of TAF is lower and there was significantly less disturbance from noise and draught.

**Conclusion:**

If you pay particular attention to the avoidance or minimization of potential risk factors for surgical site infections, you should select a technical solution that reduces the microbiological burden in the air of the operating theatre. Taking into account current publications and our data TAV and TAF are both suitable for this purpose. However, based on our findings and the current data in the literature ultimately, the operator, together with the responsible hospital hygienist, has to evaluate and define the ventilation system to be implemented. This is a risk management decision and has to consider the utilization concept and the planned clinical procedures for the respectiv operating theatre.

### Reduction of postoperative wound infections by antiseptica (RECIPE) – a prospective randomized trial

(Abstract ID: 555)

J. C. Lauscher^1^, R. Strobel^1^, M. Leonhardt^1^, A. Böckenfeld^1^, K. Neumann^2^, F. Speichinger^1^, S. Daum^1^, M. E. Kreis^1^

^1^*Charité - Unversitätsmedizin Berlin CBF*

^2^*Charité - Universitätsmedizin Berlin CCM*

**Background:**

Postoperative wound infection is a common complication after laparotomy causing increased morbidity and postoperative pain. Although many surgical departments conduct subcutaneous wound irrigation before skin closure in order to reduce wound infections, there is a lack of high-level evidence on the use of wound irrigation for the prevention of postoperative wound infection. Therefore, we conducted an investigator initiated randomized trial comparing antiseptic wound irrigation with Serasept® with saline.

**Materials and methods:**

The RECIPE trial is a single-centre, prospective, randomized-controlled trial with two parallel treatment groups, comparing subcutaneous wound irrigation with Serasept® (0.04 % Polihexanide) to irrigation with saline 0.9% after elective laparotomy. The primary endpoint is the rate of wound infection within 30 days postoperatively according to the criteria of the Centers for Disease Control (CDC). Secondary endpoints are colonisation of abdominal wall with bacteria, length of hospital stay, postoperative pain and cosmetic result.

**Results:**

Between Feb. 2015, and Jun. 2018, 456 patients were randomly assigned to Serasept® (n=228) or saline (n=228), of whom 393 patients (191 in the Serasept® group and 202 in the saline group) were analyzed. Overall, we recorded 84 (21.4%) postoperative wound infections. On univariate analysis, significantly fewer wound infections occurred in the Serasept® group: 29 (15.2%) vs. 55 (27.2%); p = 0.004. On multivariate analysis, subcutaneous irrigation (OR 0.41, 95% CI 0.23 - 0.72, p = 0.002) was the parameter with the strongest risk reduction for postoperative wound infection. Preoperative anaemia (OR 1.85, 95% CI 1.03 - 3.31, p = 0.039) and intraoperative creation of an ostomy (OR 2.29, 95% CI 1.12 - 4.69, p = 0.023) were also risk factors for postoperative wound infections. With regards to secondary outcomes, there were fewer positive swabs of the abdominal wall after irrigation with Serasept®: 35 (21.6%) vs. 107 (67.7%); p < 0.001. No differences were seen between the two groups in terms of length of hospital stay (median Serasept® 13.0 vs. saline 14.0 days; p = 0.943), postoperative pain (Serasept® 7.8% vs. saline 6.4% strong pain; p = 0.814) and cosmetic result (Serasept® 9.0% vs. saline 9.9% unsatisfied; p = 0.647).

**Conclusion:**

Subcutaneous wound irrigation with Serasept® reduces postoperative wound infections after elective laparotomy in visceral surgery. Furthermore, it reduces the colonisation of the abdominal wall with bacteria. Preoperative anaemia and intraoperative creation of an ostomy were also associated with postoperative wound infections.

## DGCH: Innovations and robotics

### Cassandra – Machine learning algorithms in visceral surgery

(Abstract ID: 315)

A. Winter^1^, F. Krenzien^1^, M. Maurer^1^, S. Chopra^1^, M. Schmelzle^1^, J. Pratschke^1^, I. M. Sauer^1^

^1^*Charité - Universitätsmedizin Berlin CVK*

**Background:**

Artificial intelligence, big data and machine learning algorithms are currently changing the way we look at medical data fundamentally. These technologies create new and fascinating possibilities for the interpretation and evaluation of a large number of clinical data. With their help doctors are able to discover patterns and complex relationships. However, a concrete implementation in clinical practice, especially in surgery, is still to find.

**Materials and methods:**

At the Department of Surgery, Charité - Universitätsmedizin Berlin, we are implementing machine learning algorithms for early detection of postoperative complication after major abdominal surgery. After abdominal surgery, signs of complication often occur delayed und indifferent, but once a patient shows signs of a severe complication, we are often behind the action, we are only able to react instead of act. The idea of this project is to collect all data from patients before and after pancreas, liver and colorectal surgery and analyse these data with machine learning algorithms. With the help of these algorithms, we hope to detect major postoperative complications at a much earlier stage. The heart of every machine learning algorithm is the quality of the underlying data. On an ICU these data can be easily obtained, because there is a continuous monitoring of the patient’s vital signs, status etc. To achieve the required quality of the data on a normal surgical ward we needed to completely rethink and redesign the way we do our clinical documentation and develop our digital infrastructure.

**Results:**

We analyzed all our processes and mandatory documentation in our clinic and looked for synergies between the different documentation systems. Due to this analysis we came up with 50 patient-related, 18-22 disease-related (depending on the disease) and 41- 48 procedure-related factors (depending on the kind of surgery). For the postoperative course we identified 39-59 (depending whether blood samples were taken or not) parameters which were assessed every day. The next step was to find automated interfaces to document these factors and parameters in a standardized way. We identified 4 relevant forms for the documentation of the required data. These forms where specially designed for an easy standardized documentation for a desktop computer and tablet device layout. We the help of these specially designed forms we were able to generate a data set of approximately 300 data points until POD 4 per patients. All these previously described parameters were assessed one time a day, but the dynamic in a patient’s postoperative course is missing. In cooperation with a berlin start up company we designed a wearable device for continuous measurement of patient’s vital signs: Heart rate, blood pressure temperature, oxygen saturation. With this setup we are now able generate a sufficient standardized data set, which covers every aspect of the patient’s course.

**Conclusion:**

The implementation of machine learning algorithms in daily surgical routine requires high effort in the planning and designing of the clinical documentation. In addition investments in the digital infrastructure are necessary. The effort for this is enormous, but the resulting advantages for patients and medical staff can be a game changer.

### 3D-navigated liver resection – optimizing technical aspects for clinical use

(Abstract ID: 432)

R. Wahba^1^, G. Dieplinger^1^, A. Bunck^1^, C. J. Bruns^1^, D. Stippel^1^

^1^*Uniklinik Köln*

**Background:**

Intraoperative 3D-naviagtion is an innovative technique, that could help to perform parenchyma-sparing liver resection (3DNvL) in combination with microwave ablation (3DNvMWA) in case with multiple disseminated metastases. Many different technical details occurred to be important to guarantee an optimal use during the operation. Aim of this study is to analyze these aspects.

**Materials and methods:**

Prospective observational study during introduction of 3DNvL/3DNvMWA

**Results:**

After test runs 3DNvL was first performed in October 2017. Since then it was used in 10 cases. Data reconstruction was performed by MeViS. In one patient 3DNvL was not possible due to extensive alterations of the liver surface. The mean operation time was 294 ± 60min, the mean number of treated metastases was 7.1 ± 5.9. In 7 patients resection and microwave ablation was combined. In 3 patients 3DNvL was used to support parenchyma dissection in major anatomical resections (right hepatectomy, extended right hepatectomy, ALPPS). Registration of reference points was performed in apnoe. A combination of surface and intrahepatic ultrasound points was most precise (screenshot technique) compared to the sweep technique (dynamic registration of vessel via ultrasound). 3DNavL and 3DNvMWA was feasible in central located liver segments, which could be assessed without deforming the liver. 3DNvMWA should be well planned upfront, beginning at the caudal segments and be completed before resection. 3DNvMWA without ultrasound guidance was feasible also in lateral dorsal a parts of segments 7 and 8. Short ablation devices (14cm) could interfere with the navigation tools, so longer devices (up to 29 cm) should be available. Treated lesions should be marked on the navigation system to ease later orientation in the liver. Smaller surgical wound retractors with less steel ease the use of 3DNvL. Fixing the reference tool on a plastic covered ultrasound head did not compromise navigation. Actual software update allowed in-house local data reconstruction. The acceptance of 3DNavL by the scrub nurses during implementation increased with continuous use.

**Conclusion:**

3D-navigation could optimize surgical treatment of multiple hepatic lesions. Technical details are important in the daily use to reach precision.

**Picture:**
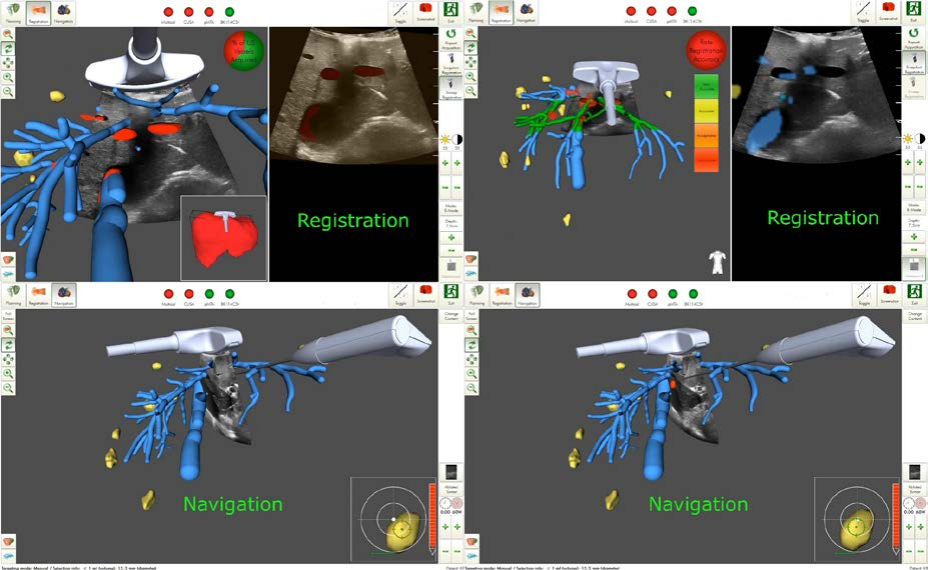
Screenshots during liver surgery: planning, registration and 3D-navigated microwave ablation of a liver metastasis

### CT-based cinematic vs. volume rendering of multifragmentary intraarticular lower extremity fractures: Does it improve preoperative visualization of fracture morphology?

(Abstract ID: 440)

L. Wollschläger^1^, J. Boos^1^, P. Jungbluth^1^, J. Grassmann^1^, C. Schleich^1^, D. Latz^1^, P. Kroepil^1^, G. Antoch^1^, J. Windolf^1^, B. Schaarschmidt^1^

^1^*Universitätsklinikum Düsseldorf*

**Background:**

Using a unique lightning model, cinematic rendering (CR), a new 3D rendering technique, converts conventional CT image datasets into photo-realistic 3D reconstructions. The aim of this study was to compare CR images of multifragmentary intraarticular lower extremity fractures with standard volume rendering technique (VRT) images to evaluate the potential of CR in fracture visualization.

**Materials and methods:**

In this retrospective, IRB-approved study, 41 patients (female: n=13; male: n=28; mean age: 52.3±17.9y) with multifragmentary intraarticular fractures of the lower extremity (calcaneus: n=16, tibial pilon: n=19, acetabulum: n=6) were included. All CT datasets were acquired on a 128-row dual source CT. Using a dedicated workstation VRT and CR images were reconstructed. Independently, two experienced board-certified traumatologists trained in special trauma surgery reviewed VRT and CR images. On a 6-point Likert scale (1=non-diagnostic to 6=excellent) image quality, anatomical accuracy and fracture visualization were rated. Axial CT images with MPR were used as reference standard. Additionally, the advantage of CR over VRT images for visualization of fracture morphology was assessed. Median values between both readers were calculated for each score. Wilcoxon-Ranksum test was performed to compare both reconstruction methods (p<0.05 indicating statistical significance).

**Results:**

In comparison to VRT, CR had a higher image quality (VRT: 2.5; CR: 6.0; p<0.001), a higher anatomical accuracy (VRT: 3.5; CR: 5.5; p<0.001) and provided a more detailed fracture visualization (VRT: 2.5; CR: 6.0; p<0.001). An additional value of CR for preoperative fracture visualization -- was reported in 65.9% (27/41) of all patients in comparison to VRT.

**Conclusion:**

CR is superior to VRT due to higher image quality and higher anatomical accuracy. Because of improved visualization of lower extremity fractures, CR reconstructions should be used for fracture demonstration in interdisciplinary conferences.

**Picture:**
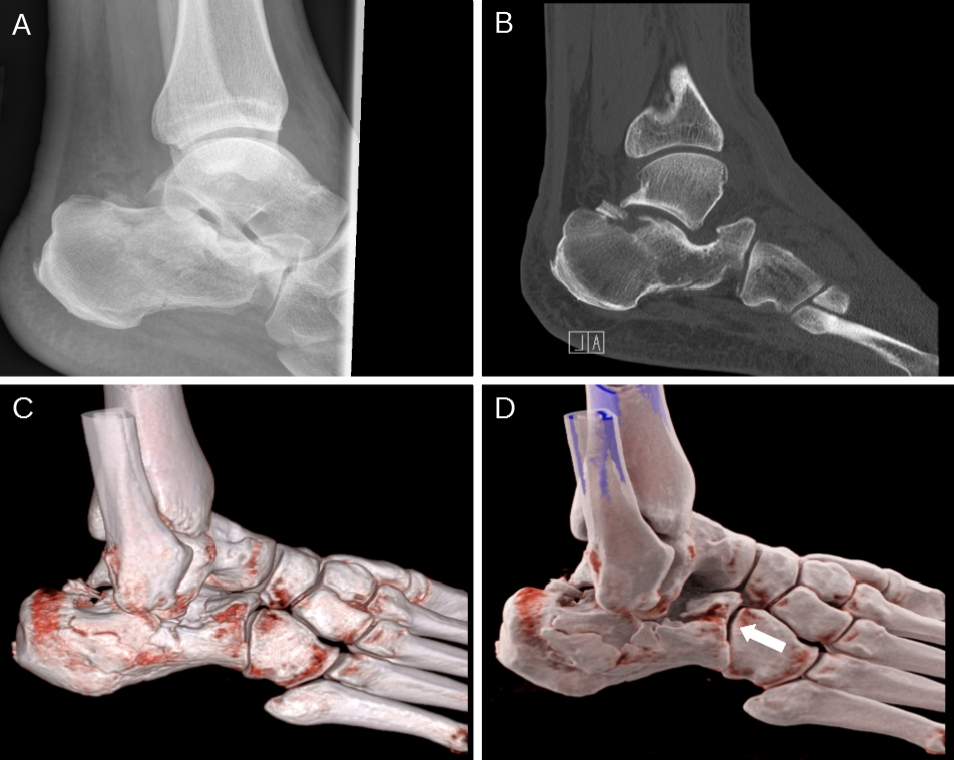
Fig. 1 A 58-year-old male patient who had suffered ankle distorsion after falling down a flight of stairs. Conventional (A) and sagittal MPR images of the CT data set (B) are displayed. While the fracture of the anterior process of the calcaneus is difficult to delineate in the VRT image (C), CR reconstructions (D) clearly demonstrate the fracture lines (white arrow).

### First experience routinely using ICG near-infrared fluorescence angiography for visualization of the bowel perfusion in colorectal surgery to minimize the risk of anastomotic leakage

(Abstract ID: 643)

A. Buia^1^, B. Albers^1^, E. Hanisch^1^

^1^*Asklepios Klinik Langen*

**Background:**

In up to 19 % of colorectal resections an anastomotic leakage is clinically evident followed by a reintervention rate in up to 80 %. The consequence is a considerable increase in morbidity and mortality. Ischemia of the anastomosis is described as one of the main independent risk factors. Thus, routinely testing of the bowel perfusion with the near-infrared fluorescence technique using indocyanine-green (ICG) should minimize this risk factor potentially leading to a significant reduction of the anastomotic leakage rate.

**Materials and methods:**

Indocyanine-green (ICG) is an indicator dye with the ability to fluoresce after activation with infrared light. In medicine this technique is used after intravenous application routinely e.g. for the examination of the retina perfusion in ophthalmology. Newer indications are the examination of the perfusion of gastrointestinal anastomosis or e.g. to clarify intraoperatively the anatomy of the biliary duct or to show the location of the ureter. From February to September 2018 a standardized ICG-imaging of the bowel was performed in 67 patients with colorectal surgery before and after establishing an anastomosis.First, an imaging of the resection line of the bowel followed by an examination with an introduced head of a circular stapler (if applicable) was accomplished. Finally, the perfusion of the anastomosis was checked. In case a low quality of perfusion is detected, the plane of resection area was changed.

**Results:**

The median age of the patients was 67y. (35-96 y.; 32 male / 35 female). The risk stratification according to the ASA Score (American Society of Anaesthesiology) was ASA1 n=3, ASA2 n=46, and ASA 3 n=18 patients. 55 procedures were elective surgery, 18 procedures were emergency operations. The indication for operation was benign in n=30 cases, in n=37 cases malignant.Procedures: ileocoecal resection (n=7), right hemicolectomy (n=17), sigmoid- and rectosigmoid resections (n= 30), sigmoid resection with rectopexy (n=1), anterior rectum resection (n=5), transanal total mesorectal excision (TATME) (n=3) and reversal of Hartmann´s procedure (n=3). 18 procedures were operated openly, 49 procedures were performed laparoscopically.Intraoperatively a low perfusion was detected in n=3 patients and the resection plane was changed into an area of good perfusion. In these three cases there was no anastomotic leakage observed postoperatively.Overall 2 cases of anastomotic leakage were detected (2,99%). One Patient was clinically inapparent and the leakage was detected after 60 days in the context of diagnostic for a reversal procedure of a protective ileostomy (clavien dindo complication score grade 1). The second anastomotic leakage was apparent on the second postoperative day (clavien dindo complication score grade 3). After the retrospectively analysis of the video documentation of this case with a very early leakage we interpret this event as a technical malfunction of the used stapler which was not detected during the procedure.

**Conclusion:**

The standardized imaging of the bowel perfusion before and after performing gastrointestinal anastomosis might be a promising Our results have to be interpreted with caution due to the small sample size. But the ease of performing this procedure with tolerable costs for an additional valuable information for the surgeon can lead intraoperatively to a change of the intraoperative strategy finally leading to increase in patient safety.

### Photorealistic 3D visualizations (Cinematic Rendering) of CT and MR data improve the comprehension of the patient anatomy

(Abstract ID: 651)

M. Elshafei^1^, J. Binder^1^, J. Baecker^1^, M. Uder^1^, R. Grützmann^1^, C. Krautz^1^

^1^*Universitätsklinikum Erlangen*

**Background:**

Cinematic rendering, a novel 3D visualization technology for post-processing of computed tomography (CT) or magnetic resonance (MR), provides a natural and photorealistic representation of the patient anatomy. Research on potential advantages for the comprehension of the surgical anatomy is not available. We aimed to determine the value of cinematic rendering (CR) for the comprehension of the surgical anatomy in general surgery.

**Materials and methods:**

In a crossover fashion, eighteen surgeons with different expertise (9 residents, 9 attendants) evaluated CR and conventional images of 40 general surgery cases. The initial image modality of each case was assigned by random sequence. All surgeons had to answer questions addressing the patients’ anatomy in regards to crucial aspects of pre-operative planning or intraoperative strategies. In addition, a multiple-item questionnaire was applied to rate participants’ perceived advantage of using CR compared to conventional images.

**Results:**

Visualization with CR allowed a more correct and faster comprehension of the surgical anatomy. Average time spent by participants was significantly shorter with CR imaging compared to CT scans (49.4s [95%-CI 43.7-55.8] vs. 86.4s [77.1-96.3]; p<0.001). The percentage of correct answers was significantly higher with CR compared to CT (99.2% vs. 88.5%). Notably, less experienced residents perceived the highest benefit (CR: 99.2% correct answers in 54.4s [45.0-63.8] vs. CT: 86.9% correct answers in 104.6s [91.4-117.9]). Analysis of the self-assessment questionnaire showed that CR adds significant value for the comprehension of the surgical anatomy (Table 1). No carryover or period effects were observed.

**Conclusion:**

In this study, visualization with CR allowed a more correct and faster comprehension of the surgical anatomy compared to conventional CT imaging. In particular, less experienced residents benefitted from this visualization tool.

**Picture:**
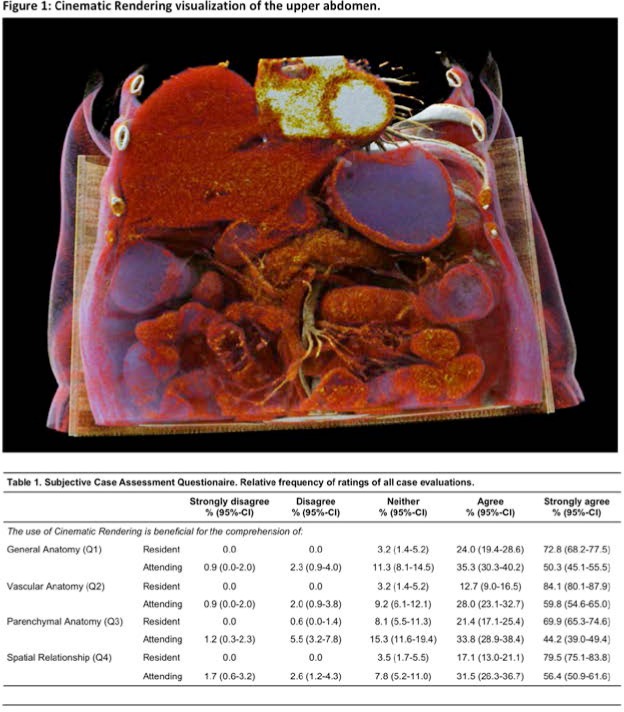


### Augmented Hyperspectral Imaging: A modified technology for the evaluation of tissue perfusion and integrity

(Abstract ID: 910)

A. Studier-Fischer^1^, K.-F. Kowalewski^1^, F. M. Schwab^1^, B. P. Müller-Stich^1^, F. Nickel^1^

^1^*Universitätsklinikum Heidelberg*

**Background:**

The evaluation of tissue perfusion and integrity is a key component of successful surgery minimizing major complications such as tissue necrosis and anastomic insufficiency.

Despite great efforts from medical-tech companies and research groups, the simple visual aspect of the tissue still is the method of choice for most surgeons.

**Materials and methods:**

Hyperspectral Imaging (HSI) is a novel imaging technique that addresses this issue. It works by projecting white light of a wide spectrum from 500 to 995 nm wavelength onto tissue in a field of view measuring 25 x 30 cm. The integrated camera then receives signals that are reflected by the underlying tissue. This reflection is tissue-specific and depends on oxygen saturation, haemoglobin concentration, water content, tissue temperature and a variety of other influencing factors. The camera obtains the signal intensities for each individual pixel in 5 nm steps resulting in 100 measured values for each pixel. Certain substances have characteristic absorption spectra with maxima and minima at very specific wavelengths e.g. deoxyhaemoglobin with its maximum at 555 nm. Eventually, a computer calculates indices which are presented in artificially coloured images that are overlaid onto the original photograph of the recorded site.

For this experimental animal study in a porcine model, the TIVITA™ tissue system HSI Camera is used. Indices that are directly provided by this system are oxygen saturation, tissue haemoglobin, near-infrared and tisse water. In preliminary works, it could be shown that the sensitivity of these indices is sufficient for clinical evaluation. Neither contrast agent nor ionizing radiation has to be applied for this type of imaging technique.

**Results:**

Further development of this technology resulted in augmented hyperspectral imaging (AHSI). Dyes with characteristic absorption spectra such as methylene blue (MB), toluidine blue (TB) and indocyanine green (ICG) are combined with conventional hyperspectral imaging allowing for an improved evaluation of perfusion situation, anastomic leakage and stenosis.

The datacubes were imported into a Python script and own indices that were developed by preceding photometry of the dyes were applied resulting in pictures that were much more sensitive for the identification of dyes compared to the human eye.

**Conclusion:**

AHSI is a novel system for intraoperative real-time evaluation of tissue perfusion for specific organs. Its sensitivity for ischemic regions is greater than visual inspection and has the potential to be beneficial for surgical outcomes.

## DGCH: Quality and transparency

### Quantity and quality of randomized controlled trials in pancreatic surgery – an analysis of more than 3 decades

(Abstract ID: 768)

F. J. Hüttner^1^, L. Capdeville^1^, P. Probst^1^, M. W. Büchler^1^, M. K. Diener^1^

^1^*Universitätsklinikum Heidelberg*

**Background:**

Clinical research in surgery, and surgical randomized controlled trials (RCTs) in particular, represent specific difficulties and have therefore suffered from limited quality for a long time. These challenges are even pronounced in complex surgical procedures such as pancreatic surgery. The aim of the current analysis was to systematically evaluate the quantity and quality of RCTs in pancreatic surgery.

**Materials and methods:**

A systematic literature search in the databases PubMed, Cochrane CENTRAL and Web of Science was performed to identify all RCTs considering pancreatic surgery. Quantity and quality was compared between three periods (P-I: before 1996; P-II: 1996-2007; P-III: after 2008). Extracted data, including basic trial data and quality measures, were organized in a relational database and evidence maps were created to identify lack of evidence for particular fields.

**Results:**

After thorough literature screening by two independent reviewers, a total of 246 RCTs comprising data on > 26,000 patients were included in the analysis. Quantity of RCTs in pancreatic surgery increased steadily over the study period. Methodological quality, excempted the domain ’blinding’ of the Cochrane Collaboration’s risk of bias tool, also improved significantly during the latter segments of the study period. Most trials were from Europe (46.3%), followed by Asia (35.0%) and North America (14.2%). Evidence mapping identified limited evidence from RCTs for pancreatic surgical procedures apart from pancreatoduodenectomy and for specific diseases such as intraductal papillary mucinous neoplasms and neuroendocrine neoplasms.

**Conclusion:**

RCTs considering pancreatic surgery demonstrated increasing quantity, but also improved quality in recent years. The evidence maps identified several evidence gaps, which could guide prioritization of future clinical research in pancreatic surgery.

## DGCH: Risk and error management

### Calculating the Risk - Can we accurately measure the perioperative Risk?

(Abstract ID: 728)

M. Boyce^1^, P. M. Vogt^2^

^1^*Medical One Klinik Stuttgart*

^2^*Medizinische Hochschule Hannover*

**Background:**

Over 230 million major surgical operations are performed each year worldwide and major morbidity complicates 3-16% of all inpatient surgical procedures in developed countries, in developing countries, studies suggest a death rate of 5-10% for major surgery. The knowledge of potential risk and known risk factors is essential for surgical decision making and obtaining consent of the patient.Calculating the perioperative risk in surgical patients often relied on expertise of the surgeon and complication factors known from the literature. Many are guided by the American society of Anaesthesiologists (ASA) classification. The American college of surgeons developed a preoperative surgical risk score (Surgeons National Surgical Quality Improvement Program, ACS-NSQIP) which calculates the perioperative risk for a patient for a specific procedure. This program has collected data from 393 hospitals. It allows the surgeon to calculate the perioperative risk for a specific procedure predicting 8 outcomes.

**Materials and methods:**

From May 2016 until May 2017 we collected risk data on all inpatient who received surgical treatment. Excluded were children under the age of 16 and patients with major burns. 1049 patients were included in the study. All patient data was collected retrospectively. Patients data was entered into the open access ACS-Risk calculator. The individual risk scores were calculated and entered into a database. Inpatient adverse events as well as 30 days outcome data was collected and compared with the estimated risk predicted by the risk calculator. Statistical analysis was performed using the SPSS performing descriptive and multi regression analysis.

**Results:**

The mean age was 45,43 (Range 16-94 years). 617 (58,8%) patients were male and 432 (41,2%) were female. 486 (46,3%) were elective cases and 563 (53,7%) were emergency cases. The majority of patients were ASA II (n=471; 44,9%) and ASA I (n=419, 39,9%). 146 were classified as ASA III (13,9%) and only 16 as ASA IV (1,5%). The minority of patients were diabetic (n=85, 8,1%) were as 965 (91,9%) not. 765 (72,9%) had no history of hypertension with 285 patients (27,1%) being known to have hypertension. 22,3% (n=234) were current smokers with 77,5% (n=814) being non-smokers. The mean BMI was 25,61kg/m2 (Range 11-53), whilst 651 (62%) had a BMI of >/= 25kg/m2. 24,1% showed an overall complication which was not significantly different to the risk predicted by the calculator. This was also true at predicting surgical site infections. Other parameters like Predicted length of stay were significantly different.

**Conclusion:**

The use of surgical risk calculators has sparked recent discussions about its reliability. For years surgeons have relied on statistical outcome data and their own gut feeling. The introduction of prefabricated calculators should help guide patient selection and consent. However they are not universally usable and caution must be taken when interpreting their calculated data.

## DGCH: tumor therapy

### Platinum-based neoadjuvant chemoradiotherapy in patients with stage II/III rectal cancer does not improve overall survival – a systematic review and meta-analysis

(Abstract ID: 50)

F. J. Hüttner^1^, E. Kalkum^1^, M. Hackbusch^1^, P. Probst^1^, M. W. Büchler^1^, M. K. Diener^1^

^1^*Universitätsklinikum Heidelberg*

**Background:**

Current guidelines recommend neoadjuvant therapy in terms of short-course radiotherapy or chemoradiotherapy (CRT) for patients with stage II/III rectal cancer. Neoadjuvant therapy has been shown to reduce rates of local recurrence, while it has failed to improve overall survival. Intensified treatment regimens such as addition of platinum derivatives to fluoropyrimidine-based CRT have been frequently investigated but their role in patients with stage II/III rectal cancer remains controversial.

**Materials and methods:**

A systematic literature search of the databases MEDLINE (PubMed), Cochrane Library and Web of Science was performed to identify randomized controlled trials (RCTs) comparing fluoropyrimidine-based CRT with or without the addition of a platinum cytotoxic agent. Trial selection, data extraction and quality assessment were performed by two reviewers independently according to the recommendations of the Cochrane Collaboration. Main endpoints were overall and disease-free survival and further outcomes included pathologic complete response, local and distant recurrences, toxicity, perioperative morbidity and treatment compliance. Time-to-event data were pooled as hazard ratios by the inverse variance method and binary outcomes were aggregated as odds ratios by means of the Peto method.

**Results:**

Ten RCTs with a total of 5521 patients were included in the meta-analysis. Addition of platinum derivatives did not improve overall survival (HR 0.91, 95% CI 0.80-1.03, p=0.12) but significantly improved disease-free survival (HR 0.89, 95% CI 0.81-0.99, p=0.02) and pathological complete response (OR 1.30, 95% CI 1.10-1.55, p<0.01). While there was no difference in local recurrence (OR 0.83, 95% CI 0.65-1.05, p=0.12), distant recurrence was significantly reduced (OR 0.79, 95% CI 0.67-0.94, p<0.01). These benefits were accompanied by a higher rate of grade 3/4 toxicity

**Conclusion:**

Intensified neoadjuvant CRT with addition of a platinum cytotoxic agent did not improve overall survival in patients with stage II/III rectal cancer. The improvement in disease-free survival, rates of distant recurrence and pathologic complete response is achieved at the cost of a substantially increased toxicity. Thus, neoadjuvant CRT with addition of a platinum derivative cannot be recommended comprehensively but may be considered as treatment option in patients with high-risk preoperative situations.

### Radical surgery for locally advanced pancreatic cancer after neoadjuvant treatment: prognostic factors of survical

(Abstract ID: 135)

U. Klaiber^1^, E. Schnaidt^1^, U. Heger^1^, M. Sachsenmaier^1^, U. Hinz^1^, A. L. Mihaljevic^1^, O. Strobel^1^, M.W. Büchler^1^, T. Hackert^1^

^1^*Universitätsklinikum Heidelberg*

**Background:**

In patients undergoing surgery following neoadjuvant treatment for pancreatic ductal adenocarcinoma (PDAC), the risk factors influencing prognosis have not been adequately investigated. For patients undergoing upfront surgery for primary resectable PDAC, the resection margin (R) status has been shown to be a relevant determinant of postoperative survival. The aim of this study was to evaluate the impact of clinical and pathological factors including R status on survival in patients undergoing pancreatic surgery following neoadjuvant treatment for locally advanced PDAC.

**Materials and methods:**

Prospectively collected data of consecutive patients undergoing pancreatic resection following neoadjuvant treatment for locally advanced PDAC were analyzed. R status was categorized as R0 (tumor-free margin >=1 mm), R0 CRM+ (circumferential margin positive = tumor-free margin <1 mm), and R1 (microscopic tumor infiltration at the resection margin). Clinico-pathological characteristics and outcomes were compared among these groups. The R status and other patient, tumor, and resection characteristics were tested for survival prediction.

**Results:**

Between January 2006 and February 2017, 296 patients with locally advanced PDAC underwent tumor resection following neoadjuvant treatment. Sixteen patients not fulfilling the inclusion criteria were excluded from analysis. Thus, the final study population consisted of 280 patients (138 men, 142 women). The R status was R0 in 82 (29.3%) patients, R0 CRM+ in 99 (35.4%) patients, and R1 in 99 (35.4%) patients. Median overall survival from the time of surgery was 25.1 months (R0) vs. 15.3 months (R0 CRM+) vs. 18.6 months (R1), with 3-year overall survival rates of 35.0%, 20.7%, and 16.1%, respectively (P=0.0076). Median disease-free survival from the time of surgery was 9.8 months (R0) vs. 8.9 months (R0 CRM+) vs. 8.3 months (R1), with 3-year disease-free survival rates of 19.6%, 9.8%, and 5.9%, respectively (P = 0.0250). Median duration of the neoadjuvant treatment period was 5.1 months. In multivariable analyses, preoperative serum CA 19-9 levels, number of tumor-infiltrated lymph nodes, distant metastases, and vascular infiltration were significant prognostic factors for overall and disease-free survival. The R status was not confirmed to be a significant prognostic factor in multivariable analyses.

**Conclusion:**

In the neoadjuvant setting, preoperative serum levels of CA 19-9, vascular invasion, and number of tumor-positive lymph nodes are independent factors of survival. In contrast to upfront surgery for resectable PDAC, however, the R status is not an independent determinant of survival in the neoadjuvant setting.

### Circulating miRNAs in patients with rectal cancer during preoperative chemoradiotherapy for predicting response and monitoring development of the disease

(Abstract ID: 201)

A. Azizian^1^, K. Max^2^, K. A. Bogardus^2^, M. Grade^1^, M. Ghadimi^1^, T. Tuschl^2^, J. Gaedcke^1^

^1^*Universitätsmedizin Göttingen*

^2^*The Rockefeller University, New York City*

**Background:**

Patients with locally advanced rectal cancer are treated with preoperative chemoradiotherapy (CRT) followed by surgical resection (total mesorectal excision, TME). Although patients have similar clinical parameters (uT2-3, uN+) and receive standard therapy, their response to CRT and their prognosis differ widely. Biomarkers to predict therapy response and to reveal early possible recurrence of the disease would be of great importance for individualizing therapy and improving prognosis. MicroRNAs (miRNAs) are single-stranded small non-coding RNAs representing master regulators of gene expression which perform post-transcriptional modification of mRNA. In many diseases including different cancer types the importance of miRNAs has been shown.

**Materials and methods:**

Blood samples of 45 patients with locally advanced rectal cancer have been collected at several time points: before any therapy, after a week of CRT, after 4 weeks of CRT, after surgery and finally several months after completed therapy. All patients have been treated according the control arm of the CAO/ARO/AIO-04 study receiving radiotherapy of 50.4 Gy in 28 fractions plus i.v. 5-FU (1g/m2during days 1-5 and 29-33). Matched tumor biopsies have been collected before any therapy.

Using a new developed method for RNA purification out of body fluids we isolated high amounts of RNA out of plasma samples. All samples for purification were processed fully-automated by a fully equipped liquid epMotion 5075 liquid handling system which ensured a clean and accurate pipetting. To verify the separation of RNA and DNA, radiolabelled markers were used as quality control.The cDNA libraries were then generated by RT-PCR and sequenced in a single Illumina HiSeq lane yielding approximately 150 million reads. RNA quality and recovery efficiencies were determined using two different synthetic RNA calibrator sets.

All tissue samples were frozen in liquid nitrogen. A fraction of each sample was used for determining the percentage of tumor within the biopsy. RNA purification out of tumor biopsies was performed via a modified trizol-based method. The quality of the isolated RNA was verified via bioanalyzer.

**Results:**

Comparing miRNA profile of the rectal cancer patients to normal controls, there is a set of differentially abundant miRNAs. Those miRNAs show an adjusted abundance in patients’ samples taken after completed therapy: In those samples the miRNA profile resembles to the miRNA profile of normal controls.

Two miRNAs were identified to predict tumor response in pretherapeutic blood samples und were also detectable in the matched tumor tissue biopsies: miR-577 has a significantly higher abundance in plasma of patients with tumor regression grade 4 (complete response) while miR-506 has a significantly higher abundance in plasma of patients with tumor regression grade 1 (non-/poor response). Using both miRNAs prediction of response is possible with high accuracy.

**Conclusion:**

Using an accurate purification and sequencing method, miRNA profiles in plasma can distinguish between rectal cancer patients and normal controls, and also between patients during CRT and tumor-free patients after completed therapy. This can be used to monitor patients after therapy and detect possible recurrence via blood based method. Furthermore, miR-577 and miR-506 can predict good vs. poor response to CRT in pretherapeutic samples and might allow an individualized therapy. A validation set including further 40 patients has been collected and will be processed.

### The prognostic capacity of CBP and p300 in rectal cancer

(Abstract ID: 202)

F. Rühlmann^1^, I.-M. Windhof-Jaidhauser^1^, C. Menze^1^, H. Bohnenberger^1^, T. Beißbarth^1^, M. Ghadimi^1^, S. Dango^2^

^1^*Universitätsmedizin Göttingen*

^2^*Kreisklinikum Siegen*

**Background:**

The transcriptional coactivators and histone acetyltransferases (HAT) CREB-binding protein (CBP) and its homolog p300 are involved in many cellular processes including DNA repair, cell growth, differentiation and apoptosis. Despite their general function as tumor suppressors, their role in human colorectal cancer (CRC) remains unclear and contradictory. We correlated CBP and p300 expression from human rectal cancer tissue with clinicopathological parameters to evaluate a possible potential for epigenetic-based anti-cancer therapy.

**Materials and methods:**

This analysis involved 93 patients (median: 70 years; 63 men (67.7%), 30 women (32.3%)) with locally advanced rectal cancer. All these patients underwent primary resection and were treated within the phase-II GAST-05 trial. The expression of CBP and p300 was determined by immunohistochemical staining from resection specimens using the H-Score. It was then correlated with both clinical parameters and long-term follow-up.

**Results:**

High CBP expression in resection specimens significantly correlated with a longer cancer- specific survival (CSS; p=0.002). Regarding CSS, CBP represents an independent prognostic parameter in univariate analysis in this collective (p=0.042). We did not find any correlation between CBP expression and tumor stage or grading or tumor localization. In our analysis we could demonstrate that approximately two thirds of the patients highly express nucleus CBP. Low expression of p300 correlates non-significantly with shorter CSS (p=0.09). A cooperativeness between CBP and p300 was not observed in this collective of patients.

**Conclusion:**

High CBP expression was significantly correlated to a better long-term outcome und could therefore represent a new target in upcoming therapeutic strategies as HDAC inhibitors have entered the clinical arena. These findings should be further evaluated and verified in further clinical trials.

### Radical surgery with high cumulative radiation doses is associated with enhanced local control in retroperitoneal soft tissue sarcoma

(Abstract ID: 212)

F. Willis^1^, S. Schimmack^1^, M. W. Büchler^1^, M. Uhl^1^, T. Schmidt^1^, G. Mechtersheimer^1^, U. Hinz^1^, M. Schneider^1^

^1^*Universitätsklinikum Heidelberg*

**Background:**

Retroperitoneal soft tissue sarcoma (rSTS) are characterized by high local recurrence (LR) rates. Known prognostic factors comprise tumor entity, histological grading and resection margins. Despite aggressive surgical approaches wide resection margins cannot be achieved in all patients. There is evidence that recurrence rates can be improved by additional radiation therapy, but data concerning the significance of intraoperative radiation therapy (IORT) are scarce. We aimed to identify prognostic factors affecting LR after surgical resection of rSTS, and to evaluate the significance of IORT in multimodal treatment of rSTS.

**Materials and methods:**

Patients undergoing resection of primary, recurrent or metastasized retroperitoneal STS at the University of Heidelberg Department of General, Visceral and Transplantation Surgery were retrospectively analyzed. Univariate Kaplan-Meyer and multivariate Cox regression analyses were performed to identify predictors of LR free survival, and to investigate the impact of IORT on recurrence rates.

**Results:**

280 patients were identified. The median LR free survival of all 280 patients was 45.04 ±7.82 months (95% CI: 29.71 - 60.37). 3-year LR-free survival was 56% and 5-year LR free survival was 43%. Recurrent and metastatic tumors as well as histology of dedifferentiated liposarcoma or unclassified sarcoma were associated with decreased LR free survival. Cumulative radiation doses exceeding 60 Gy were associated with lower LR rates. This effect was especially pronounced in primary tumors and tumors with gross complete but microscopic positive resection margins (R1 resection). Application of IORT alone did not influence LR free survival.

**Conclusion:**

There seems to be no benefit of applying IORT alone in patients with rSTS. However, patients may benefit from cumulative radiation doses exceeding 60 Gy, which can hardly be achieved applying external beam radiation therapy (EBRT) alone. IORT may therefore represent an indispensable part in multimodal therapy of retroperitoneal STS.

**Picture:**
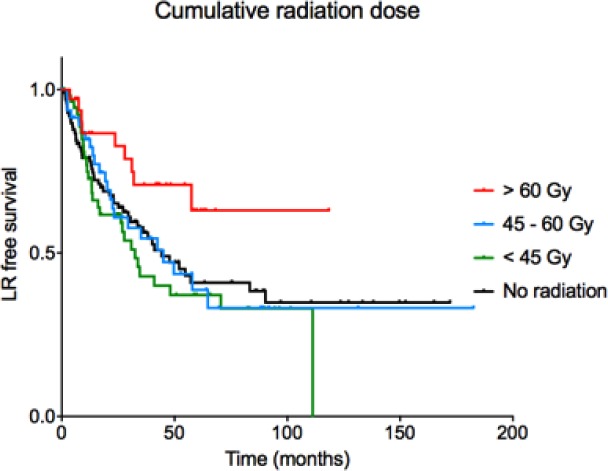
Local recurrence free survival by cumulative radiation dose with estimated 5 year local recurrence free survival: > 60 Gy: 63%, 45-60 Gy: 39%, < 45 Gy: 37%, no radiation: 41%.

### Tumor-specific cellular and humoral immune responses following microwave ablation (MWA) in patients with hepatocellular Carcinoma (HCC)

(Abstract ID: 221)

E. Staib^1^, K. Leuchte^1^, M. Thelen^1^, R. R. Datta^1^, D. L. Stippel^1^, C. J. Bruns^1^, M. S. von Bergwelt-Baildon^1^, K. Wennhold^1^, H. A. Schlößer^1^

^1^*University of Cologne, Köln*

**Background:**

Thermal ablative therapies, such as microwave ablation (MWA) are standard treatments for HCC. In addition to the local tumor destruction, abscopal effects (a reduction of the tumor mass in areas that were not included in the thermal ablation) could be observed. These systemic effects may be mediated by anti-tumor immune response.

**Materials and methods:**

PBMC of patients receiving MWA were collected prospectively on day 0, 7 and 90. We included PBMC samples of 14 patients with long-term-remission (>1year) and 12 patients with early relapse (<1 year). Immune-related effects of MWA in HCC were analysed by flow cytometry and Fluorospot assays (8 tumor associated antigens). Digital analyses of scanned IHC slides was used to determine the "Immunoscore" of primary tumors in patients receiving MWA and resection.

**Results:**

Flow cytometric analyses of prospective patients revealed only moderate effects of microwave ablation on circulating immune cell subsets. Fluorospot analyses against 8 cancer testis antigens frequently described in HCC, revealed de-novo (5/19) or enhanced (2/19) tumor-specific immune responses in a subset of prospective patients. Patients with a long-lasting remission (>12 months) after MWA more often showed antigen-specific T cell responses than patients suffering from an early relapse (5/14 vs. 0/12 patients). Correlations of response to MWA and IHC-based analyses of immunogenicity will also be presented.

**Conclusion:**

In summary, we provide first evidence for an induction of tumor-specific immune response by MWA. Combination of MWA and immune checkpoint inhibition could be a promising therapeutic approach in HCC.

### Patterns of immune checkpoint expression by primary tumor cells and tumor infiltrating lymphocytes across different tumor entities

(Abstract ID: 222)

M. Thelen^1^, K. Wennhold^1^, A. Quaas^1^, E. Staib^1^, P. Plum^1^, D. Pfister^1^, F. Dörr^1^, M. Heldwein^1^, K. Hekmat^1^, F. Thangarajah^1^, M. Malter^1^, D. Ratiu^1^, C. J. Bruns^1^, M. S. von Bergwelt-Baildon^2^, H. A. Schlößer^1^

^1^*University of Cologne, Köln*

^2^*Uniklinik München*

**Background:**

Immune-checkpoint inhibition (CKI) demonstrated breakthrough therapeutic efficacy in several kinds of cancer. These therapies are unique, as the primary target is not the tumor cell itself, but the crosstalk between immune cells and cancer cells in the tumor microenvironment. Efficacy of CKI is not limited to patients with expression of the respective protein on tumor cells and recent publications demonstrated that expression of PD-L1 on tumor-infiltrating lymphocytes (TIL) can be of similar importance.

**Materials and methods:**

Expression patterns of 30 described immune checkpoint and regulatory molecules were analyzed on T, B and NK cells in peripheral blood and single cell suspensions of normal tissue and primary tumor samples of 130 patients. Furthermore, expression of the respective ligands on primary tumor cells was assessed in tissue microarrays. Tumors deriving from 11 different primary tissues were included.

**Results:**

Tumors from different origins contained distinct patterns of other tumor-infiltrating lymphocytes, while regulatory T cells were increased in tumors from all sites. The majority of analyzed immune checkpoint pathways could be detected in the tumor microenvironment. Despite the variety of primary tumor sites, our analyses revealed similar expression patterns for a large fraction of molecules included in this study. Expression patterns on lymphocytic subsets were largely overlapping and will be demonstrated in detail. Analyses of immunosuppressive ligands revealed multiple co-expressions for the majority of patients of different tissue sites.

**Conclusion:**

Immune escape is a common feature of cancer and the specific expression patterns described in this study are of translational relevance for ongoing and future immunotherapeutic trials.

### Two-staged CRS & HIPEC could optimize learning curve and complication rates during implementation in a tertiary teaching hospital

(Abstract ID: 268)

J. Bohle^1^, P. S. Plum^1^, R. R. Datta^1^, A. Tuchscherer^1^, T. Zander^1^, D. L. Stippel^1^, C. J. Bruns^1^, R. Wahba^1^

^1^*Uniklinik Köln*

**Background:**

Since establishment of cytoreductive surgery (CRS) and hyperthermic intraperitoneal chemotherapy (HIPEC) within the modern multimodal oncological management of advanced solid tumor diseases, the patients’ prognosis could be improved. However, these procedures are associated with increased patients’ morbidity. While splitting CRS an HIPEC (two-staged procedure) the surgical trauma could be minimized. Therefore, this study analyzed our results of two-staged CRS and HIPEC during reimplementation in a tertiary teaching hospital.

**Materials and methods:**

Since its reimplementation in March 2016, 40 patients underwent CRS and HIPEC due to primary gastrointestinal neoplasia as well as gynecological tumors. Data retrospectively analyzed utilizing the clinical documentation system as well as the active patients’ follow-up data within our outpatient clinics. Severe postsurgical complications (Dindo-Clavien Grade III and IV) and other clinic-technical factors (e.g. ICU stay, duration of HIPEC, time delay between CRS and HIPEC) were considered.

**Results:**

The majority of patients (n=22; 55%) underwent CRS & HIPEC due to colorectal cancer, 10 patients had gastric cancer (25%), 3 patients (7.5%) suffered from ovarian cancer, 2 patients had pseudomyxoma (5%), one patient showed neuroendocrine carcinoma (NEC) of the ovar (2.5%), one patient had a neuroendocrine tumor (NET) in the same localization (2.5%) and one patient had a cholangiocellular carcinoma (2.5%). Initial peritoneal carcinoma index (PCI) was median 12 and ranged from 9 to 31 after diagnostic laparoscopy and 4 to 36 according to CT-morphology. Time delay was median 2,73 days between CRS and HIPEC (range: 0-10 days). Surgery duration was 125-741 min (median: 359 min) for CRS and 60-187 min (median: 137.5 min) for HIPEC. Patients stayed on ICU 1-10 days (median: 3,03 days) after CRS and 2-12 days (median: 2,00 days) after HIPEC. In 10 patients (25%) complications according to Dindo-Clavien grade III occurred while complications according to Dindo-Clavien grade IV manifested in 6 patients (15%). Complications were mechanical ileus in 3 patients (7.5%), wound dehiscence in 11 patients (27.5%), small bowel perforation in 4 patients (10%) and leakage of the blind rectum after resection in one patient (2.5%). However, there was no anastomotic leakage detectable at all (0%). The 30-days and 90-days mortality was 0% in the cohort.

**Conclusion:**

The two-staged CRS and HIPEC concept is a feasible method to optimize the learning curve and results in low severe complication rates during implementation of these technical challenging procedures in a tertiary teaching hospital.

### Low expression of CK1δ in colon tumor tissue predicts longer survival in colorectal cancer patients

(Abstract ID: 368)

P. Xu^1^, J. Richter^1^, A. Hillenbrand^1^, J. Lemke^1^, D. Henne-Bruns^1^, U. Knippschild^1^

^1^*Universitätsklinikum Ulm*

**Background:**

Colorectal cancer (CRC) is the fourth contributor of cancer related mortality worldwide due to high apoptotic resistance and metastatic potential. Casein kinase 1 (CK1) family are eukaryotic evolutionary conserved and ubiquitously expressed serine/threonine-specific protein kinases. In human, six isoforms of CK1 α, γ1, γ2, γ3, δ, and ε were identified. CK1 play a key role in regulating tumor development and tumor suppressor functions as well as the contribution of CK1 family members in pathways associated with growth, development, and homeostasis. Therefore, inhibition of CK1 isoforms in CRC development and progression, is a potential way to develop drug targets in the treatment of CRC.

**Materials and methods:**

CK1δ expression level in tumor tissue specimens of 326 CRC patients was performed by RT-PCR analyses. For analyzing survival rates of patients with tumors expressing low and high CK1δ RNA levels, Kaplan-Meier curves were compared using the log-rank test. Immunohistochemical stainings of CK1δ in randomly chosen colorectal tumor tissue to confirm a correlation between CK1δ RNA and protein expression levels.

**Results:**

We found weak CK1δ immunoreactivity in patients with low CK1δ RNA levels as well as strong CK1δ immunoreactivity in patients with high CK1δ RNA levels, confirming a correlation between CK1δ RNA and protein expression levels. CK1δ RNA expression levels are analyzed in tumors of the entire cohort revealing significantly increased survival rates of patients with lower expression of CK1δ in whole cohort (p = 0.040). Increased survival rates of patients with low CK1δ RNA levels are also find in subgroup of patients with grade 1 and grade 2 tumors (p = 0.039), in patients with tumors in right colon (p =0.008), in male patients with right-sided tumors (p=0.004) and in patients with tumors assigned to UICC II and UICC III tumors and located in right colon (p< 0.001).

**Conclusion:**

Our results provide evidence that decreased CK1δ expression levels in tumor tissue predict prolonged survival rates. Therefore, CK1δ can be considered as a prognostic biomarker in CRC patients, consequently providing an interesting drug target for new therapy concepts.

### Giant hemangioma of the lumbar spine: A case report

(Abstract ID: 416)

G. Schmid^1^, A. Reinke^1^

^1^*Donau-Ries Klinik Donauwörth*

**Background:**

Vertebral hemangiomas are common lesions, which are often seen as incidental findings, and are usually benign. Rare cases of aggressive hemangiomas are reported with compression fractures and neurological deficits. We present a highly unusual case of a 54-year old man with paraparesis and an infiltrative tumor in the lumbar spine.

**Materials and methods:**

A 54-year old mal presented an acute sciatic pain and a paraparesis of his legs. Magnetic resonance imaging (MRI) revealed a massive tumor mass of the entire vertebral body of L3 and the surrounding tissue (diameter 65 mm) with subtotal compression of the spinal canal. Angiographic computed tomography (CT) showed a hypervascular bony lesion with loss of height of L3. Tumor staging could not prove any other neoplasia. We performed a dorsal stabilization with Carbon Instrumentation and decompression in case of emergency. After primary improvement of the symptoms a re-paraparesis of the legs appeared after a few days. The postoperative MRI demonstrated a recurrent increase of the tumor with compression of the spinal canal. After endovascular embolization a vertebral body replacement of L3 with tumor resection from ventral was done.

**Results:**

Patient recovered completely from his neurological deficit after the second operation. Final pathologic diagnosis after surgery confirmed the diagnosis of an osseous hemangioma.

**Conclusion:**

This case highlights an extremely unusual appearance of a giant and aggressive vertebral hemangioma with consecutive fracture. In addition, it shows the importance of preoperative MRI and vascular imaging as well as the necessary urgent surgical management.

### R0-resection following chemotherapy or chemoradiation improves survival of primary inoperable pancreatic cancer patients. Interim results of the German CONKO-007 prospective randomized multicenter trial

(Abstract ID: 909)

R. Grützmann^1^, S. Kersting^1^, H. Golcher^1^, D. Lubgan^1^, R. Fietkau^1^, U. Wittel^1^

^1^*Universitätsklinikum Erlangen*

**Background:**

Pancreatic cancer is the fourth leading cause of cancer-related death in men and women, and its incidence is steadily increasing. Surgery - the only potentially curative treatment - is reserved for patients with initially resectable tumors. However, only around 15.0% of all patients are classified as resectable at primary diagnosis. It remains unclear whether patients with initially unresectable pancreatic cancer can achieve resectable status and, if so, whether surgery provides them any additional benefit. The CONKO-007 study examines the value of radiotherapy versus chemotherapy in patients with non-metastatic, unresectable pancreatic cancer.

**Materials and methods:**

CONKO-007 is an open-label, multicenter, phase III randomized clinical trial which examines the effectiveness of chemoradiotherapy compared to chemotherapy alone after induction chemotherapy with 3 cycles of gemcitabine or 6 cycles of FOLFIRINOX in patients with non-metastatic, initially locally advanced unresectable pancreatic cancer.

The present analysis was based on data from the first 180 patients recruited from 2013 to December 2015. The patients were consecutively enrolled by 10 large cancer centers in Germany, each of which recruited more than 6 patients/center.

**Results:**

Induction chemotherapy consisted of gemcitabine in 43 cases and of FOLFIRINOX in 137 cases. Compliance was excellent in both groups. After completion of induction chemotherapy, 126/180 patients (70.0%) were randomized to further treatment. The main reasons for non-randomization were distant metastasis (29.6%) and patient request (24.1%), followed by local progression 216 (13.0%), side effects (13.0%), insufficient dose of induction chemotherapy (9.3%), concomitant disease (9.3%), and treatment switch (1.9%). After completion of study treatment, 36/126 patients (20.0%) underwent surgery and 87 (48.3%) received no surgical treatment. Of the surgically treated patients, R0 resection was achieved in 25 cases (20.0% of the randomized patients and 13.9% of the overall group of 180 study patients) versus exploration or R1/R2/Rx resection in the remaining 11 cases (6.1%). Five (13.9%) of the 36 surgically treated patients developed postoperative complications of grade 3 severity or worse: bleeding (n=1), pancreatic fistula (n=1), wound healing disturbance 228 (n=1), ileus (n=1), and insufficiency of gastric anastomosis (n=1). Two (5.5%) of the 36 patients died from complications: one died on the day after surgery (acute liver failure), and the other died 36 days after surgery (multiple organ failure with sepsis). Disease-free survival (DFS) was 11.2 months in the overall population compared to 16.6 months in the R0 resection group. The prognosis of patients who achieved R0 resection was thus significantly better (p=0.003) than that of non-operated patients (DFS: 11.0 months) or patients in the exploration or R1/R2/Rx resection group (DFS: 11.9 months). At 24-month follow-up, the median tumor-free survival rate was 28.0% (95.0% Cl: 14.9-52.5) in the R0 resection group, 10.0% (95.0% Cl: 8.6-0.20.9) in the non-operated group, and 0.0% in the exploration or R1/R2/Rx resection group (p=0.003).

**Conclusion:**

Our data show that even if pancreatic cancer is staged as locally advanced unresectable at primary diagnosis, tumor resectability should be reassessed after neoadjuvant treatment. Patients with a good probability of R0 resection should undergo surgery as this significantly improves their prognosis.

**Picture:**
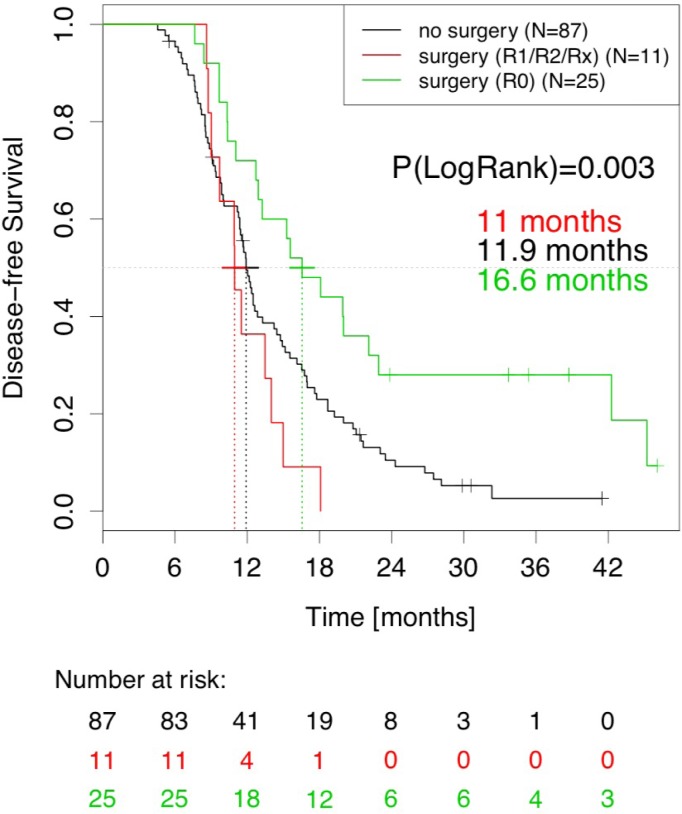


## DGCH: Varia

### What happens under that dressing? Improvement of local microcirculation through intermittent Negative Pressure Wound Therapy

(Abstract ID: 56)

A. Sogorski^1^, J. Kolbenschlag^2^, M. Dadras^1^, C. Wallner^1^, B. Behr^1^, N. Kapalschinski^1^, A. Daigeler^2^, M. Lehnhardt^1^

^1^*Universitätsklinikum BG Bergmannsheil Bochum*

^2^*BG Unfallklinik Tübingen*

**Background:**

Negative Pressure Wound Therapy (NPWT) is widely used across different kinds of surgical disciplines. A controversial debate was raised by diverging results from studies that were conducted to examine the impact of NPWT on local perfusion. Thus, there is a lack of evidence for one important underlying factor that influences the physiology of wound healing under an applied NPWT-dressing. The commonly used measurement technique, the Laser Doppler Flowmetry (LD), turned out as a method that is rather complicated to interpret when the measurement probes are subjected to a certain degree of compressional forces. Beyond that, majority of studies conducted in humans investigated the continuous mode of NPWT application, although there was evidence for a superior effect on wound healing of the intermittent mode of action already mentioned in the initial publications of Morykwas et al. who introduced the concept of NPWT.

Objecitve: To investigate the immediate local perfusion changes due to an applied intermittent NPWT protocol through utilization of combined LD and white light spectroscopy (TS).

**Materials and methods:**

A NPWT dressing was applied to the antero-lateral thigh of seven healthy volunteers with two probes of both pressure and microcirculatory measuring devices. One of each probe was placed under the NPWT dressing, the other one in close proximity next to it. A protocol consisting of two cycles of 10 minutes of -125mmHg pressure, followed by 10 minutes of 0 mmHg pressure was applied. Measurements of local pressure to the underlying tissue, as well as microcirculatory changes (Blood flow, tissue oxygen saturation, blood flow velocity and relative hemoglobin content) were performed continuously.

**Results:**

Applied negative pressure caused significant compressional forces (27.33 mmHg, p<0.05) towards the underlying tissue. Blood Flow showed to be increased after both suction periods (+ 52.5 %, + 108.7 %; p<0.05) and continued increasing until the end of measurements (+ 145.3 %). This was accompanied by an also significant increase in Tissue Oxygen Saturation (+ 21.6 %; p<0.05) and Relative Hemoglobin Content (+ 16.7 %). Next to the dressing, changes were also significant but less pronounced.

**Conclusion:**

Intermittent NPWT improves local microcirculation with consecutive enhancement of oxygen supply. Combined LD and TS is suitable for analysis of microcirculation under an applied NPWT-dressing. Further research is warranted to determine the differences of microcirculatory changes due to continuous vs. intermittent mode of action.

### Weight Regain after Laparoscopic Sleeve Gastrectomy (LSG): Our Experience with the SADI-Bypass in Comparison to the Omega-loop bypass (OLB)

(Abstract ID: 423)

M. de la Cruz^1^, M. Büsing^1^, J. Halter^1^, V. Christogianni^1^

^1^*Knappschaftkrankenhaus Recklinghausen*

**Background:**

The laparoscopic sleeve gastrectomy (LSG) is an established procedure for the treatment of morbid obesity. Alongside with the gastroesophageal reflux, the regain of weight or inadequate weight loss, especially in patients with a BMI greater than 60 kg/m2 are considered to be the two most frequent long term problems. In particular, the GERD represents the main long term complication of the Omega loop bypass (OLB), consequently a conversional bypass to a Roux-Y-Bypass has to be performed.

**Materials and methods:**

Since August 2014 the SADI-S procedure has been one of the established operations in our clinic for weight regain or insufficient weight loss after LSG. The hand sutured duodenoileal anastomosis is performed 250cm oral of the ileoceacal valve. A Re-Sleeve resection or an inverting longitudinal suture is simultaneously performed in cases of dilatatjon of the Sleeve. The OLB is created with a 250cm measured biliary thigh, starting from the Lig. suspensorium duodeni over a gastrojejunal anastomosis. Patients experiencing GERD Symptoms, due to a hiatal hernia, are also treated with a posterior hiatoplasty.

**Results:**

In this study we have compared two groups of patients. The first cohort included 46 patients, who underwent a SADI-S operation, the second group consisted of 39 patients receiving an OLB. The time of the initial procedure (LSG), prior to the SADI operation, was 37 months (11-96 months). In comparison, the OLB shows a median period of 33 months (4-240 months).

Group one showed a mean preoperative BMI of 40,6 kg/m2 (25,3 -65,6 kg/m2). Group two showed a similar number of 41,1 kg/m2 (21,6-67,7 kg/m2).

In the long term view, nearly all patients of group one showed satisfactory weight loss with a BMI-drop after at least two years of 8,5 kg/m2 and a corresponding %EWL of 68,6, indicating a succesful treatment. The GERD symptoms were reduced and in most cases no further PPI medication was needed anymore. Excessive diarrhoea or flatulences were not observed.

The BMI drop in group two was similar to the SADI at 8,1 kg/m2, whereas the %EWL was insufficient at 41,5. Three patients complained about a dumping syndrom.

In regard to the SADI the following procedures were performed at the same time: Re-Sleeve (n=1), gastroplication (n=9), hiatoplasty (n=9) and cholecystectomy (n=20).

There was no case of a duodenal stump insufficiency, whereas in one case a reanastomosis, due to a duodenalileal insufficiency, was necessary. The mean hospital stay took 5 days.

In case of the OLB the following procedures where simultaneously performed: Hiatoplasty (n=16), adhesiolysis (n=17), modified fundoplicatio (n=1) and cholecystectomy (n=2). Three operations were performed due to a proximal insufficiency, after a sleeve gastrectomy. In seven cases a conversion to a Roux-Y-Bypass had to be done, because of persistent reflux symptoms. The mean hospital stay took also 5 days.

**Conclusion:**

The SADI-S operation is a more effective procedure than the OLB, when it comes for the treatment of inadequate weight loss after a sleeve gastrectomy, with less postoperative complaints. A volume reduction of the sleeve can additionaly be performed. In cases of gastroesophageal reflux a hiatoplasty can also provide a relief of the symptoms. A long term follow up is necessary.

### Negative sentinel node biopsie in melanoma patients: How high is the risk for recurrence?

(Abstract ID: 467)

V. Schellerer^1^, J. Göhl^1^, M. Erdmann^1^, R. Grützmann^1^, G. Schuler^1^

^1^*Universitätsklinikum Erlangen*

**Background:**

For about 25 years, the sentinel node biopsy (SNB) is the gold standard in staging of malignant melanoma. The result of the SNB is one of the main prognostic factors for survival. Anyway, patients with negative SNB might develop further relapse of the malignant melanoma. We analyzed our data of patients suffering from recurrent metastasis despite negative SNB.

**Materials and methods:**

Between 1/2008 and 12/2013 545 patients received SNB for malignant melanoma. In 449 cases the result of the sentinel node was negative. These patients were followed till 1/2016 and recurrence of melanoma was evaluated.

**Results:**

Between 1/2008 and 12/2013 in 449 patients SNB was negative. Out of this group 72 Patients (16%) presented with recurrent melanoma till 1/2016: 47 patients (10%) had loco-regional recurrence including 25 patients with recurrence within the lymph node region of the initial sentinel node biopsy and 22 patients with loco-regional skin metastases. Out of these 47 patients 22 patients developed further distant metastasis. 25 patients (6%) presented with initial distant metastasis. Loco-regional skin metastases occurred in the mean 23 months and loco-regional lymph node metastases within the draining basin in the mean 24 months after SNB. In case of initial systemic metastases these occurred in the mean 31 months after SNB. In patients with loco-regional lymph node metastases 60% of patients had a history of a malignant melanoma on the distant lower extremity. Comparing patients with recurrent tumor manifestation (n=72) and those without recurrent tumor manifestation (n=377) following negative SNB, those with tumor recurrence were significantly older (median 71,5 years vs. 65 years, p=0.05), had significantly more nodular melanomas (62% vs. 44%, p=0.001), significant thicker melanomas (median 3 mm vs. 1.5 mm, p=0.001) and significantly more frequent an ulceration of the primary melanoma (53% vs. 75%, p< 0.001).

**Conclusion:**

Sentinel node biopsy is one of the most important prognostic factors in malignant melanoma. Within a follow-up period of 6 years 16% of patients developed either loco-regional metastasis, distant metastasis or both. Especially patients with malignant melanoma on the distal lower extremity develop lymph node metastases within the draining basin. This might be caused by the passing through the popliteal lymph node station. Popliteal sentinel lymph nodes are not easily to find and popliteal lymph node dissection is not performed. Therefore in this high-risk subpopulation of patients further systemic therapy should be evaluated.

### CD4+ T cell alloresponse on primary human hepatocytes in vitro is associated with MHC II upregulation on hepatocytes and suppressible by Treg

(Abstract ID: 484)

D.E. DeTemple^1^, F. Oldhafer^1^, C. S. Falk^1^, C. Chen-Wacker^1^, C. Figueiredo^1^, M. Kleine^1^, W. Ramackers^1^, K. Timrott^1^, F. Lehner^1^, J. Klempnauer^1^, M. Bock^1^, F. W. R. Vondran^1^

^1^*Medizinische Hochschule Hannover*

**Background:**

Hepatocyte transplantation is an encouraging therapeutic approach for several end-stage liver diseases. Long-term acceptance could not be achieved yet and new methods are needed to ensure reduction of post-transplant cell loss. As increased frequencies of regulatory T cells (Treg) were observed in tolerant patients after liver transplantation, this correlation was investigated.

**Materials and methods:**

Immune reactions induced by primary human hepatocytes (PHH) were analysed. Immunological potential of Treg in mixed lymphocyte and mixed lymphocyte and hepatocyte cultures were examined using peripheral blood mononuclear cells and PHH. Treg were polyclonally expanded to CD4 CD25highCD127low phenotype and added to co-cultures in single-/trans-well setups with/without supplementation of anti-interferon γ (IFN γ). Alloresponses were analysed by flow cytometry.

**Results:**

Hepatocyte induced T cell response was observed as primarily CD4 T cell mediated and associated with IFNγ-induced up-regulation of major histocompatibility complex (MHC) class II on hepatocytes. Experiments with fragmented hepatocytes could rule out indirect antigen presentation. Secretion of inflammatory cytokines accompanied allospecific proliferation. Though CD8 T cells lacked proliferation, early up-regulation of CD69 was observed. Treg supplementation successfully inhibited hepatocyte induced alloresponses. This effect was shown to be primarily cell contact dependent.

**Conclusion:**

The hepatocyte induced alloresponse is CD4 T cell mediated and associated with MHC class II up-regulation on hepatocytes. All responses are suppressible by Treg.

### Ovarian cyst heavier than the patient itself

(Abstract ID: 534)

M. Bialobrzecka^1^, A. Wunsch^1^, P. Schenker^1^, L. Berger^1^, R. Viebahn^1^

^1^*Knappschaftskrankenhaus Bochum Langendreer*

**Background:**

Giant tumours arising from the ovaries are rare events in daily surgical practice. However, they are among the largest known tumours and may present a challenge for removal.

**Materials and methods:**

A 58 years old lady was seen in the emergency room because of beginning haemodynamic instability and impaired alertness. There was a history of progressive abdominal distension over the last 30 years. Due to reasons which could yet not fully elucidated, the patient had denied further evaluation and treatment. Two weeks ago the patient became bedridden. For 1 week she was finding it difficult to eat and drink. There were no other diseases known.

On physical examination there was a marked increase in abdominal circumference. Obvious signs of dehydration and cachexia were present. On ultrasound examination a large hyperechogenic, homogenous intraabdominal mass without boosted vascularisation could be demonstrated. Correspondingly, CT scan showed a huge homogenous tumour (45 x 43 x 50 cms) with a severe compression of IVC, a displacement of the intestine and a bilateral hydronephrosis. The precise nature of this mass was not clear.

The patient was admitted to the ICU and operative treatment was scheduled. Anaesthesia induction was performed in a lateral position with fiber-optic intubation. Surgery took place in 30° left lateral position. Due to intense adhesions to the peritoneum, the cyst was accidentally opened. About 55 to 60 litres of a brown, thick liquid were evacuated. The operation took 12h. An extensive adhesiolysis and a hysterectomy were performed. The transverse, the descending and the sigmoid colon were complete dislocated to the right side. The ovaries could not be identified macroscopically.

**Results:**

Histopathology was reported as a benign endometrial cyst with severe chronic and active inflammation. There were areas of fibrosis and calcification of the cyst wall.

The postoperative period was complicated by 3 intestinal perforations, problems with wound healing and a subcutaneous hematoma. Furthermore a cystitis and bladder emptying disturbances occurred which eventually subsided completely. The patient was transferred to a rehabilitation facility for further mobilization and psychological treatment 8 weeks following the operation in a good general condition.

**Conclusion:**

This case describes an example of a cyst which was even heavier than the rest of the patient.

Although huge endometrial cysts are not uncommon, the size of 45 x 43 x 50 cms and the fact that it developed only 2 kms away from a university clinic in Germany in 2017, makes this case unique. The largest endometrial cyst mentioned in the literature weighed 214 kgs and was resected in Pakistan in 1979. To our knowledge, our case is the second biggest chocolate cyst described so far.

Having in mind the severe cachexia of the patient and her postoperative complications, this case emphasises the importance of maintaining an adequate nutritional status before surgery.

**Picture:**
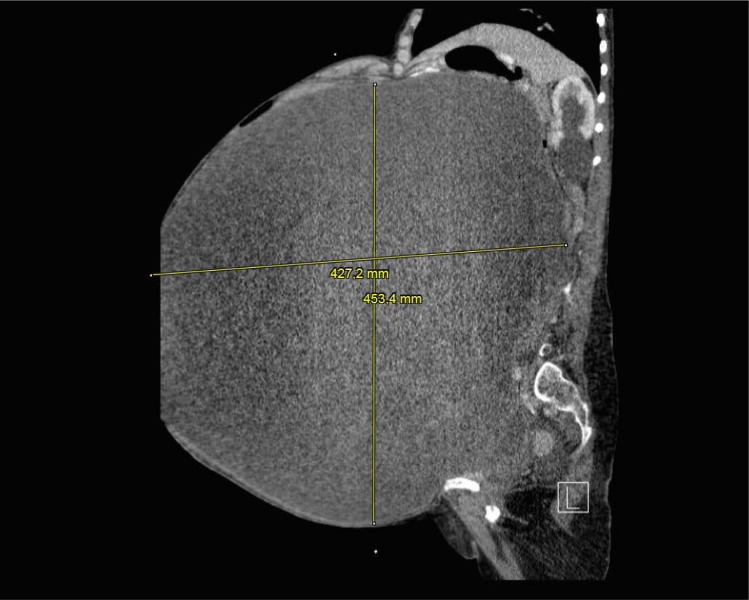
CT scan

### 25 years experience with transpedal lymphangiography in the management of postoperative therapy-refractory lymphatic fistula – preliminar results

(Abstract ID: 636)

C. M. Sommer^1^, F. Pan^1^, T. D. Do^1^, G. M. Richter^2^, H. U. Kauczor^1^, T. Hackert^1^, M. Loos^1^

^1^*Universitätsklinikum Heidelberg*

^2^*Klinikum Stuttgart*

**Background:**

Postoperative therapy-refractory lymphatic fistula is an interdisciplinary therapeutic challenge. In the last years, there is increasing evidence about safety and efficacy of different radiological lymphatic treatments. In this work, we report our 25 year experience on transpedal lymphangiography in the management of postoperative therapy-refractory lymphatic fistula.

**Materials and methods:**

A systematic review of all patients undergoing transpedal lymphangiography in the management of postoperative therapy-refractory lymphatic fistula was performed. For identification of the patients, the search algorithm in our digital database consisted of one MeSH text word: "lymphangiography". Study goals were analyses of patient demographics, technical results, complications, and clinical success.

**Results:**

Between 1993 and 2018, a total of 410 patients underwent transpedal lymphangiography. Of those patients, 371 patients (90.5%) underwent transpedal lymphangiography for the management of postoperative therapy-refractory lymphatic fistula (and 39 patients [9.5%] for the management of idiopathic lymphatic fistula). For the 371 patients, postoperative therapy-refractory lymphatic fistula resulted from a variety of surgical procedures including general surgery, transplantation surgery, vascular surgery, gynecological surgery, urological surgery, visceral surgery, cardiac surgery, and others. Accordingly, there were different locations of the postoperative therapy-refractory lymphatic fistula including neck, thorax, abdomen, pelvis, groin, and lower extremity, The interval between causal surgery and transpedal lymphangiography was 60±169 days. Technical success rate of, amount of injected iodized oil for, and major and minor complication rates of transpedal lymphangiography were 86.8% (322/371 patients), 10.3±4.3 ml, and 0% (0/371 patients) and 0.5% (2/371 patients), respectively. Clinical success rates after transpedal lymphangiography were as follows: Complete response in 37.9% (122/322 patients), partial response in 18.9% (61/322 patients), and clinical failure in 43.2% (139/322 patients). In 20 patients with partial response or clinical failure after transpedal lymphangiography, CT guided sclerotherapy of the lymphatic fistula and/or afferent lymphatic feeders was performed as radiological lymphatic second-line treatment 9±13 days after transpedal lymphangiography. The amount of injected ethanol 95% for and major and minor complication rates of CT guided sclerotherapy were 3.5±2.0 ml and 5.0% (1/20 patient) and 0% (0/20 patients), respectively. Clinical success rates of the 20 patients undergoing CT guided sclerotherapy were as follows: Complete response in 50.0% (10/20 patients), partial response in 20.0% (4/20 patients), and clinical failure in 30.0 (6/20 patients). The interval between causal surgery and discharge for the entire collective (371 patients) was 77±169 days.

**Conclusion:**

Transpedal lymphangiography is feasible, safe and effective for imaging diagnosis and radiological treatment of postoperative therapy-refractory lymphatic fistula. In case of clinical failure after transpedal lymphangiography, radiological lymphatic second-line treatments such as percutaneous sclerotherapy and others (e.g. interstitial embolization and thoracic duct embolization) are indicated to further improve the clinical success. These different and complex radiological lymphatic second-line treatments should be planned specifically on the basis of CT imaging after transpedal lymphangiography and in close consultation with surgical partners to determine a standardized prospective treatment plan.

**Picture:**
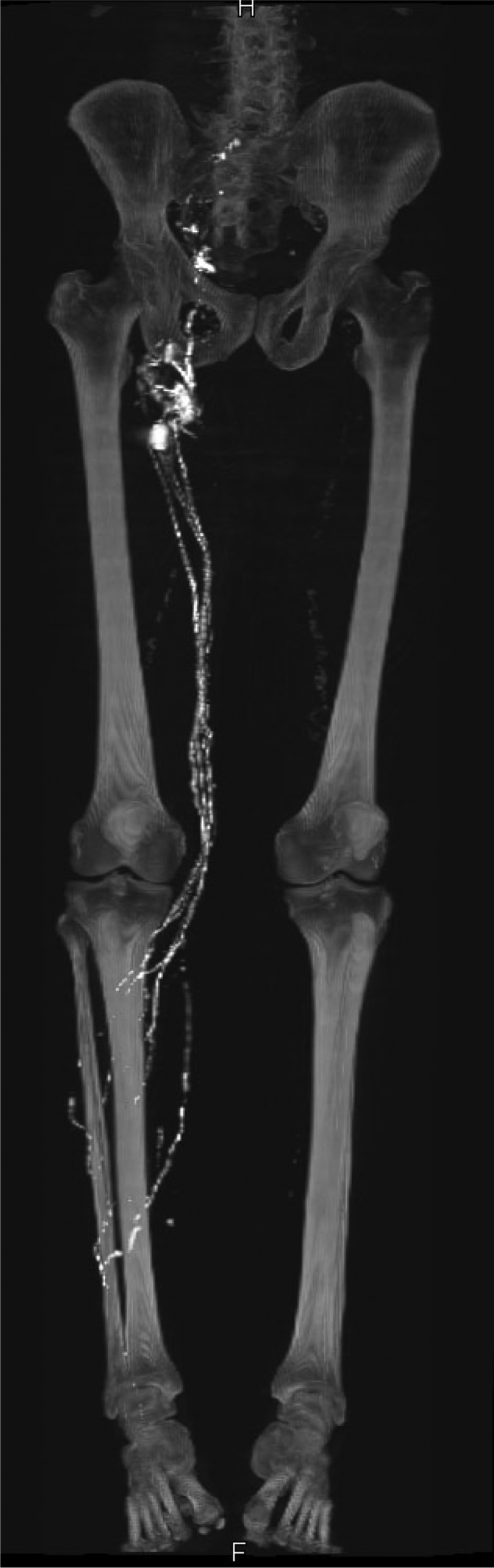
CT imaging after (therapeutic) transpedal lymphangiography for the management of postoperative therapy refractory lymphatic fistula in the right groin.

### Velocity of clinical wound healing without targeted treatment – specified for age, gender and Co-Morbidities

(Abstract ID: 769)

I. Metelmann^1^, F. Podmelle^2^, S. Kindler^2^, R. Rutkowski^2^

^1^*Universitätsklinikum Leipzig*

^2^*Universitätsmedizin Greifswald*

**Background:**

Healing of wounds is different in the course of scarring, in influence of risk factors, functional and aesthetic results and velocity. Research endeavours on wound healing aim to identify means to accelerate wound closure. However, there are no extensive clinical data published referring to the time needed for wound healing, more precisely for re-epithelialization without any targeted and specific treatment. The main concern of this study is to learn about, how many days it takes to cover a patient´s wound with epithelial barrier without any specific treatment and how the healing time is modified by individual condition. Herein, we present results of a clinical trial of STSG donor sites concerning the average velocity of epithelial wound healing depending on various factors.

**Materials and methods:**

Data of the study are part of a phase III multicenter clinical trial with the objective to explore the clinical effectiveness and safety of topical betulin gel in healing of split thickness skin graft donor-site wounds (EudraCT no. 2012-003390-26, EudraCT no. 2012-000777-23). Split thickness skin graft donor site wounds have been divided into two halves. By random decision, one side is treated topically with the substance under investigation, while the other side is left untreated for intra-individual comparison and just covered by an inert standard of care wound dressing. This co-investigation is referring to the outcome of the untreated wound sites only. The assessment was based on photo evaluation by a remote panel of three blinded experts. The protocol is including 198 patients. Statistical analysis was performed with Kaplan-Meier estimates with 95% confidence intervals, plotting cumulative event rate over time to 95% epithelialization (days) for several independent variables and logrank test: X2 and P-value. The observation continued to complete closure of the untreated wound or up to 28 days.

**Results:**

Completion of wound closure is taking at least about 7 days. There are 25% of patients ahead of the group called fast healers with wound closure by day 11. In 50% of the patients wound closure has been completed after 14 days of healing. The final 25% of patients we call late healers presenting wound closure after day 18. Median time to wound closure takes 14 days. Age under 40 years significantly reduces time to wound closure by 2 days while it takes 4 days longer in patients over 60 years of age (P=0). Childbearing potential in females leads to a significant reduction of 4 days (P=0,002). Suffering from cancer or comedication with glucocorticoids lead to a prolongation of 12 and 10 days, respectively (P=0).

**Conclusion:**

A superficial skin wound as artificially provoked by dermatomy for STSG without any targeted treatment starts healing after 7 days, is closed in fast healers after 11 days, needs 14 days for re-epithelialization in 50% of all patients and 18 days in slow healers. Healing is retarded 4 to 12 days extra in patients above 60 years of age, under glucocorticoid medication and suffering from cancer. Healing is getting faster by 2 to 4 days less in younger patients and in pre-menopausal women generally. This follows from the data of 198 patients in 32 hospitals across Europe by common protocol and based upon server-blinded analysis of wound closure by standardized photography.

